# Hypoxia-Induced Osteopontin-Positive Glioma-Associated Macrophages Facilitate Glioma Mesenchymal Transition via NF-κB Pathway Activation

**DOI:** 10.34133/cancomm.0007

**Published:** 2026-01-23

**Authors:** Jingchen Yang, Xuejing Li, Xiaoxue Zhu, Ziwei Li, Xiaoyong Chen, Ruoyu Huang, Mingchen Yu, Bo Han, Tao Jiang, Chuanbao Zhang, Xing Liu

**Affiliations:** ^1^ Department of Molecular Neuropathology, Beijing Neurosurgical Institute, Capital Medical University, Beijing, P. R. China.; ^2^ Department of Neurosurgery, Beijing Tiantan Hospital, Capital Medical University, Beijing, P. R. China.; ^3^ Department of Neuropathology Center, Beijing Neurosurgical Institute, Capital Medical University, Beijing, P. R. China.

## Abstract

**Background:** Hypoxia is a prevalent, characteristic feature of the tumor microenvironment (TME) in glioblastomas (GBMs). As dominant immune cells within the TME, glioma-associated macrophages (GAMs) crucially regulate tumor progression. A comprehensive understanding of the effect of hypoxia on the behavior of GAMs is essential for elucidating the immune landscape and developing innovative therapeutic strategies. This study aimed to elucidate the mechanisms by which GAMs facilitate GBM progression under hypoxic conditions. **Methods:** Transcriptome sequencing, single-cell RNA sequencing, and spatial transcriptomic analyses were performed to explore the correlation between hypoxia and GAMs. Clinical samples were used to validate the findings. The underlying molecular mechanisms were examined via chromatin immunoprecipitation, quantitative real-time polymerase chain reaction, Western blotting analysis, and immunofluorescence assays. The therapeutic effectiveness was assessed via the use of in vivo models. **Results:** A subset of GAMs with elevated osteopontin (OPN) expression accumulates in response to hypoxic stimulation. Hypoxia induces OPN expression in macrophages via the histone 3 lysine 4 trimethylation–WD40 repeat-containing protein 5 (H3K4me3-WDR5) epigenetic axis. These OPN-positive GAMs (OPN^+^ GAMs) enhance the mesenchymal transition in GBMs by secreting OPN into the TME. Mechanistically, OPN activates nuclear factor κB (NF-κB) signaling through cluster of differentiation 44 (CD44), subsequently leading to increased programmed cell death ligand 1 (PD-L1) expression. The inhibition of OPN increased GBM sensitivity to temozolomide (TMZ) in orthotopic models. **Conclusions:** This study revealed the potential mechanism by which hypoxia-induced OPN^+^ GAMs promote the mesenchymal transition in GBM cells and demonstrated the therapeutic potential of targeting OPN to enhance TMZ treatment effectiveness.

## Background

Glioblastoma (GBM) is conventionally regarded as a cold tumor because of its highly immunosuppressive microenvironment, which facilitates immune evasion and limits antitumor immune responses [1]. Increasing evidence has highlighted the tumor microenvironment (TME) as a major driver of therapeutic resistance, substantially contributing to the poor prognosis of GBM patients, whose median survival is approximately 15 months [2]. Although the composition of the TME has been studied, the precise mechanisms governing cellular interactions within this environment are still not fully understood. Glioma-associated macrophages (GAMs) constitute the highest proportion of nontumor cell components in the TME [3]. GAMs directly interact with malignant cells to promote tumor progression [4] and act as architects of the immunosuppressive microenvironment [5,6]. A substantial proportion of GAMs exhibit an M2-like phenotype, which is recognized as a protumorigenic state that impairs antitumor immune surveillance and promotes glioma initiation, invasion, and recurrence [7,8].

Among the key microenvironmental factors, hypoxia is particularly important [9] and is intimately involved in tumor metabolism and the regulation of oncogenic signaling pathways. It has long received widespread attention as a therapeutic target [10–12]. In GBMs, hypoxic stimulation affects various malignant biological processes, such as glioma cell proliferation, differentiation [13], angiogenesis [14], invasion [15], migration [16], and treatment resistance [17], while it also affects the microenvironment and tumor heterogeneity by influencing neighboring components [18–20], such as macrophages [21], endothelial cells [22], fibroblasts [23], and other cells [24]. Moreover, recent studies suggest that nontumor cells also undergo adaptive changes in response to hypoxic stress. They can interact with adjacent cell types [20], including tumor cells [25], thereby influencing tumor growth and progression. As macrophages constitute the most abundant immune population in the TME [26], the function and molecular mechanism of macrophages under hypoxic conditions should be investigated further.

In this study, we focused on the biological changes in GAMs under hypoxic conditions within the glioma microenvironment. Through an integrated analysis of transcriptomic, single-cell, and spatial transcriptomic data, we identified a hypoxia-enriched subcluster of GAMs that is closely associated with the mesenchymal transition of glioma cells. We further explored the potential mechanisms by which this subpopulation promotes tumor progression through functional and molecular biology experiments. Additionally, we identified a potential combination therapeutic strategy to increase the effectiveness of temozolomide (TMZ) treatment.

## Materials and Methods

### Sample collection

The human GBM samples used in this research were obtained from patients who underwent surgical treatment at Beijing Tiantan Hospital, Capital Medical University. Informed consent and clinical sample collection were approved by the Ethics Committee of Beijing Tiantan Hospital, Capital Medical University (KY 2020-093-02).

### Data collection and bioinformatics analysis

A total of 310 and 631 glioma samples for which RNA sequencing (RNA-seq) data and follow-up information were available were downloaded from the Chinese Glioma Genome Atlas (CGGA) database (http://www.cgga.org.cn) [27] and The Cancer Genome Atlas (TCGA) database, projects TCGA-GBM and TCGA-LGG (http://cancergenome.nih.gov/), respectively. The transcriptome data based on spatial sampling of 7 different regions in GBMs (270 cases), including the cellular tumor (CT), hyperplastic blood vessels (HBVs), infiltrating tumor (IT), leading edge (LE), microvascular proliferation (MVP) zone, perinecrotic zone (PNZ), and pseudopalisading cells around necrosis (PAN), were obtained from the IVY GBM atlas (https://glioblastoma.alleninstitute.org/) [28]. The secretory protein list was obtained from the Human Protein Atlas (HPA; https://www.proteinatlas.org/) database. Survival analyses of patients in the CGGA and TCGA databases were performed using the R package survminer, version 0.5.1 (http://cran.rproject.org/web/packages/survminer/), and Kaplan–Meier survival curves were created. The log-rank test was used to assess statistical significance. The hypoxia scores were calculated based on mRNA-based signatures developed in previous studies [29]. Briefly, the median expression of each gene in all the samples was calculated, and each sample was assigned a value of 1 if the expression of the gene was greater than the median or −1 if it was less than the median. This binary matrix was then transposed, and the values were summed across all selected genes to generate a composite hypoxia score for each sample. GAM scores [30], mesenchymal scores, and proneural scores [30] were calculated using the R package gene set variation analysis (GSVA; version 1.20.0). Overall survival curves were plotted using the Kaplan–Meier method. Patients were stratified into different groups based on the optimal cutoff value determined by the surv_cutpoint function from the R package survminer (version 0.4.3).

### Transcriptome sequencing

Total RNA was extracted from cells in the different treatment groups using an Eastep Super Total RNA Extraction Kit (catalog no. LS1040; Promega Biotech). Sequencing was conducted on the NovaSeq platform (Novagene). Raw read counts were normalized, followed by *P* value calculations. The Benjamini–Hochberg correction was applied to adjust for multiple testing and estimate false discovery rate (FDR) values. Genes with adjusted *P* values < 0.05 and |log_2_(fold change)| > 1 were considered differentially expressed genes (DEGs). Gene Ontology (GO) and Kyoto Encyclopedia of Genes and Genomes (KEGG) analyses were performed using the database for annotation, visualization, and integrated discovery (DAVID; https://davidbioinformatics.nih.gov/).

### Single-cell RNA sequencing

The gene expression matrix files of single-cell RNA-seq (scRNA-seq) datasets GSE141383 [31] and GSE117891 [32], as processed in the original studies, were retrieved from the Gene Expression Omnibus (GEO) database (https://www.ncbi.nlm.nih.gov/geo/). The GSE141383 dataset contains 29,451 single-cell transcriptomes of 63 gliomas. The GSE117891 dataset contains 6,148 single-cell transcriptomes of 73 surgical samples from 13 glioma patients and 1 patient with brain metastasis. Downstream analyses were performed using the R package Seurat (version 5.3.0) [33]. Principal components analysis (PCA) was applied for dimensional reduction. Cell clustering was performed with between 1 and 20 dimensions and visualized by uniform manifold approximation and projection (UMAP). Specific cell types were assigned to each cluster using the following established gene expression markers: astrocytes, *GFAP* [32]; oligodendrocytes, *OLIG1* and *OLIG2* [34]; macrophages, *PTPRC* and *CD86* [35]; and T cells, *PTPRC* and *CD3E* [36]. Hypoxia scores were calculated as described previously. The feature plots of gene expression levels were generated using the FeaturePlot function in Seurat. Intercellular crosstalk was analyzed with the CellChat package (version 1.6.1) using the standard protocol described at https://github.com/sqjin/CellChat [37].

scRNA-seq of orthotopic glioma tissues from C57BL/6J mice was performed on a 10× Genomics platform (Shanghai Biotechnology Corp.). Twelve glioma samples derived from C57BL/6J mice across 4 treatment groups (3 mice per group) were included. Raw sequencing data were processed using the Cell Ranger pipeline (version 7.0.1; https://www.10xgenomics.com/support/software/cell-ranger/latest) to generate gene expression matrices. Downstream analyses were performed using the R package Seurat (version 5.3.0). PCA was applied for dimensional reduction. Cell clustering was performed using the top 20 dimensions and visualized using UMAP. Marker genes for each cluster were identified using FindAllMarkers. Cell types were determined by manually comparing cluster-specific markers with canonical cell-type signatures [38–41]. The relative levels of hypoxia score, mesenchymal score, and GAM score were calculated using AddModuleScore. Differential expression analyses of feature genes and scores were performed using the Wilcoxon rank-sum test.

### Spatial transcriptomic analysis

The spatial gene expression data from the 2 human GBM samples used in this study were obtained from 10× Genomics (https://www.10xgenomics.com/datasets/human-glioblastoma-whole-transcriptome-analysis-1-standard-1-2-0) and GSE237183 (https://www.ncbi.nlm.nih.gov/geo/query/acc.cgi?acc=GSE237183) [42]. The data were analyzed using the R package Seurat (version 5.3.0). Briefly, the imported data were first filtered using a complexity filter threshold of 1,000, a mitochondrial filter threshold of 20, and a gene number threshold of 7,000. The filtered data were subsequently normalized and centered for further analysis. The expression levels of feature genes were assessed using AddModuleScore in the Seurat package on the basis of characteristic genes described in previous research [43,44]. For coexpression analysis, the levels of gene expression and score were normalized and the coexpression rate at each spot was defined as the product of the normalized levels of the 2 genes or scores.

Spearman rank correlation coefficient was calculated across all spots using the normalized expression values.

### Cell culture and conditioned medium collection

The established human-derived GBM cell lines U87-MG and LN229, the human-derived monocyte cell lines THP-1 and U937, and the mouse-derived GBM cell line GL261 were purchased from the Cell Resource Center, Peking Union Medical College (which is part of the National Science and Technology Infrastructure, the National Biomedical Cell-Line Resource; http://cellresource.cn). The BNI_2-4, BNI_1-3, and HG9 cell lines are patient-derived primary GBM cell lines isolated from surgical GBM samples at Beijing Tiantan Hospital, Capital Medical University. The BNI_2-4 and BNI_1-3 cell lines are GBM cells that were cultured in suspension in serum-free Dulbecco’s modified Eagle’s medium/nutrient mixture F-12 (DMEM/F12; catalog no. C11330500BT, Thermo Fisher). The medium was supplemented with 2% B-27 minus vitamin A (catalog no. 12587010, Gibco), 20 ng/ml human fibroblast growth factor (FGF; catalog no. 100-18B, Peprotech), and 20 ng/ml human epidermal growth factor (EGF; catalog no. AF-100-15, Peprotech). U87-MG, LN229, HG9, and GL261 cells were cultured in DMEM (catalog no. C11995500BT, Thermo Fisher) supplemented with 10% fetal bovine serum (FBS; catalog no. 10099158, Thermo Fisher). The human monocytic cell lines THP-1 [45] and U937 [46] were cultured in RPMI 1640 medium (catalog no. C11875500BT, Thermo Fisher) supplemented with 10% FBS (catalog no. 10099158, Thermo Fisher). Additionally, 1% penicillin/streptomycin (catalog no. 15140122, Thermo Fisher) was added to all the media. All the cell lines were incubated at 37 °C in a humidified atmosphere containing 5% CO_2_.

Hypoxic culture was performed in an air incubator flushed with a gas mixture of 5% CO_2_ and 94% N_2_ at 37 °C. The final O_2_ pressure of the medium was 1%. The maximum duration of hypoxic culture was 24 h. The conditioned medium (CM) was collected and centrifuged at 120*g* for 10 min at room temperature to remove cell debris for use in subsequent experiments.

### Polarization of THP-1 and U937 cells

Phorbol myristate acetate (PMA; catalog no. HY-18739, MedChemExpress) was added to complete RPMI medium at a concentration of 10 ng/ml and cultured with macrophages for 24 h to induce differentiation. The medium containing PMA was then discarded. The cells were gently washed with phosphate-buffered saline (PBS) to remove residual PMA and nonadherent/dead cells. Complete RPMI medium was added, and the cells were maintained in culture for an additional 48 h. Unstimulated macrophages were polarized with 20 ng/ml interleukin-4 (IL-4; catalog no. HY-P70445, MedChemExpress) and 20 ng/ml IL-13 (catalog no. HY-P70568, MedChemExpress) for another 48 h [47]. Overall, when PMA was used, monocytic cells were differentiated for 72 h and subsequently polarized for 48 h.

### Small interfering RNA transfection and lentivirus transduction

Small interfering RNAs (siRNAs) and the corresponding negative control were purchased from RiboBio Co. Ltd. Opti-MEM reduced-serum medium (catalog no. 51985034, Thermo Fisher) and INTERFERin (catalog no. 101000028, Polyplus-Transfection) were used for siRNA transfection. Cells (1.5 × 10^5^) were seeded into 6-well plates. For suspension cells, the plates were precoated with poly-l-lysine (catalog no. P2100; Solarbio) 1 d prior to cell seeding. The siRNAs, INTERFERin, and Opti-MEM were mixed and incubated at room temperature for 15 min before transfection. The effects of the siRNAs were detected by Western blotting. The siRNAs used in this study are listed in Table S1.

The lentiviruses used in this study were produced by Genechem Co. Ltd. The cells (1.5 × 10^5^) were seeded into 6-well plates precoated with poly-l-lysine and cultured in serum-free media 6 h before transfection. The amount of lentivirus used for infection was calculated according to the multiplicity of infection (MOI) value as follows:

$$\text{Virus volume}\;\left(\mu ,l\right)=\left(\mathrm{MOI}\times \text{cell number}\right)/\text{viral titer}\;\left(\mathrm{TU}/\mu l\right).$$ (1)

The MOIs for different cell lines were determined according to the manufacturer’s guidelines. The MOI for BNI_2-4, BNI_1-3, THP-1, and U937 cells was 20. The MOI for HG9, U87-MG, and LN229 cells was 10. Lentiviruses were transduced into cells for 24 h in the presence of Hi-transG A reagents (catalog no. REVG004, Genechem) according to the manufacturer’s guidelines. The transduced cells were selected with puromycin (2 μg/ml; catalog no. P8230, Solarbio) for at least 1 week, and the transfection efficiency was determined by Western blotting [48]. The short hairpin RNA (shRNA) sequences and all the lentivirus vectors used in this study are listed in Tables S2 and S23, respectively.

### OICR-9429 and OPNi-1 treatment

THP-1 and U937 cells were treated with the WD40 repeat-containing protein 5 (WDR5) inhibitor OICR-9429 (20 μM, catalog no. HY-16993, MedChemExpress) for 24 h during hypoxic culture. All the cells were treated with OPN expression inhibitor 1 (OPNi-1) (50 μM, catalog no. HY-146064, MedChemExpress) for 24 h under hypoxic or normoxic conditions.

### RNA extraction and quantitative real-time PCR

Total RNA was extracted using an Eastep Super Total RNA Extraction Kit (catalog no. LS1040, Promega Biotech) according to the manufacturer’s instructions. cDNAs were synthesized with a RevertAid First Strand cDNA Synthesis Kit (catalog no. K1622, Thermo Fisher) according to the manufacturer’s instructions. Endogenous mRNA levels were determined using PowerUp SYBR Green Master Mix (catalog no. A25742, Thermo Fisher). Actin β (ACTB) expression served as an internal control, and relative expression at the transcript level was calculated using the 2^−ΔΔCt^ method. The polymerase chain reaction (PCR) primers designed and synthesized by Genewiz are listed in Table S4.

### Chromatin immunoprecipitation

Chromatin immunoprecipitation (ChIP) experiments were performed using a ChIP assay kit (catalog no. 17-295, Millipore, Boston, MA, USA) and antibodies according to the manufacturer’s protocol. Briefly, DNA was extracted from 5 × 10^7^ cells and incubated overnight with 10 μg of target antibodies or with normal rabbit immunoglobulin (Ig) A/G. The DNA was then extracted with a ChIP assay kit according to the manufacturer’s guidelines. Quantitative real-time PCR (qPCR) was performed to analyze the precipitated DNA samples after purification and rehydration. The quantification of precipitated DNA was analyzed by calculating the cycle threshold (Ct) value. The Ct value of each precipitated DNA sample was normalized to its corresponding input, and relative enrichment was calculated using the control group as a reference. The ChIP-PCR products were subjected to agarose gel electrophoresis.

### Transwell cell migration assay

Glioma cells were first cultured in CM obtained from the THP-1 and U937 cell lines for 48 h. The cells were then digested, resuspended in culture media supplemented with 2% FBS, and adjusted to a density of 5 × 10^5^ cells/ml. A total of 600 μl of media supplemented with 10% FBS was added to the lower chamber of a 24-well plate, while 100 μl of the cell suspension was added to the upper chamber. After 24 h of routine culture, the wells were washed with PBS twice, fixed with methanol for 5 min, and stained with 0.1% crystal violet (catalog no. C4870, Solarbio) for an additional 5 min. Five random visual fields were observed and photographed under a microscope (Zeiss Axio Observer Z1).

### Cell proliferation assay

Cell proliferation was analyzed using a Cell Counting Kit-8 (CCK-8; catalog no. CK04; Dojindo). Briefly, cells were seeded at a density of 1 × 10^4^ cells per well in 96-well microtiter plates (Corning) and cultured in complete medium. Suspension cells were seeded into 96-well plates precoated with poly-l-lysine. After the cells adhered, the medium was replaced with CM obtained from the THP-1 and U937 cell lines. After 0, 12, 24, 36, and 48 h, the CM was removed, and 100 μl of complete medium with 10 μl of CCK-8 reagent was added to each well and incubated for 2 h. The absorbance was measured at 450 nm using an Infinite 200 enzyme marker (Tecan Trading AG) to assess cell proliferation, with cell-free wells serving as blanks.

### Acridine orange and propidium iodide staining

Cells were harvested and resuspended in PBS at an appropriate concentration. Acridine orange and propidium iodide (AO/PI) staining solution (catalog no. C2019A; Beyotime) was prepared according to the manufacturer’s instructions. Equal volumes of the cell suspension and AO/PI solution were mixed gently and incubated for 5 min at room temperature in the dark. Stained cells were immediately analyzed using an automatic cell fluorescence analyzer (Countstar). The proportion of live cells was automatically recorded.

### Western blotting analysis

Total protein was extracted using radioimmunoprecipitation assay (RIPA) buffer (catalog no. 9806, Cell Signaling Technology) supplemented with phenylmethylsulfonyl fluoride (PMSF; 1 mM, Solarbio) and a phosphatase inhibitor cocktail (catalog no. B15001, Selleck). The protein concentration was quantified with a bicinchoninic acid (BCA) protein assay kit (catalog no. PC0010, Solarbio). The proteins were heated at 95 to 100 °C with sodium dodecyl sulfate–polyacrylamide gel electrophoresis (SDS-PAGE) buffer (catalog no. P1040, Solarbio), separated on a 10% SDS-PAGE gel, and transferred to a polyvinylidene difluoride (PVDF) membrane (Millipore). The membrane was then blocked with 5% skim milk (catalog no. 232100; BD Biosciences) for 1 h at room temperature. The membrane was subsequently incubated with primary antibodies overnight at 4 °C. Secondary antibodies, including goat anti-rabbit IgG H&L [horseradish peroxidase (HRP); catalog no. ab6721, Abcam] and goat anti-mouse IgG H&L (HRP; catalog no. ab6789, Abcam), were chosen according to the species of the primary antibodies and incubated with the membrane for 1 h at room temperature. The proteins in the membrane were detected using an enhanced chemiluminescence protein blotting detection system (Bio-Rad Laboratories). The primary antibodies used and the corresponding concentrations are listed in Table S5.

### Enzyme-linked immunosorbent assay

The levels of OPN in the medium of the THP-1 and U937 cell lines were measured with a Human OPN enzyme-linked immunosorbent assay (ELISA) kit (catalog no. KE00375, Proteintech) according to the manufacturer’s instructions. Briefly, 100 μl of each standard or sample, diluted appropriately in medium, was added to antibody-coated microplate wells and incubated for 2 h at 37 °C. After the wells were washed with wash buffer, a detection antibody was added and incubated for 40 min at 37 °C. The chromogen solution was added to develop the color. After a 15-min incubation at 37 °C, the reaction was stopped by stopping solution, and the absorbance at 450 nm was measured using an Infinite 200 enzyme marker (Tecan Trading AG). The concentrations of OPN were calculated using GraphPad Prism (version 9.0, GraphPad Software Inc.).

### Nuclear-cytoplasmic fractionation

Nuclear and cytoplasmic protein fractions were extracted via the use of a nuclear and cytoplasmic protein extraction kit (catalog no. P0028, Beyotime) according to the manufacturer’s instructions. The protein concentrations were quantified, and the samples were heated at 95 to 100 °C with SDS-PAGE buffer. Protein expression levels were then measured using Western blotting.

### Orthotopic glioma model

Male C57BL/6J or BALB/c nude mice (aged 4 to 6 weeks) were obtained from Beijing Vital River Laboratory Animal Technology Co. Ltd. and housed in the specific pathogen-free (SPF) animal facility of Beijing Neurosurgical Institute. The mice were randomly assigned to the following 4 groups: the control group (treated with PBS), the OPNi-1 group (treated with 0.5 mg/kg OPNi-1, catalog no. HY-146064; MedChemExpress), the TMZ group (treated with 50 mg/kg TMZ, catalog no. HY-17364, MedChemExpress), and the TMZ + OPNi-1 group (treated with 50 mg/kg TMZ and 0.5 mg/kg OPNi-1).

GL261 cells (3 × 10^5^) were stereotaxically injected into C57BL/6J mice as previously described [49,50]. Additionally, 6 × 10^5^ cells (U87-MG cells:THP-1 cells = 2:1) were injected into BALB/c nude mice. Beginning on day 14 since implantation, the mice received daily intraperitoneal injections of the appropriate treatment for 5 d, followed by 2 d without treatment (1-week regimen). Tumor volume was assessed using a PharmaScan 70/16 US (Bruker) nuclear magnetic resonance imaging (MRI) system on day 0 (before treatment) and days 7 and 14 since initial treatment. A survival analysis was conducted, with the survival time defined as the period from tumor implantation to death. All the animal experiments were approved by the Ethics Committee of Beijing Neurosurgical Institute (nos. 202204004 and 202204003).

### Hematoxylin and eosin staining

Tumor tissues were first fixed with 4% paraformaldehyde, dehydrated, embedded in paraffin, and cut to prepare tumor sections. After deparaffinization and rehydration, the tumor sections were stained with hematoxylin (catalog no. C0107, Beyotime) for 5 min, differentiated in 1% acid alcohol for 1 min, and rinsed with water for 20 min. After counterstaining with eosin (catalog no. C0109, Beyotime) for 5 min, the sections were dehydrated, rendered transparent, and sealed. The sections were then observed and photographed with a microscope (Zeiss Axio Observer Z1).

### Immunohistochemistry

The tumor sections were blocked with QuickBlock Blocking Buffer (catalog no. P0222; Beyotime) at room temperature for 15 min after dewaxing and antigen retrieval. The blocking buffer was removed, and the sections were incubated with the primary antibody overnight at 4 °C in a humid chamber. The sections were then washed with PBS containing Tween 20 (PBST) for 3 times and incubated with enzyme-labeled goat anti-mouse/rabbit IgG (catalog no. PV-6000, ZSGB-Bio, Beijing, China) at room temperature for 1 h. Diaminobenzidine (DAB) regent (catalog no. ZLI-9018, ZSGB-Bio) was used for color development. After hematoxylin staining, the slices were dehydrated, rendered transparent, and sealed. The sections were then observed and photographed with a microscope (Zeiss Axio Observer Z1). The results were analyzed using ImageJ software (v1.51; National Institutes of Health) [51]. The H-score, which combines the staining intensity and percentage of positive cells to generate a quantitative score, was calculated to evaluate protein expression levels [52]. All the immunohistochemistry (IHC) results were quantified by an experienced pathologist. The primary antibodies used and the corresponding concentrations are listed in Table S5.

### Immunofluorescence staining

Cells were seeded in 24-well plates with a 14-mm-diameter microscope cover glass on the bottom. For suspension cells, the cover glass was precoated with poly-l-lysine. The cells were fixed with 4% paraformaldehyde for 10 min at room temperature and blocked with QuickBlock Blocking Buffer (catalog no. P0222, Beyotime) at room temperature for another 15 min. The cells on the cover glasses were then incubated with primary antibodies overnight at 4 °C. Secondary antibodies, including goat anti-rabbit IgG H&L (Alexa Fluor 488; ab150077, Abcam), goat anti-mouse IgG H&L (Alexa Fluor 488; ab150113, Abcam), and goat anti-rabbit IgG H&L (Alexa Fluor 594; ab150080, Abcam), were chosen according to the species of the primary antibodies. The secondary antibodies were applied to the cells on the cover glasses at a dilution of 1:500 and incubated for 1 h at room temperature in the dark. The cover glasses were then sealed with ProLong Diamond Antifade Mountant with 4′,6-diamidino-2-phenylindole (DAPI) (catalog no. P36971, Thermo Fisher). The cells were observed and photographed with a fluorescence microscope (Zeiss Axio Observer Z1) and analyzed with ImageJ software. The primary antibodies used and the corresponding concentrations are listed in Table S5.

### Multiplex immunohistochemistry

Multiplex immunohistochemistry (mIHC) staining was performed with an Opal 6-Plex Detection Kit (catalog no. NEL821001KT, AKOYA) according to the manufacturer’s instructions. Briefly, tissue sections underwent 4 sequential staining cycles, each consisting of antigen retrieval, blocking, incubations with primary and secondary antibodies, and opal fluorescence labeling. Following the staining cycles, the sections were mounted using ProLong Diamond Antifade Mountant with DAPI for imaging. The sections were scanned with a NanoZoomer (S60, Hamamatsu) and observed using NDP.view2 software (Hamamatsu).

### Statistical analysis

Data presentation and analysis were conducted using GraphPad Prism (version 9.0, GraphPad Software Inc.) and R (version 4.4.2). Univariate and multivariate Cox proportional hazards regression analyses were performed with the same set of covariates using SPSS Statistics software (version 26.0, IBM Corp.).

Paired or unpaired *t* tests were used to compare the means between the 2 groups. One-way or 2-way analysis of variance (ANOVA) was used for comparisons among multiple groups. Overall survival curves were plotted using the Kaplan–Meier method and compared using the log-rank test. Correlations were assessed with Pearson’s tests. The data are presented as the means ± standard deviations (SDs). The exact number of replicates is indicated in the figure legends. Significance levels for all numerical data are as follows: *P < 0.05, **P < 0.01, and ***P < 0.001.

## Results

### Hypoxia is strongly associated with OPN^+^ GAMs in the glioma microenvironment

**Fig. 1.**
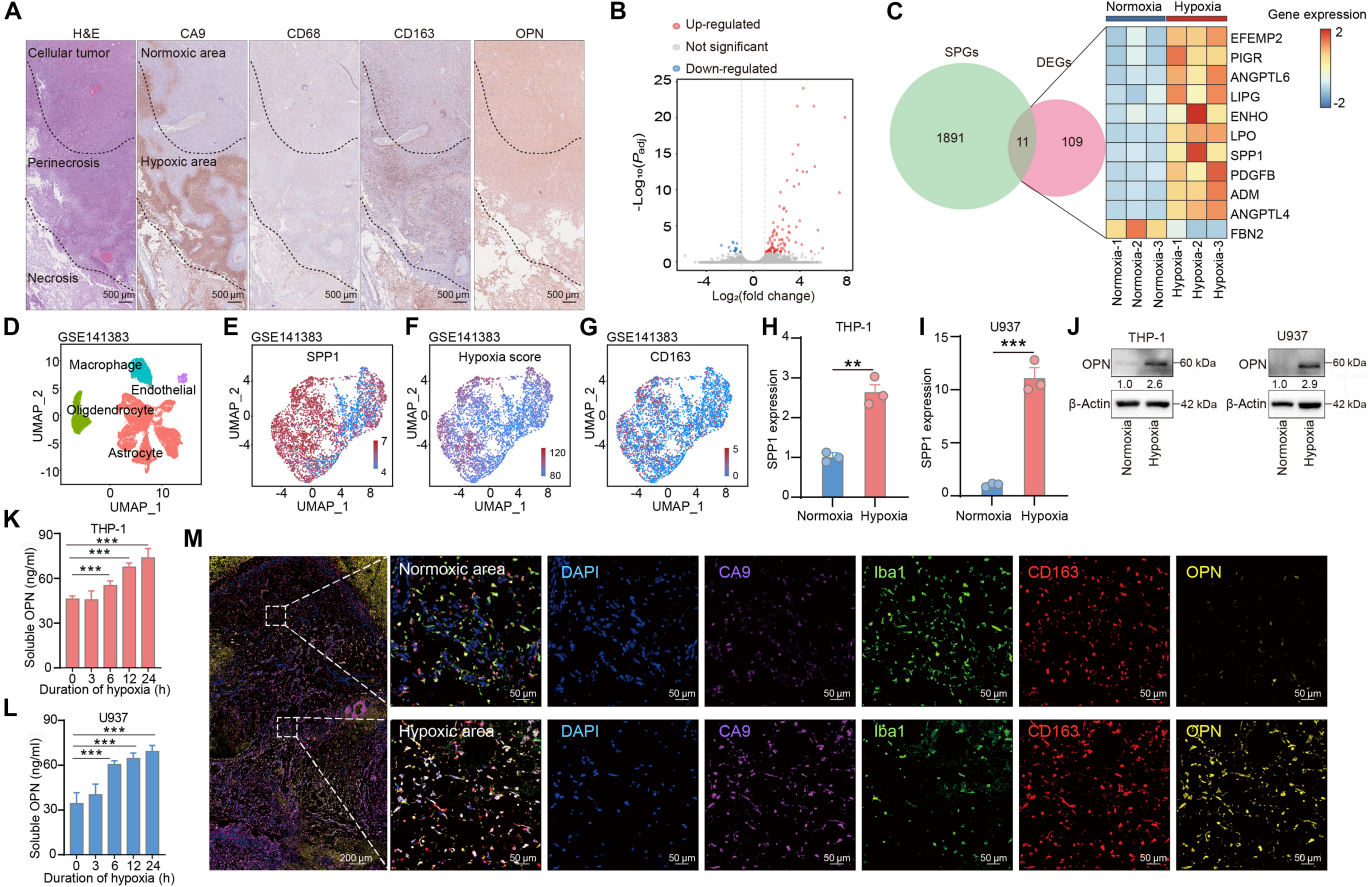
OPN expression increases in macrophages under hypoxic conditions. (A) Representative images of H&E and IHC staining for CA9, CD68, CD163, and OPN in GBM tissues. CA9 expression was used to define hypoxic regions, CD68 and CD163 were used to identify GAMs, and OPN staining indicated the distribution of OPN^+^ GAMs. (B) Volcano plot of the DEGs between THP-1 cells cultured under hypoxic and normoxic conditions, as determined by the transcriptomic analysis. Genes that were significantly up-regulated and down-regulated in response to hypoxia were identified based on *P*_adj_ < 0.05 and |log_2_(fold change)| > 1. Red dots represent up-regulated genes, blue dots represent down-regulated genes, and gray dots indicate nonsignificant changes. (C) Heatmap showing the expression profiles of DEGs encoding secreted proteins, which were identified by intersecting DEGs with secreted protein annotations from the HPA database. Gene expression was normalized and clustered across samples. (D) UMAP plot of all cells from GSE141383 scRNA-seq dataset. (E) UMAP plots showing SPP1 expression of macrophages in the GSE141383 dataset. (F) UMAP plots showing hypoxia scores of macrophages in the GSE141383 dataset. (G) UMAP plots showing CD163 expression of macrophages in the GSE141383 dataset. (H and I) qPCR analysis of SPP1 expression in THP-1 (H) and U937 (I) cells cultured under normoxic and hypoxic conditions for 24 h (*n* = 3 per group). (J) Western blotting showing OPN expression levels in THP-1 and U937 cells cultured under normoxic and hypoxic conditions for 24 h. (K and L) ELISA of OPN levels in the conditioned media of THP-1 (K) and U937 (L) cells exposed to hypoxic conditions for the indicated durations (*n* = 6 per group). (M) Representative images of mIHC staining for CA9 (purple), Iba1 (green), CD163 (red), and OPN (yellow) in GBM tissues. CA9 expression was used to define hypoxic regions, Iba1 and CD163 were used to identify GAMs, and OPN staining indicated the distribution of OPN^+^ GAMs. The data are presented as the means ± SD; **P < 0.01 and ***P < 0.001. OPN, osteopontin; H&E, hematoxylin and eosin; IHC, immunohistochemistry; CA9, carbonic anhydrase IX; CD68, cluster of differentiation 68; CD163, cluster of differentiation 163; GBM, glioblastoma; GAM, glioma-associated macrophage; OPN^+^ GAM, osteopontin-positive glioma-associated macrophage; DEG, differentially expressed gene; HPA, Human Protein Atlas; SPP1, secreted phosphoprotein 1; ELISA, enzyme-linked immunosorbent assay; mIHC, multiplex immunohistochemistry; Iba1, ionized calcium binding adapter molecule 1; qPCR, quantitative real-time PCR; SD, standard deviation; kDa, kilodaltons.

We first performed hematoxylin and eosin (H&E) and IHC staining of serial sections from 50 GBM samples to investigate the relationship between hypoxia and the macrophage distribution in the glioma microenvironment. Tumor regions, including necrotic, perinecrotic, and cellular components, were delineated according to histomorphological criteria [20,42]. The tumor sections were divided into hypoxic and normoxic regions based on CA9 positivity. Total macrophages were identified by cluster of differentiation 68 (CD68) expression, and GAMs were identified by CD68 and CD163 expression (Fig. 1A). Compared with normoxic regions, a higher proportion of GAMs was observed in hypoxic regions, indicating a tendency for GAM enrichment in hypoxic regions (Fig. S1A). These findings are consistent with those of previous reports [42,53].

We simulated the TME in vitro by culturing the human monocytic cell lines THP-1 and U937 under hypoxic (1% O_2_) or normoxic conditions after they were induced to differentiate into a protumor subtype to further explore the effect of hypoxia on GAMs (Fig. S1B and C). THP-1 cells cultured under hypoxic and normoxic conditions were collected for transcriptome sequencing. A total of 120 genes were differentially expressed in cells cultured under hypoxic conditions (*P*_adj_ < 0.05), among which 105 genes were up-regulated and 15 genes were down-regulated (Fig. 1B, Fig. S1D, and Table S6). The results of GO analysis revealed that the DEGs were not only related to hypoxic stimulation in the microenvironment but also closely related to extracellular structure organization and extracellular matrix organization (Fig. S1E).

Given that macrophages primarily influence tumor progression through secreted factors [54–56], we screened secretion-related genes in the HPA database. Eleven DEGs encoding secreted proteins were identified (Fig. 1C). We analyzed the scRNA-seq data from GBM in the GSE141383 dataset to further refine this list (Fig. 1D). Macrophages were selected, and the expression levels of secretion-related DEGs were assessed. The macrophages expressed relatively high levels of secreted phosphoprotein 1 (SPP1) compared with the other 10 genes (Fig. 1E and Fig. S2A to J). In addition, SPP1 exhibited elevated correlation with hypoxia scores and CD163 levels (Fig. 1F and G and Fig. S2K and L). The GSE117891 scRNA-seq dataset was used for validation, and consistent patterns of SPP1 expression were identified (Fig. S3A to P). The increase in the expression of SPP1 and the protein it encodes, osteopontin (OPN), in hypoxic THP-1 and U937 cells was examined using both qPCR (Fig. 1H and I) and Western blotting (Fig. 1J). The concentration of OPN in the media of the THP-1 and U937 cell lines increased in a time-dependent manner (Fig. 1K and L).

We performed AO/PI staining of the THP-1 and U937 cells cultured under hypoxic and normoxic conditions to confirm that the increased OPN levels detected in the media resulted from active OPN secretion by macrophages under hypoxic conditions. The results suggested that hypoxic stress had no significant effect on the viability of macrophages (Fig. S4A and B), thereby excluding the influence of cell death under hypoxic conditions. OPN has been detected in 2 cellular locations: the nucleus and the cytoplasm [57–59]. Therefore, we detected OPN levels in the nucleus and cytoplasm by performing nuclear and cytoplasmic fractionation. In THP-1 and U937 cells cultured under hypoxic conditions, increased OPN expression was detected in both the cytoplasm and nucleus (Fig. S4C and D), suggesting that OPN expression was comprehensively activated by hypoxic stimulation. The scRNA-seq analyses revealed a strong correlation between SPP1 and CD163 expression (Figs. S2L and S3P). Given that CD163 is a key marker of the protumorigenic macrophage subset [60], we intended to further investigate the potential relationship between OPN expression and macrophage polarization toward a protumor phenotype. We inhibited OPN expression in THP-1 and U937 cells by knocking down SPP1 expression and applying OPNi-1, respectively (Fig. S4E and F). Cell viability was not influenced by either the shRNAs or the inhibitor (Fig. S4G and H). The cells were subsequently treated with PMA, IL-13, and IL-4 to induce polarization. qPCR revealed a decrease in the expression of polarization markers in cells with lower OPN expression (Fig. S4I and J). These findings suggest that OPN plays a role in the maintenance of macrophage polarization, which is consistent with the findings of a previous study [61].

We next performed IHC staining of serial sections from 50 GBM patients to assess the clinical relevance of the above findings. Cells coexpressing CD68, CD163, and OPN were defined as OPN-positive GAMs (OPN^+^ GAMs). The proportion of OPN^+^ GAMs was higher in hypoxic regions (Fig. S4K). For further validation, we performed mIHC staining of tumor tissues from an additional 5 GBM patients and found that OPN^+^ GAMs (Iba1^+^ CD163^+^ OPN^+^) were more likely to accumulate in hypoxic areas (Fig. 1M and Fig. S4L). In addition, a spatial transcriptomic data (GSE237183, ZH881_1A bulk) was analyzed. SPP1 and CD163 showed a positively correlated spatial expression pattern (Fig. S4M). Hypoxia scores were then calculated for each spot (Fig. S4N). The spot locations were stratified into high-hypoxia score and low-hypoxia score groups based on the median hypoxia score. We compared the proportions of SPP1^+^ CD163^+^ spots and SPP1^−^ CD163^+^ spots and found that the proportion of SPP1^+^ CD163^+^ spots was higher among high hypoxia score spots, whereas the proportion of SPP1^−^ CD163^+^ spots was higher among low hypoxia score spots (Fig. S4O). These results indicated that the proportion of OPN^+^ GAMs increased in hypoxic regions of GBM. OPN^+^ GAMs are characterized by high OPN secretion and are associated with macrophage polarization.

### Hypoxia induces OPN overexpression via the WDR5-H3K4me3 epigenetic axis

**Fig. 2.**
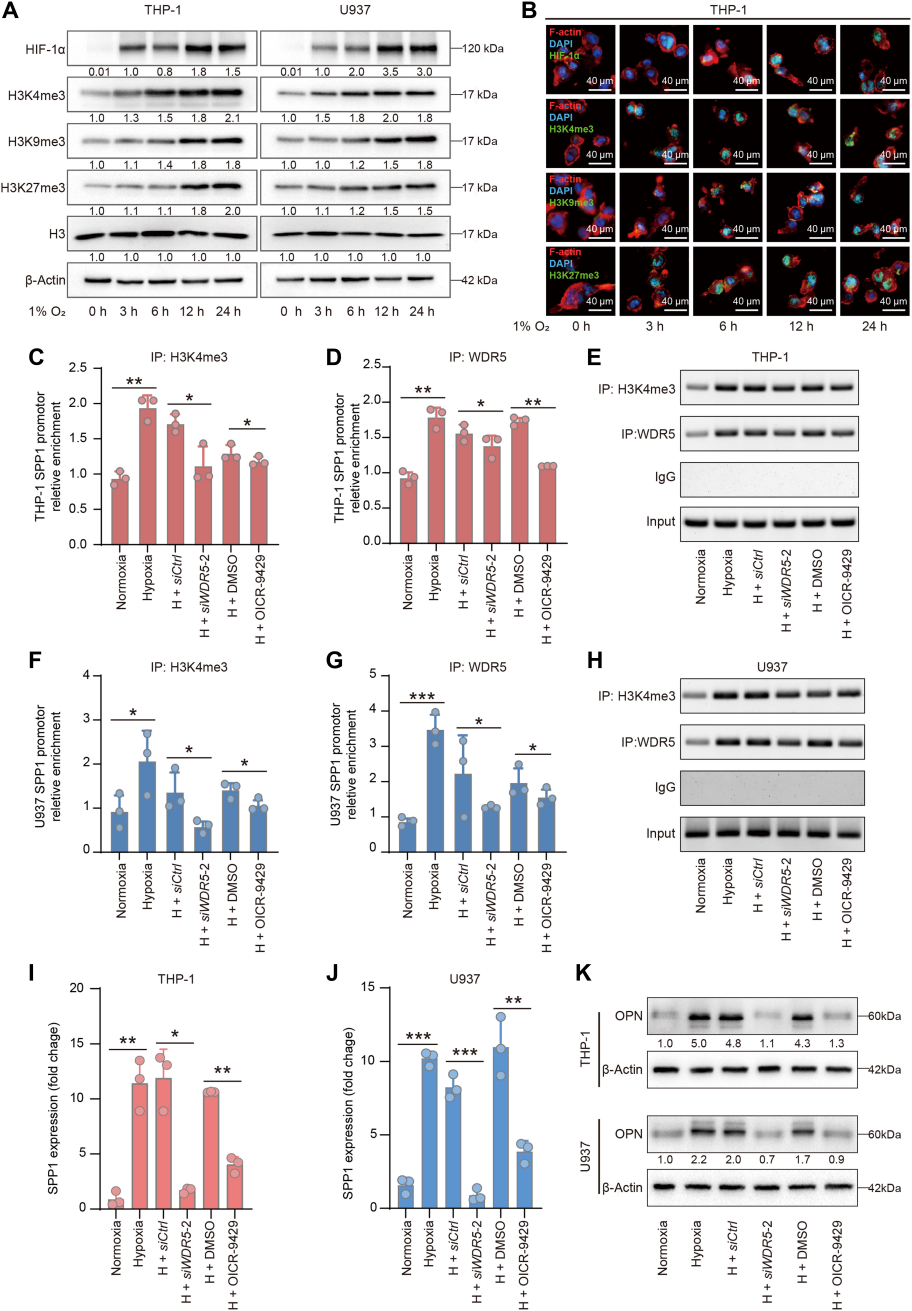
Hypoxia-induced epigenetic regulation contributes to increased OPN expression. (A) Western blotting showing the levels of HIF-1α, H3K4me3, H3K9me3, H3K27me3, and H3 in THP-1 and U937 cells exposed to 1% O_2_ for the indicated durations. (B) IF staining showing the levels of HIF-1α, H3K4me3, H3K9me3, and H3K27me3 in THP-1 cells exposed to 1% O_2_ for the indicated durations. The target proteins are shown in green. DAPI was used to stain the nuclei, and F-actin was labeled to delineate the cytoplasmic region (scale bar = 40 μm). (C) qPCR analysis of H3K4me3 enrichment at SPP1 promoter area in THP-1 cells under indicated treatment conditions (*n* = 3 per group; normoxia, normoxic culture for 24 h; hypoxia, hypoxic culture for 24 h; H + siCtrl, hypoxic culture for 24 h together with transfection of the negative control siRNA; H + siWDR5-2, hypoxic culture for 24 h together with WDR5 knockdown; H + DMSO, hypoxic culture for 24 h together with DMSO treatment; H + OICR-9429, hypoxic culture for 24 h together with OICR-9429 treatment). (D) qPCR analysis of WDR5 enrichment at SPP1 promoter area in THP-1 cells under indicated treatment conditions (*n* = 3 per group). (E) Agarose gel electrophoresis assays showing ChIP-PCR (SPP1 promoter sequence) products from THP-1 cells immunoprecipitated with anti-H3K4me3, anti-WDR5, and IgG antibodies under indicated treatment conditions. (F) qPCR analysis of H3K4me3 enrichment at SPP1 promoter area in U937 cells under indicated treatment conditions (*n* = 3 per group). (G) qPCR analysis of WDR5 enrichment at SPP1 promoter area in U937 cells under indicated treatment conditions (*n* = 3 per group). (H) Agarose gel electrophoresis assays showing ChIP-PCR (SPP1 promoter sequence) products from U937 cells immunoprecipitated with anti-H3K4me3, anti-WDR5, and IgG antibodies under indicated treatment conditions. (I and J) qPCR analysis of SPP1 expression in THP-1 (I) and U937 (J) cells under indicated treatment conditions (*n* = 3 per group). (K) Western blotting showing OPN expression in THP-1 and U937 cells subjected to the indicated treatments. The data are presented as the means ± SD; *P < 0.05, **P < 0.01, and ***P < 0.001. HIF-1α, hypoxia-inducible factor α; H3K4me3, histone 3 lysine 4 trimethylation; H3K9me3, histone 3 lysine 9 trimethylation; H3K27me3, histone 3 lysine 27 trimethylation; IF, immunofluorescence; ChIP, chromatin immunoprecipitation; WDR5, WD40 repeat-containing protein 5; SPP1, secreted phosphoprotein 1; OPN, osteopontin; DMSO, dimethyl sulfoxide; qPCR, quantitative real-time PCR; SD, standard deviation; kDa, kilodaltons.

As one of the most prevalent physicochemical conditions in the TME, hypoxia plays a critical role in regulating epigenetic modifications and gene expression [62]. Hypoxia has been reported to activate histone methylation independent of hypoxia-inducible factors (HIFs) [63–65]. THP-1 and U937 cells were exposed to hypoxia for different periods to investigate whether this mechanism is involved in macrophages. The methylation levels of key histones, including histone 3 lysine 4 trimethylation (H3K4me3), histone H3 lysine 9 trimethylation (H3K9me3), and histone H3 lysine 27 trimethylation (H3K27me3), were assessed. Western blotting and immunofluorescence (IF) assays showed that the H3K4me3, H3K9me3, and H3K27me3 levels increased under hypoxic conditions. These results confirmed that hypoxic stimulation can also induce histone methylation in macrophages (Fig. 2A and B and Fig. S5A to C).

As the only positive transcriptional regulator among the 3 examined histone modifications [62], H3K4me3 is a crucial histone modification that often selectively localizes to target gene promoter regions and downstream transcription start sites to activate target gene transcription [66]. We therefore hypothesized that the increase in H3K4me3 levels under hypoxic conditions may lead to an increase in SPP1 expression. We tested our hypothesis by performing ChIP-PCR targeting H3K4me3 at the SPP1 promoter in the THP-1 cell line. H3K4me3 was enriched at the SPP1 promoter region, and this enrichment increased under hypoxic conditions (Fig. 2C). WDR5, which forms a protein complex with methyltransferases, is essential for H3K4me3 [67]. As expected, the binding of WDR5 to the SPP1 promoter region also increased under hypoxic conditions (Fig. 2D and E). WDR5 knockdown (Fig. S5D) or treatment with the WDR5 inhibitor OICR-9429 disrupted the binding of WDR5 to the SPP1 promoter. As a result, H3K4me3 enrichment at the SPP1 promoter was also reduced. Similar results were obtained in U937 cell line (Fig 2F to H and Fig. S5E). The expression of SPP1 was up-regulated under hypoxic conditions in both cell lines and decreased by down-regulation of WDR5 (Fig. 2I and J). Consistently, OPN expression was also elevated under hypoxic conditions and was attenuated by WDR5 suppression (Fig. 2K). These results explain the key epigenetic mechanism of increased SPP1 expression in macrophages under hypoxic conditions. The inhibition of this epigenetic regulatory axis could serve as an intervention target to reduce the emergence of OPN^+^ GAMs.

### OPN is strongly associated with glioma malignancy and facilitates the mesenchymal transition, proliferation, and migration of glioma cells

**Fig. 3.**
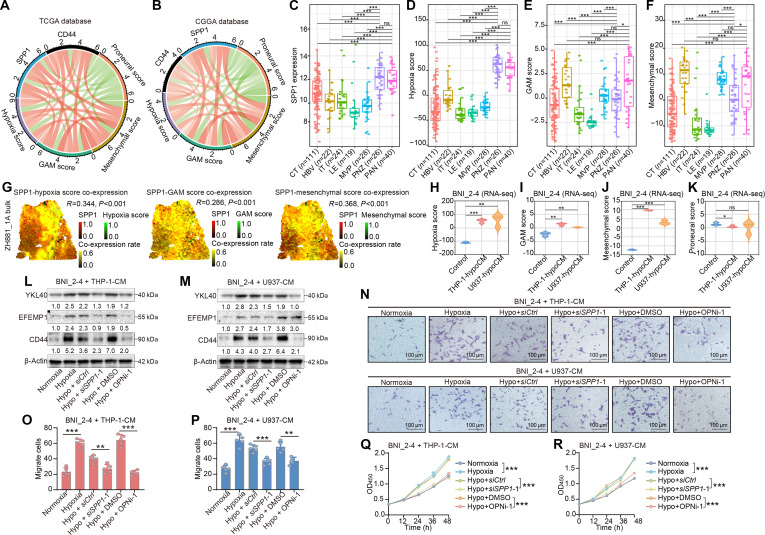
OPN facilitates the mesenchymal transition, migration, and proliferation of glioma cells. (A and B) Chord diagrams showing the correlations among SPP1 expression, CD44 expression, hypoxia score, mesenchymal score, GAM score, and proneural score in glioma samples from TCGA (A) and CGGA (B) datasets (red, positive correlation; green, negative correlation; the scale for each item represents the sum of the absolute values of the correlation coefficient of that item with the other items). (C to F) Distribution of SPP1 expression (C), hypoxia score (D), GAM score (E), and mesenchymal score (F) in samples from the IVY Glioblastoma Atlas dataset. (G) Spatial coexpression rate of SPP1 with hypoxia score, GAM score, and mesenchymal score in ZH_881 1A bulk spatial transcriptomic dataset. (H to K) Violin plots illustrating hypoxia score (H), GAM score (I), mesenchymal score (J), and proneural score (K) generated from RNA-seq data of BNI_2-4 cells cultured with the indicated CM (*n* = 3 per group; control, standard growth medium for macrophages; THP-1-hypoCM, CM obtained from THP-1 cells cultured under hypoxic conditions for 24 h; U937-hypoCM, CM obtained from U937 cells cultured under hypoxic conditions for 24 h). ns, not significant, *P < 0.05, **P < 0.01, ***P < 0.001. (L and M) Western blotting showing the expression levels of representative mesenchymal phenotype-related proteins (YKL40, EFEMP1, and CD44) in BNI_2-4 cells after culture with CM derived from THP-1 (L) and U937 (M) cells under different treatment conditions (normoxia, CM obtained from THP-1 or U937 cells cultured under normoxic condition for 24 h; Hypoxia, CM obtained from THP-1 or U937 cells cultured under hypoxic condition for 24 h; Hypo + siCtrl, CM obtained from siCtrl transfected THP-1 or U937 cells cultured under hypoxic condition for 24 h; Hypo + siSPP1-1, CM obtained from siSPP1-1 transfected THP-1 or U937 cells cultured under hypoxic condition for 24 h; Hypo + DMSO, CM obtained from THP-1 or U937 cells cultured under hypoxic condition for 24 h in the presence of DMSO; Hypo + OPNi-1, CM obtained from THP-1 or U937 cells cultured under hypoxic condition for 24 h in the presence of OPNi-1). (N) Transwell assays showing the migration of BNI_2-4 cells cultured with the indicated CM from THP-1 and U937 cells (scale bar = 100 μm). (O and P) Quantification of migrated BNI_2-4 cells cultured with indicated CM from THP-1 (O) and U937 (P) cells (*n* = 5 per group). **P < 0.01, ***P < 0.001. (Q and R) CCK-8 assays showing the proliferation of BNI_2-4 cells cultured with indicated CM from THP-1 (Q) and U937 (R) cells (*n* = 5 per group). ***P < 0.001. The data are presented as the means ± SD. TCGA, The Cancer Genome Atlas; CGGA, Chinese Glioma Genome Atlas; SPP1, secreted phosphoprotein 1; CT, cellular tumor; HBV, hyperplastic blood vessels; IT, infiltrating tumor; LE, leading edge; MVP, microvascular proliferation; PNZ, perinecrotic zone; PAN, pseudopalisading cells around necrosis; CM, conditioned media; YKL40, chitinase-3 like protein 1; EFEMP1, EGF-containing fibulin extracellular matrix protein 1; CD44, cluster of differentiation 44; DMSO, dimethyl sulfoxide; OPNi-1, OPN expression inhibitor 1; kDa, kilodaltons.

We explored the relationship between SPP1 expression and glioma by performing survival analyses using TCGA and CGGA datasets. Patients were divided into 2 groups based on SPP1 expression. Patients in the high SPP1 group experienced shorter overall survival (Fig. S6A and B). The GBM and lower grade glioma (LGG) subgroups were then analyzed separately, and high SPP1 expression was consistently associated with a poor prognosis for patients in both groups (Fig. S6C to F). In addition, higher SPP1 expression was observed in GBMs [World Health Organization (WHO) grade 4] than in LGGs (WHO grades 2 and 3) (Fig. S7A and B). SPP1 expression was also higher in the mesenchymal subtype than in classical, proneural, and neural subtypes (Fig. S7C and D). High expression of SPP1 was also detected in malignant tumors classified into molecular subtypes, including unmethylated O-6-methylguanine-DNA methyltransferase (MGMT) promoter (Fig. S7E and F), 1p/19q non-codeleted (Fig. S7G and H), and isocitrate dehydrogenase-wildtype (IDH-WT; Fig. S7I and J). We then performed univariate and multivariate Cox analyses with IDH-WT cases from the 2 datasets. As shown in Tables S7 and S8, high SPP1 expression was independently associated with a poor prognosis for GBM patients. We subsequently calculated hypoxia scores, GAM scores, proneural scores, and mesenchymal scores for samples from the 2 databases. Pearson’s correlation analyses revealed that SPP1 expression was significantly positively correlated with the hypoxia score, GAM score, and mesenchymal score but negatively correlated with the proneural score (Fig. 3A and B). Analysis of IVY GBM database showed that SPP1 expression was higher in necrotic area (PNZ and PAN) (Fig. 3C). Moreover, hypoxia score, GAM score, and mesenchymal score showed a similar increasing trend in necrotic areas, whereas the proneural score was comparatively low (Fig. 3D to F and Fig. S8A). The spatial transcriptomic analysis revealed that the spatial distribution of SPP1 expression was positively correlated with that of the hypoxia score, GAM score, and mesenchymal score (Fig. 3G and Fig. S8B). These results indicated a probable correlation between SPP1 expression and the malignant phenotype, especially that of the mesenchymal subtype.

We collected CM from THP-1 and U937 cells cultured under hypoxic or normoxic conditions and cultured the glioma cells with CM to investigate the correlation between OPN expression and the mesenchymal transition in glioma cells. Transcriptome sequencing was performed using BNI_2-4, BNI_1-3, and U87-MG cells cultured with different media for 48 h. Compared with cells cultured in control medium, those cultured in hypoxic CM had increased hypoxia scores (Fig. 3H and Fig. S8C and D), GAM scores (Fig. 3I and Fig. S8E and F), and mesenchymal scores (Fig. 3J and Fig. S8G and H) and reduced proneural scores (Fig. 3K and Fig. S8I and J). BNI_2-4, BNI_1-3, HG9, U87-MG, and LN229 cells were then cultured with different CM for subsequent validation. The expression levels of representative mesenchymal markers (CD44, EFEMP1, and YKL40 [52]) increased in all glioma cells cultured with hypoxic CM. SPP1 knockdown in THP-1 and U937 cells (Fig. S9A) or treatment with OPNi-1 (Fig. S4F) attenuated the effect of hypoxic CM on glioma cells (Fig. 3L and M and Fig. S9B to I). In addition, the migration (Fig. 3N to P and Fig. S10) and proliferation (Fig. 3Q and R and Fig. S11) of glioma cells were increased by the addition of hypoxic CM. The blockade of OPN in macrophages reduced the migration and proliferation of all glioma cell lines cultured with hypoxic CM. We treated glioma cells with OPNi-1 alone to exclude the possibility that the suppression of the malignant phenotype was caused by a direct cytotoxic effect of OPNi-1 on glioma cells. Compared with the dimethyl sulfoxide (DMSO) control group, OPNi-1 did not induce obvious death of glioma cells, indicating that it did not directly kill tumor cells (Fig. S12). These findings support an important role for OPN in promoting the malignant transition of glioma cells.

### CD44 mediates the OPN-induced mesenchymal transition and malignant phenotypes of glioma

**Fig. 4.**
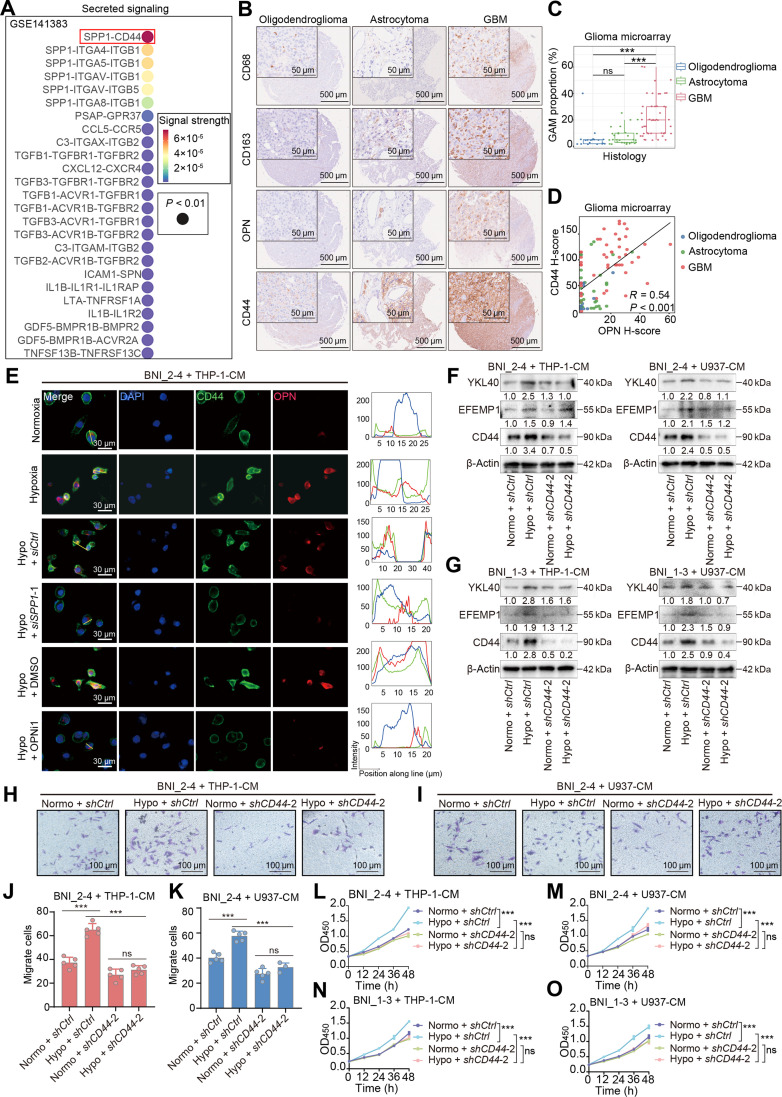
CD44 mediates the protumor effects of OPN on glioma cells. (A) Bubble plot showing ligand–receptor signaling from macrophage to glioma cells in GSE141383 dataset. (B) Representative images of IHC staining for CD68, CD163, OPN, and CD44 in the tissue microarray of glioma samples with different histological subtypes. (C) GAM (CD163^+^ CD68^+^) distribution in glioma tissue microarray samples. ns, not significant and ***P < 0.001. (D) Scatter plot showing the relationship between OPN and CD44 H-scores in a glioma tissue microarray. Each dot represents one sample (blue, oligodendroglioma; green, astrocytoma; red, GBM). (E) IF staining showing the colocalization of OPN (red) and CD44 (green) in BNI_2-4 cells cultured with the indicated CM derived from THP-1 cells (normoxia, CM obtained from THP-1 cells cultured under normoxic condition for 24 h; Hypoxia, CM obtained from THP-1 cells cultured under hypoxic condition for 24 h; Hypo + siCtrl, CM obtained from siCtrl transfected THP-1 cells cultured under hypoxic condition for 24 h; Hypo + siSPP1-1, CM obtained from siSPP1-1 transfected THP-1 cells cultured under hypoxic condition for 24 h; Hypo + DMSO, CM obtained from THP-1 cells cultured under hypoxic condition for 24 h in the presence of DMSO; Hypo + OPNi-1, CM obtained from THP-1 cells cultured under hypoxic condition for 24 h in the presence of OPNi-1). Nuclei were counterstained with DAPI (blue). The yellow line in the first column indicates the line of interest used for intensity analysis. Line intensity profiles on the right display the fluorescence signal distribution along the marked line. (F) Western blotting showing the expression levels of representative mesenchymal phenotype-related proteins (YKL40, EFEMP1, and CD44) in BNI_2-4 cells cultured under the indicated conditions (normo + shCtrl*,* glioma cells that were transinfected with negative control lentivirus cultured in normoxic CM; hypo + shCtrl*,* glioma cells that were transinfected with negative control lentivirus cultured in hypoxic CM; normo + shCD44-2, glioma cells that were transinfected with shCD44-2 lentivirus cultured in normoxic CM; hypo + shCD44-2, glioma cells that were transinfected with shCD44-2 lentivirus cultured in hypoxic CM). (G) Western blotting showing the expression levels of representative mesenchymal phenotype-related proteins (YKL40, EFEMP1, and CD44) in BNI_1-3 cells cultured under the indicated conditions. (H and I) Transwell assays showing the migration of BNI_2-4 cells cultured under the indicated conditions with CM of THP-1 (H) and U937 (I) cells (scale bar = 100 μm). (J and K) Quantification of migrated BNI_2-4 cells cultured under the indicated conditions with CM of THP-1 (J) and U937 (K) cells (*n* = 5 per group). ns, not significant, ***P < 0.001. (L and M) CCK-8 assays showing the proliferation of BNI_2-4 cells cultured under the indicated conditions with CM of THP-1 (L) and U937 (M) cells. ns, not significant, ***P < 0.001. (N and O) CCK-8 assays showing the proliferation of BNI_1-3 cells cultured under the indicated conditions with CM of THP-1 (N) and U937 (O) cells. ns, not significant, ***P < 0.001. The data are presented as the means ± SD. OPN, osteopontin; IHC, immunohistochemistry; CD68, cluster of differentiation 68; CD163, cluster of differentiation 163; GAM, glioma-associated macrophage; OPN^+^ GAM, osteopontin-positive glioma-associated macrophage; KD, knockdown; CM, conditioned media; EFEMP1, EGF-containing fibulin extracellular matrix protein 1; YKL40, chitinase-3 like protein 1; kDa, kilodaltons.

We analyzed intercellular crosstalk from macrophages to glioma cells using scRNA-seq datasets to determine how OPN drives the mesenchymal transition in GBM. Among the ligand–receptor pairs, SPP1-CD44 had a high binding probability in both datasets (Fig. 4A and Fig. S13A). In addition, analyses of TCGA and CGGA datasets revealed a positive correlation between SPP1 and CD44 expression (Fig. 3A and B). Spatial transcriptome data also showed spatial consistency between CD44 and SPP1 expression (Fig. S13B). These findings indicated an association between CD44 and OPN.

CD44 is regarded as a receptor for OPN [68–70] and plays important roles in various pathological processes during tumor progression [71]. We therefore hypothesized that OPN-CD44 might be a key pair that mediates macrophage–glioma cell crosstalk. We validated our hypothesis by performing IHC on tissue microarrays consisting of 96 gliomas with various histological diagnoses (including oligodendroglioma, astrocytoma, and GBM) (Fig. 4B). The results indicated that GBM samples contained higher proportions of GAMs (Fig. 4C). Notably, CD44 and OPN displayed a generally similar distribution pattern among different histological subtypes (Fig. 4D). IF staining glioma cell lines cultured with hypoxic CM derived from THP-1 and U937 cells demonstrated the colocalization of OPN and CD44 on the surface of glioma cells (Fig. 4E and Figs. S13C to E and S14). The inhibition of OPN restricted the binding of OPN and CD44 across all the glioma cell lines. Notably, OPNi-1 did not significantly influence CD44 expression levels in glioma cells, ruling out off-target effects (Fig. S15A).

We knocked down CD44 in the BNI_2-4 and BNI_1-3 cell lines with shRNAs and cultured the cells with CM derived from THP-1 and U937 cells to investigate the role of CD44 in the regulation of the glioma cell mesenchymal transition by macrophages (Fig. S15B to E). Compared with cells transduced with a control shRNA, glioma cells with CD44 knockdown did not show increased expression of mesenchymal markers after exposure to CM (Fig. 4F and G). In addition, hypoxic CM did not promote migration (Fig. 4H to K) or proliferation (Fig. 4L to O) of glioma cells with CD44 knockdown compared with the cells transduced with a control shRNA. Collectively, these findings indicate that CD44 is essential for the OPN-driven mesenchymal transition and malignant phenotypes of glioma cells.

### OPN up-regulates PD-L1 expression in glioma cells by activating the NF-κB pathway

**Fig. 5.**
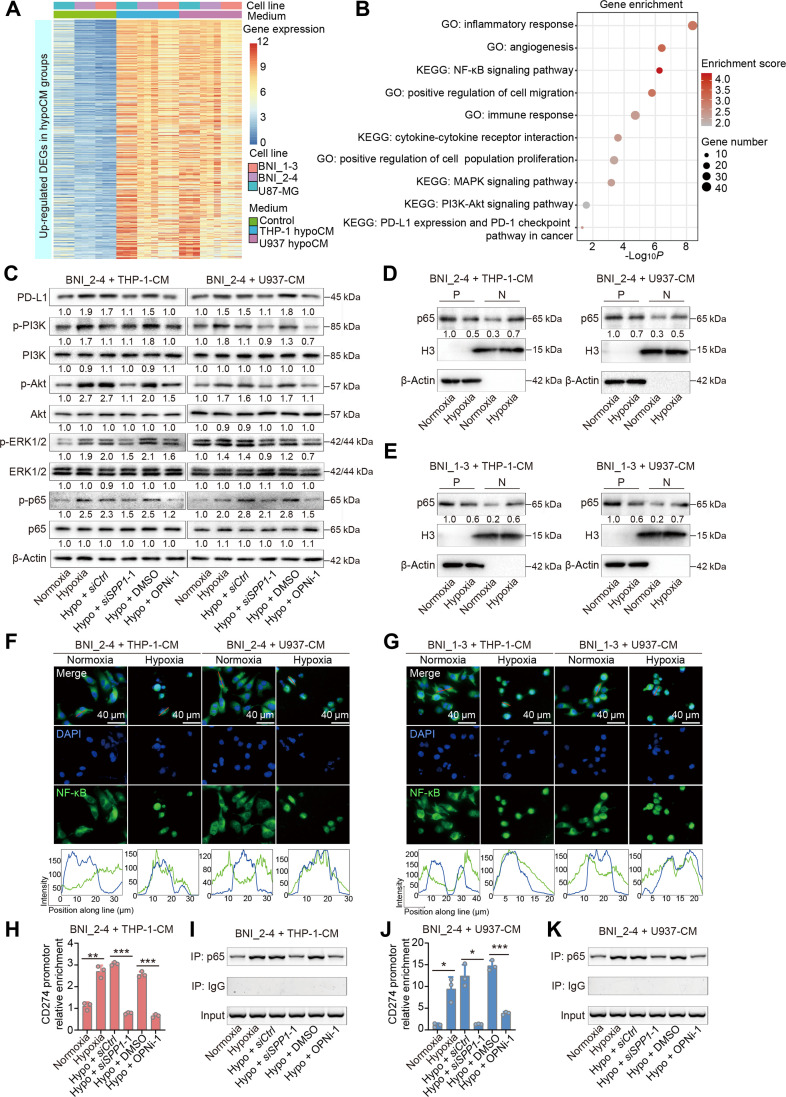
OPN promotes PD-L1 expression in glioma cells by activating the NF-κB signaling pathway. (A) Heatmap showing commonly up-regulated DEGs in BNI_1-3, BNI_2-4, and U87-MG cells treated with the hypoxic CM, as identified by transcriptome sequencing. Gene expression was normalized and clustered across samples. (B) Bubble plot showing the results of GO and KEGG enrichment analyses of commonly up-regulated DEGs in glioma cells cultured with hypoxic CM. (C) Western blotting showing the expression levels of PD-L1 and proteins in the NF-κB signaling pathway in BNI_2-4 cells cultured with the indicated CM (normoxia, CM obtained from THP-1 or U937 cells cultured under normoxic condition for 24 h; Hypoxia, CM obtained from THP-1 or U937 cells cultured under hypoxic condition for 24 h; Hypo + siCtrl, CM obtained from siCtrl transfected THP-1 or U937 cells cultured under hypoxic condition for 24 h; Hypo + siSPP1-1, CM obtained from siSPP1-1 transfected THP-1 or U937 cells cultured under hypoxic condition for 24 h; Hypo + DMSO, CM obtained from THP-1 or U937 cells cultured under hypoxic condition for 24 h in the presence of DMSO; Hypo + OPNi-1, CM obtained from THP-1 or U937 cells cultured under hypoxic condition for 24 h in the presence of OPNi-1). (D and E) Western blotting showing NF-κB (p65 subunit) expression in the cytoplasmic and nuclear fractions of BNI_2-4 (D) and BNI_1-3 (E) cells cultured with normoxic or hypoxic CM derived from THP-1 and U937 cells. β-Actin and H3 were used as cytoplasmic and nuclear loading controls, respectively (P, cytoplasm; N, nuclei). (F and G) IF staining showing the localization of the NF-κB (p65 subunit, green) in BNI_2-4 (F) and BNI_1-3 (G) cells cultured with CM derived from normoxic and hypoxic THP-1 and U937 cells. Nuclei were counterstained with DAPI (blue). The red line in the first row indicates the line of interest used for intensity analysis. The line intensity profiles below display the fluorescence signal distribution along the marked line. (H) qPCR analysis of NF-κB (p65 subunit) enrichment at CD274 promoter area in BNI_2-4 cells cultured with indicated CM derived from THP-1 cells (*n* = 3 per group). (I) Agarose gel electrophoresis showing ChIP-PCR products (CD274 promoter sequence) from BNI_2-4 cells cultured with indicated CM derived from THP-1 cells. Chromatin was immunoprecipitated using anti-NF-κB (p65 subunit) and IgG control antibodies. (J) qPCR analysis of NF-κB (p65 subunit) enrichment at CD274 promoter area in BNI_2-4 cells cultured with indicated CM derived from U937 cells (*n* = 3 per group). (K) Agarose gel electrophoresis showing ChIP-PCR products (CD274 promoter sequence) from BNI_2-4 cells cultured with indicated CM derived from U937 cells. Chromatin was immunoprecipitated using anti-NF-κB (p65 subunit) and IgG control antibodies. The data are presented as the means ± SD. *P < 0.05, **P < 0.01, ***P < 0.001. GO, Gene Ontology; KEGG, Kyoto Encyclopedia of Genes and Genomes; PD-L1, programmed cell death ligand 1; PI3K, phosphatidylinositol kinase 3; p-PI3K, phosphorylated phosphatidylinositol kinase 3; Akt, protein kinase B; p-Akt, phosphorylated protein kinase B; ERK1/2, extracellular signal-regulated protein kinases 1/2; p-ERK1/2, phosphorylated extracellular signal-regulated protein kinases 1/2; NF-κB, nuclear factor κB; p-p65, phosphorylated nuclear factor κB; CM, conditioned media; DMSO, dimethyl sulfoxide; OPNi-1, OPN expression inhibitor 1; kDa, kilodaltons.

We integrated transcriptome sequencing data from BNI_2-4, BNI_1-3, and U87-MG cells cultured with control medium and hypoxic CM to elucidate the molecular alterations occurring in glioma cells following OPN stimulation. We identified the DEGs whose expression was consistently up-regulated in all 3 cell lines cultured with hypoxic CM compared with the respective control medium (Fig. 5A and Table S9). The results of GO and KEGG analyses revealed that the up-regulated DEGs were enriched in nuclear factor κB (NF-κB), mitogen-activated protein kinase (MAPK), and phosphatidylinositol 3-kinase (PI3K)–Akt pathways and were associated with programmed cell death ligand 1 (PD-L1) expression (Fig. 5B). Western blotting analysis revealed that hypoxic CM up-regulated PD-L1 expression and promoted the phosphorylation of key regulators of the NF-κB signaling pathway, including PI3K, Akt, extracellular signal-regulated kinase (ERK1/2), and NF-κB (p65 subunit). SPP1 knockdown in THP-1 and U937 cells or treatment with OPNi-1 attenuated the up-regulation of PD-L1 expression and activation of the NF-κB signaling pathway induced by hypoxic CM (Fig. 5C and Fig. S16).

NF-κB translocates from the cytoplasm to the nucleus when it is activated [72]. Therefore, we performed nuclear-cytoplasmic fractionation of glioma cells cultured with different CM and observed the intracellular distribution of NF-κB. Compared with glioma cells cultured with normoxic CM, those cultured with hypoxic CM presented increased nuclear and decreased cytoplasmic NF-κB (p65 subunit) levels, indicating that hypoxic CM promoted NF-κB nuclear translocation (Fig. 5D and E and Fig. S17A and B). IF staining showed that NF-κB (p65 subunit) translocated from the cytoplasm to the nucleus of glioma cells cultured with hypoxic CM (Fig. 5F and G and Fig. S17C to E). Moreover, the expression of PD-L1 and the phosphorylation of NF-κB signaling pathway-related proteins were not up-regulated by hypoxic CM in glioma cells with CD44 knockdown (Fig. S18). Hypoxic CM did not promote the intracellular translocation of NF-κB in CD44-knockdown glioma cells (Fig. S19).

Upon activation, NF-κB translocates to the nucleus and regulates downstream genes as a transcription factor [73]. We therefore hypothesized that activated NF-κB binds to the promoter region of CD274, the gene encoding PD-L1, thereby up-regulating PD-L1 expression. Consistent with this hypothesis, ChIP-PCR revealed that hypoxic CM generally increased the binding of NF-κB (p65 subunit) to the CD274 promoter region. SPP1 knockdown in THP-1 and U937 cells or treatment with OPNi-1 suppressed this binding, although the statistical robustness of this effect differed among the cell lines 1 (Fig. 5H to K and Fig. S20). These findings indicated that OPN activates NF-κB signaling, thereby up-regulating PD-L1 expression in glioma cells.

### OPN inhibition sensitizes gliomas to TMZ treatment in vivo

**Fig. 6.**
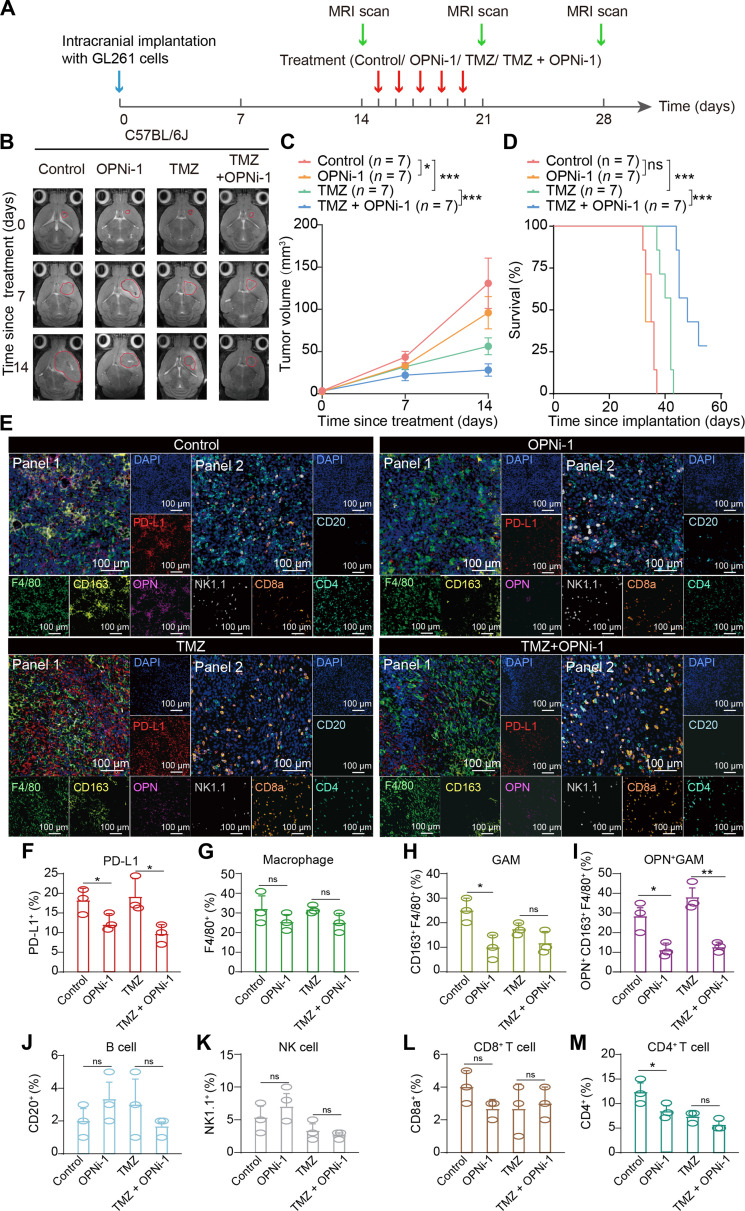
Targeting OPN increases the therapeutic effectiveness of TMZ in a C57BL/6J in vivo glioma model. (A) Schematic illustration of the in vivo experimental design in C57BL/6J mice. The blue arrow represents intracranial implantation; the red arrows represent treatments (control, treatment with PBS; OPNi-1, treatment with OPNi-1 alone; TMZ, treatment with TMZ alone; TMZ + OPNi-1, combined treatment with TMZ and OPNi-1); the green arrows represent MRI scans. (B) Representative MR images showing the intracranial tumor burden in C57BL/6J mice from the indicated treatment groups. (C) Quantification of the tumor volume in C57BL/6J mice from the indicated groups on days 0, 7, and 14 since initial treatment (*n* = 7 mice per group). (D) Kaplan–Meier survival curves of glioma-bearing mice receiving the indicated treatments (*n* = 7 mice per group). (E) Representative mIHC images of glioma tissues from mouse brain sections. Eight markers were divided into 2 staining panels and applied to serial tumor sections to preserve spatial consistency. Panel 1 includes PD-L1, F4/80, CD163, and OPN, and panel 2 includes CD20, NK1.1, CD8a, and CD4. Images for both panels were acquired from matched anatomical regions on adjacent serial sections. (F) Quantification of PD-L1 positivity based on mIHC staining (*n* = 3 per group). (G) Quantification of macrophage (F4/80^+^ cells) proportion based on mIHC staining (*n* = 3 per group). (H) Quantification of GAM (CD163^+^ F4/80^+^ cells) proportion based on mIHC staining (*n* = 3 per group). (I) Quantification of OPN^+^ GAM (OPN^+^ CD163^+^ F4/80^+^ cells) proportion based on mIHC staining (*n* = 3 per group). (J) Quantification of B cell (CD20^+^ cells) proportion based on mIHC staining (*n* = 3 per group). (K) Quantification of NK cell (NK1.1^+^ cells) proportion based on mIHC staining (*n* = 3 per group). (L) Quantification of CD8^+^ T cell (CD8a^+^ cells) proportion based on mIHC staining (*n* = 3 per group). (M) Quantification of CD4^+^ T cell (CD4^+^ cells) proportion based on mIHC staining (*n* = 3 per group). Data are presented as the mean ± SD; *P < 0.05, **P < 0.01. PD-L1, programmed cell death ligand 1; F4/80, mouse EGF-like module-containing mucin-like hormone receptor-like 1; CD163, cluster of differentiation 163; OPN, osteopontin; GAM, glioma-associated macrophage; OPN^+^ GAM, osteopontin positive glioma-associated macrophage; NK, natural killer; CD20, cluster of differentiation 20; SD, standard deviation. Hypoxia induces the emergence of OPN^+^ GAMs through the epigenetic activation of the H3K4me3-WDR5 axis. The secreted OPN promotes the mesenchymal transition and PD-L1 expression in glioma cells via CD44-mediated activation of the NF-κB signaling pathway.

Due to its high degree of heterogeneity and complex immune microenvironment, glioma often presents treatment resistance that is difficult to overcome. We seek to improve the therapeutic effectiveness of TMZ in gliomas by targeting OPN as a method for reprogramming the TME. We first established an orthotopic mouse model of GBM with GL261 in C57BL/6J mice, which provided complete immune function. We subsequently treated the mice with TMZ and/or OPNi-1 (Fig. 6A). MRI showed that treatment with OPNi-1 alone inhibited tumor growth compared with the control treatment. The combination of TMZ and OPNi-1 inhibited tumor growth more effectively than TMZ alone (Fig. 6B and C). The mice treated with TMZ combined with OPNi-1 had prolonged survival compared with those treated with TMZ alone, while treatment with OPNi-1 alone did not prolong survival of the mice compared with the control treatment (Fig. 6D). IHC staining of glioma xenografts showed that CD163 expression was reduced in the OPNi-1-treated group compared with the control group (Fig. S21A and B). Compared with that in the control group, CD44 expression was suppressed by OPNi-1 (Fig. S21A and C). Consistent with the MRI results, TMZ combined with OPNi-1 resulted in the greatest inhibition of tumor growth, as indicated by the lowest Ki67 expression among all the treatment groups. Additionally, OPNi-1 reduce the expression of Ki67 compared with that in the control group (Fig. S21A and D). OPNi-1 inhibited the expression of PD-L1 compared with that in the control group, and the combined therapy of TMZ and OPNi-1 reduced PD-L1 expression as well compared with TMZ treatment alone (Fig. S21A and E). OPNi-1 strongly suppressed OPN expression compared with that in the control group. Similarly, the combination of TMZ and OPNi-1 significantly reduced OPN expression compared with TMZ alone (Fig. S21A and F). For further validation, we established SPP1^+^ THP-1 cells (Fig. S22A) and developed an in situ xenograft model by inoculating U87-MG cells along with OPN^+^ THP-1 cells into BALB/c nude mice (Fig. S22B). These results were consistent with those obtained using the C57BL/6J mouse model (Fig. S22C to K).

As OPNi-1 mainly targeted macrophages, changes in the immune microenvironment were subsequently evaluated in C57BL/6J orthotopic models. We performed serial sectioning of tumor tissues from mice in each treatment group and mIHC for PD-L1, F4/80, CD163, OPN, CD20, NK1.1, CD8a, and CD4 to assess changes in the immune cell composition (Fig. 6E). The quantitative analysis revealed that treatment with OPNi-1 led to decreasing trends in PD-L1 expression (Fig. 6F) and the total number of macrophages (Fig. 6G). Notably, the proportion of GAMs (CD163^+^ F4/80^+^) among total macrophages, as well as the proportion of OPN^+^ GAMs (OPN^+^ F4/80^+^ CD163^+^) among total GAMs, were significantly reduced in tumors from OPNi-1-treated mice (Fig. 6H and I). The proportion of B cells did not show a consistent change across treatment groups (Fig. 6J). Compared with the control treatment, OPNi-1 treatment tended to increase the proportion of natural killer (NK) cells. However, the combined therapy of TMZ and OPNi-1 did not further increase the number of NK cells compared with TMZ alone (Fig. 6K). In contrast, the proportions of CD8^+^ and CD4^+^ T cells tended to decrease in the OPNi-1 group compared with those in the control group. Compared with TMZ alone, the combined therapy tended to reduce the proportion of CD4^+^ T cells, whereas the proportion of CD8^+^ T cells did not change noticeably (Fig. 6L and M). Furthermore, scRNA-seq was conducted on tumors from the mice in the different treatment groups. The cells were clustered and assigned to 16 cell types based on canonical marker genes (Fig. S23A to C). Compared with the control group, OPNi-1 alone reduced the GAM score, mesenchymal score, and the expression of Spp1, Cd44, and Cd274, whereas the hypoxia score was increased in the OPNi-1 group. The B cell marker Ms4a1 and the NK cell marker Klrb1c showed no significant changes in the OPNi-1 group, whereas the CD4^+^ T cell marker Cd4 and the CD8^+^ T cell marker Cd8a were significantly down-regulated. Similar alterations were observed in the TMZ + OPNi-1 group compared with the TMZ group, except that the GAM score and Cd4 expression were increased in the TMZ + OPNi-1 group (Fig. S23D). Overall, these data indicated that the blockade of OPN partially reshaped the GBM microenvironment and potentially increased TMZ sensitivity in a rodent GBM model.

## Discussion

**Fig. 7.**
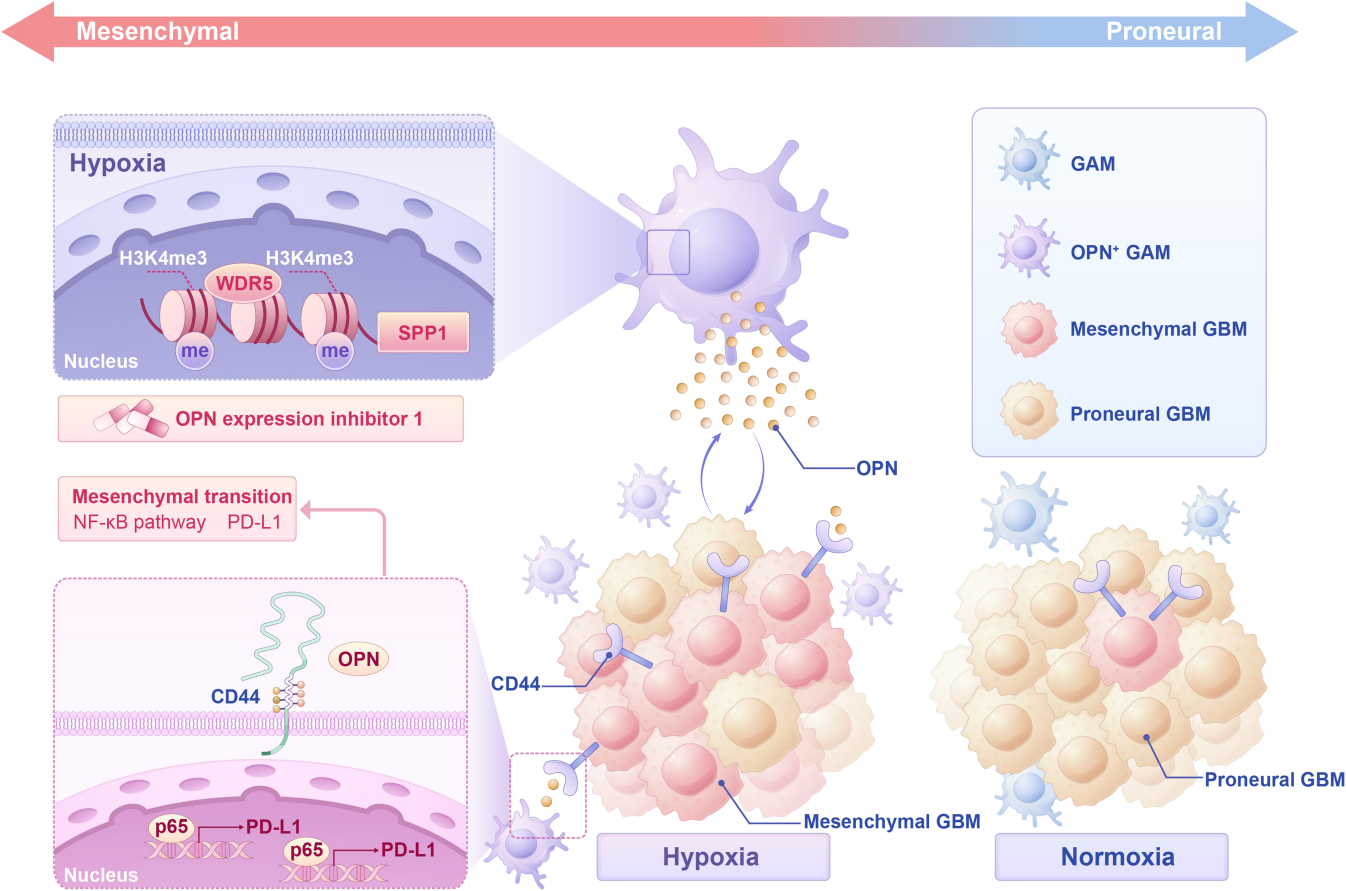
Schematic diagram showing mechanistic summary of OPN^+^ GAM promoting mesenchymal transition of glioma cells under hypoxic conditions. Hypoxia induces the emergence of OPN^+^ GAMs through the epigenetic activation of the H3K4me3-WDR5 axis. The secreted OPN promotes mesenchymal transition and PD-L1 expression in glioma cell axis. The secreted OPN promotes the mesenchymal transition and PD-L1 expression in glioma cells via CD44-mediated activation of the NF-κB signaling pathway. PD-L1, programmed cell death ligand 1; OPN, osteopontin; GAM, glioma-associated macrophage; H3K4me3, histone 3 lysine 4 trimethylation; MES, mesenchymal; PN, proneural; GBM, glioblastoma.

Glioma remains the most common primary malignant tumor of the central nervous system [74], and despite advances in surgical resection, radiotherapy, and chemotherapy, its aggressive nature and therapeutic resistance significantly limit patient outcomes [1,75]. The comprehensive treatment of surgical resection combined with radiotherapy and chemotherapy is currently effective for treating glioma [76]. However, complete resection of GBMs is difficult to achieve because of their aggressive growth. Moreover, GBMs are likely to develop resistance to radiotherapy and chemotherapy, resulting in difficulty in achieving ideal results after surgery [2]. In recent years, the understanding of glioma has gradually developed from focusing on the tumor itself to comprehensively considering the entire TME. Macrophages play a pivotal role in glioma progression [77,78], and their response to hypoxic stimulation within the glioma microenvironment warrants in-depth investigation. By analyzing clinical GBM samples, we observed a spatial correlation between hypoxic regions and GAM enrichment. By integrating scRNA-seq, spatial transcriptomics, and in vitro hypoxia-induced models, we identified a subset of OPN^+^ GAMs that emerged under hypoxic conditions. Mechanistically, hypoxia increased OPN expression in macrophages through H3K4me3-WDR5 epigenetic activity, as confirmed by ChIP assays. Functional studies revealed that OPN activated the NF-κB pathway in glioma cells via an interaction with its receptor CD44, thereby inducing the mesenchymal transition and PD-L1 up-regulation (Fig. 7). Additionally, in an orthotopic glioma model, OPN inhibition not only reshaped the immune microenvironment by reducing the abundance of GAMs and PD-L1 expression but also increased the therapeutic response to TMZ, suggesting a potential combinatorial treatment strategy.

Hypoxia is a common environmental irritant for GBMs and promotes the malignant progression of glioma [79–81]. Recent studies have highlighted a strong association between the composition of the GBM microenvironment and hypoxic regulation. Greenwald et al. [52] proposed the concept of the multilayered organization of GBMs formed under hypoxia. In the hypoxic region, macrophages and stromal glioma cells are spatially closely related. Hypoxia-associated GAMs, particularly monocyte-derived macrophages (MDMs), have been identified as distinct clusters enriched within the hypoxic niches of GBMs. These MDMs are shaped by hypoxia-induced tumor cues to support angiogenesis [20] and impair antitumor immunity [82]. Our findings build upon this concept through the characterization of a hypoxia-induced subset of OPN^+^ GAMs. We described the differential distribution of OPN^+^ GAMs within tumors and elucidated the mechanism by which they directly promote the mesenchymal transition of GBMs.

Encoded by SPP1, OPN regulates tumor growth and progression by binding to receptors [83–85]. In this study, increased OPN secretion from OPN^+^ GAMs were detected, indicating that these cells are functionally active in glioma progression. We also showed that secreted OPN induced the glioma cell mesenchymal transition by binding to its receptor, CD44.

H3K4me3 is among the most important methylation events in cancer development, occurs ubiquitously in eukaryotes at the transcriptional start site, and is usually enriched near the gene promoter [86]. High H3K4me3 levels are associated with a poor prognosis for patients with liver cancer and cervical carcinoma [87,88]. A high H3K4me3 level was correlated with shorter survival of patients with breast cancer [89]. WDR5 is essential for promoting the binding of the methyltransferase complex to the K4-dimethylated H3 tail, as well as for global H3K4me3 in human cells [67]. Consistent with a previous study [90], we identified the epigenetic mechanism of hypoxia-induced OPN up-regulation in GAMs by observing an increase in the binding of the WDR5-H3K4me3 complex to the SPP1 promoter*.*

In vivo experiments showed that the inhibition of SPP1 expression increased the effectiveness of TMZ. Notably, our findings suggested that treatment with OPNi-1 alone may not be enough to suppress glioma growth, but its combination with TMZ provides a synergistic therapeutic effect. These findings are consistent with those of previous studies demonstrating that OPN contributes to therapeutic resistance by promoting the mesenchymal transition and immune evasion [83–85]. Furthermore, the ability of OPN inhibition to reduce PD-L1 expression suggested that this strategy may increase glioma responsiveness to immune checkpoint blockade (ICB) therapies, which is consistent with the findings of a previous study [82].

This study exhibits several limitations. The precise molecular mechanisms linking the OPN-CD44 interaction to NF-κB activation require further investigation. We investigated changes in the immune microenvironment of GBMs following OPN inhibition. However, due to the limited sample size, definitive conclusions could not be derived. In future studies, we aim to dissect the glioma microenvironment by exploring the regulatory interactions among immune cell populations and to identify additional therapeutic opportunities.

## Conclusion

Our study revealed a hypoxia-induced subset of OPN^+^ GAMs that drives glioma malignancy via the secretion of OPN, which binds to CD44 on glioma cells, activating NF-κB signaling and promoting the mesenchymal transition. Targeting OPN increases glioma sensitivity to TMZ treatment, representing a potential therapeutic strategy for GBM. This study provides detailed molecular insights into macrophage–glioma interactions and potential strategies for modulating the glioma immune microenvironment.

## Ethical Approval

The study was approved by the Ethics Committee of Beijing Tiantan Hospital, Capital Medical University (KY 2020-093-02). The clinical samples were obtained with written informed consent from each patient. All the animal experiments were approved by the Ethics Committee of Beijing Neurosurgical Institute (nos. 202204004 and 202204003).

## Data Availability

All the data that support the findings of this study are available from the corresponding authors upon reasonable request.
