# Supplementary material for: Hypoxia-Induced Osteopontin-Positive Glioma-Associated Macrophages Facilitate Glioma Mesenchymal Transition via NF-κB Pathway Activation
Source: Cancer Commun (Lond). 2026 Jan 23;46:0007. doi: 10.34133/cancomm.0007 (PMC12857759; doi:10.34133/cancomm.0007)
Supplement: Supplementary 1 — Figs. S1 to S23 Tables S1 to S9 [file cancomm.0007.f1.docx]

**Supplementary Materials**

**Hypoxia-induced osteopontin-positive glioma-associated macrophages facilitate glioma mesenchymal transition via NF-κB pathway activation**

Jingchen Yang^1,2^, Xuejing Li^1,3^, Xiaoxue Zhu^1,3^, Ziwei Li^2^, Xiaoyong Chen^2^, Ruoyu Huang^2^, Mingchen Yu^1^, Bo Han^2,*^, Tao Jiang^1,2,*^, Chuanbao Zhang^2,*^, Xing Liu^3,*^

^1^Department of Molecular Neuropathology, Beijing Neurosurgical Institute, Capital

Medical University, Beijing, P. R. China.

^2^Department of Neurosurgery, Beijing Tiantan Hospital, Capital Medical University,

Beijing, P. R. China.

^3^Department of Neuropathology Center, Beijing Neurosurgical Institute, Capital

Medical University, Beijing, P. R. China.

^*^Corresponding authors:

Xing Liu; Department of Neuropathology Center, Beijing Neurosurgical Institute, Capital Medical University, Beijing, 100070, P. R. China; E-mail: [liuxing_bjni@mail.ccmu.edu.cn](mailto:liuxing_bjni@mail.ccmu.edu.cn).

Chuanbao Zhang; Department of Neurosurgery, Beijing Tiantan Hospital, Capital Medical University, Beijing, 100070, P. R. China; E-mail: [zhangchuanbao@bjtth.org](mailto:zhangchuanbao@bjtth.org).

Tao Jiang; Department of Molecular Neuropathology, Beijing Neurosurgical Institute, Capital Medical University, Beijing, 100070, P. R. China; Email: [taojiang1964@163.com](mailto:taojiang1964@163.com).

Bo Han; Department of Neurosurgery, Beijing Tiantan Hospital, Capital Medical University, Beijing, 100070, P. R. China; E-mail: cable_han@163.com.


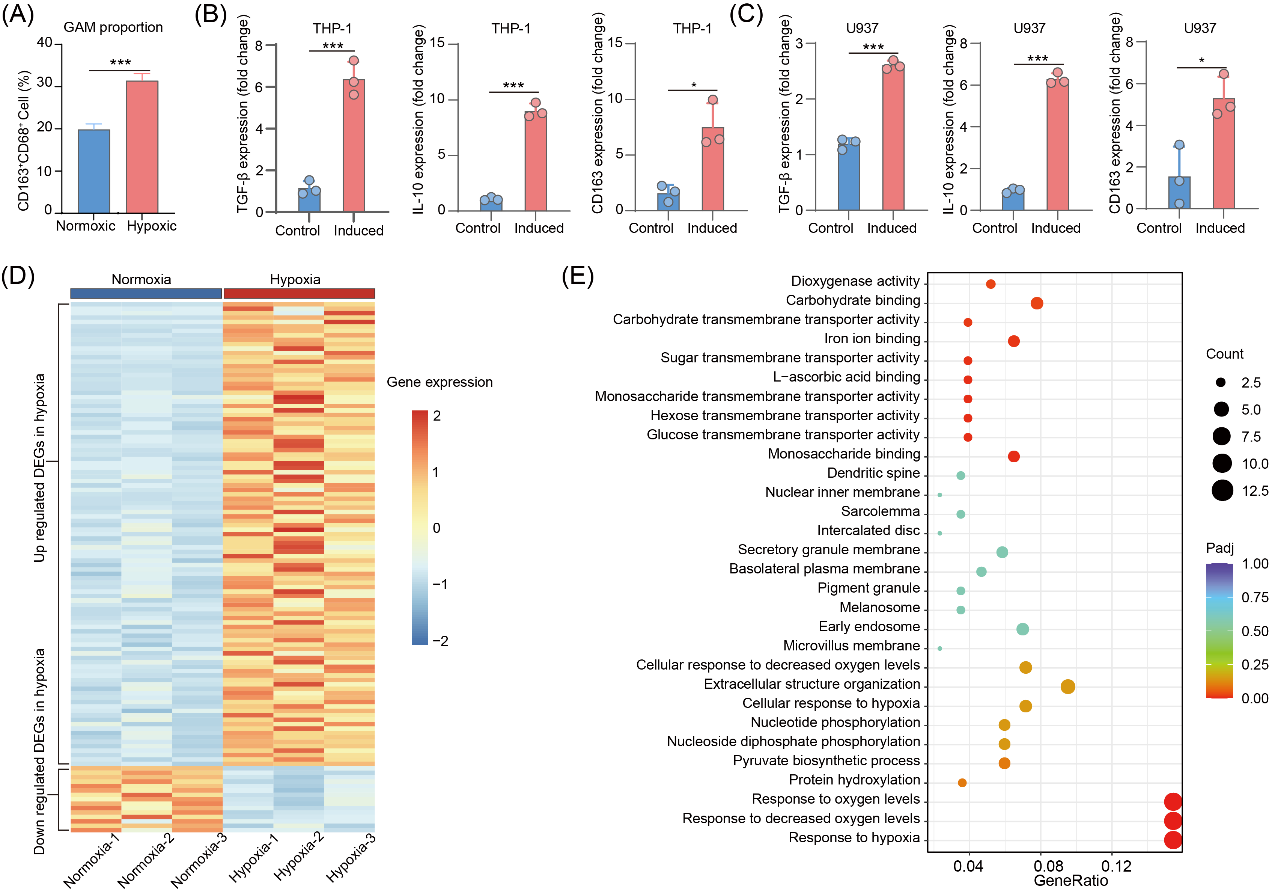


**Supplementary Figure S1.** **In vitro modeling of hypoxia-associated GAMs.**

(A) Quantification of the proportion of GAMs (CD163⁺ CD68⁺ cells) among total macrophages (CD68⁺ cells) in hypoxic and normoxic regions from tumor sections of 50 GBM patients, based on immunohistochemical staining of matched regions from serial sections.

(B) QPCR analysis of representative GAM markers (TGF-β, IL-10, and CD163) in THP-1 cells with or without cytokine induction (*n* = 3 per group).

(C) QPCR analysis of representative GAM markers (TGF-β, IL-10, and CD163) in U937 cells with or without cytokine induction (*n* = 3 per group).

(D) Heatmap showing DEGs between THP-1 cells cultured under hypoxic and normoxic conditions. Gene expression was normalized and clustered across samples.

(E) Bubble plot showing GO enrichment analysis of upregulated DEGs in hypoxia-induced macrophages.

Data are presented as the mean ± SD, **P* < 0.05, ****P* < 0.001.

Abbreviations: GAM, glioma-associated macrophage; qPCR, quantitative real-time PCR; CD68, cluster of differentiation 68; CD163, cluster of differentiation 163; GBM, glioblastoma; TGF-β: transforming growth factor beta; IL-10, Interleukin 10; DEG, differentially expressed gene; GO, gene ontology; SD, standard deviation.


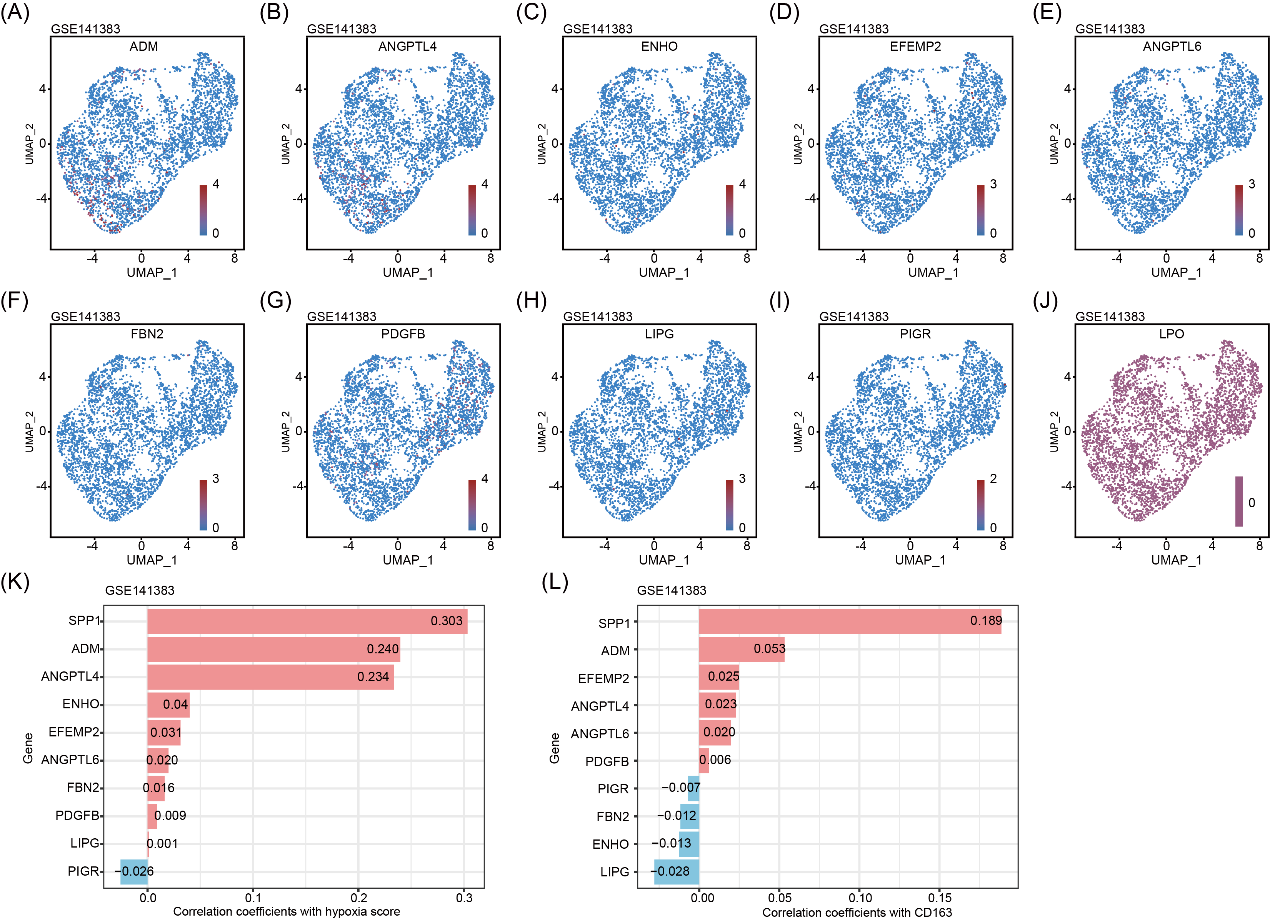


**Supplementary Figure S2. Single cell analysis reveals the correlation between OPN expression and hypoxia conditions.**

(A-J) UMAP plots showing the expression of ADM, ANGPTL4, ENHO, EFEMP2, ANGPTL6, FBN2, PDGFB, LIPG, PIGR, and LPO in the macrophages of GSE141383 database.

(K) Correlations of secreting-related DEGs with hypoxia score in the macrophages of GSE141383 database.

(L) Correlations of secreting-related DEGs with CD163 in the macrophages of GSE141383 database.

Abbreviations: ADM, adrenomedullin; ANGPTL4, angiopoietin-like 4; ENHO, energy homeostasis associated; EFEMP2, EGF containing fibulin extracellular matrix protein; ANGPTL6, angiopoietin-like 6; FBN2, fibrillin 2; platelet derived growth factor subunit B; LIPG, lipase G, endothelial type; PIGR, polymeric immunoglobulin receptor; LPO, lactoperoxidase; CD163, cluster of differentiation 163.


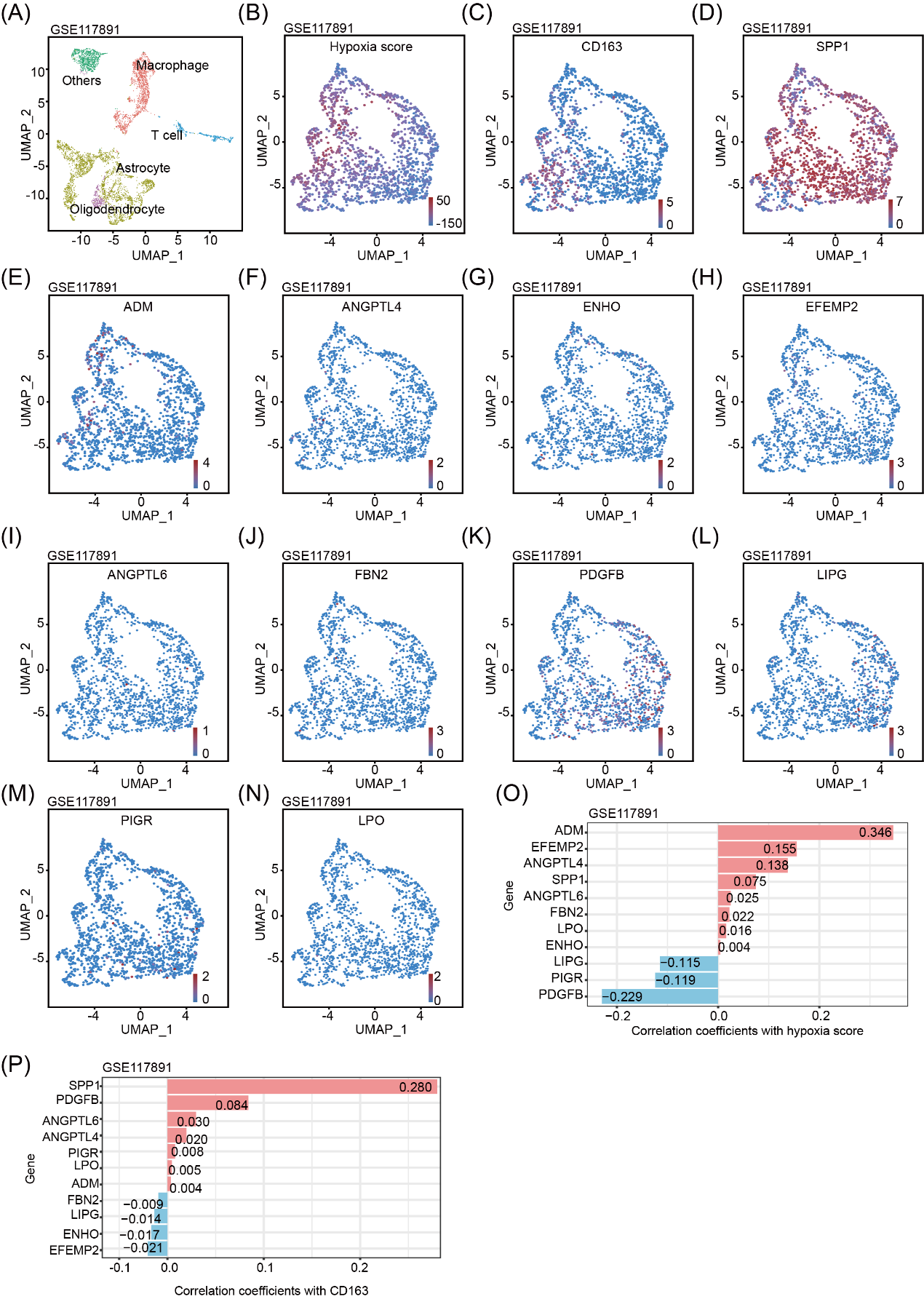


**Supplementary Figure S3. Hypoxia-associated enrichment of SPP1⁺ GAMs in glioma revealed by GSE117891 single-cell dataset.**

(A) UMAP plot of all cells from GSE117891 scRNA-seq dataset.

(B-N) UMAP plots showing the expression level of hypoxia score, CD163, SPP1, ADM, ANGPTL4, ENHO, EFEMP2, ANGPTL6, FBN2, PDGFB, LIPG, PIGR, and LPO in the macrophages of GSE117891 database.

(O) Correlations of secreting-related DEGs with hypoxia score in the macrophages of GSE117891 database.

(P) Correlations of secreting-related DEGs with CD163 in the macrophages of GSE117891 database.

Abbreviations: SPP1, secreted phosphoprotein 1; CD163, cluster of differentiation 163. ADM, adrenomedullin; ANGPTL4, angiopoietin-like 4; ENHO, energy homeostasis associated; EFEMP2, EGF containing fibulin extracellular matrix protein; ANGPTL6, angiopoietin-like 6; FBN2, fibrillin 2; platelet derived growth factor subunit B; LIPG, lipase G, endothelial type; PIGR, polymeric immunoglobulin receptor; LPO, lactoperoxidase.


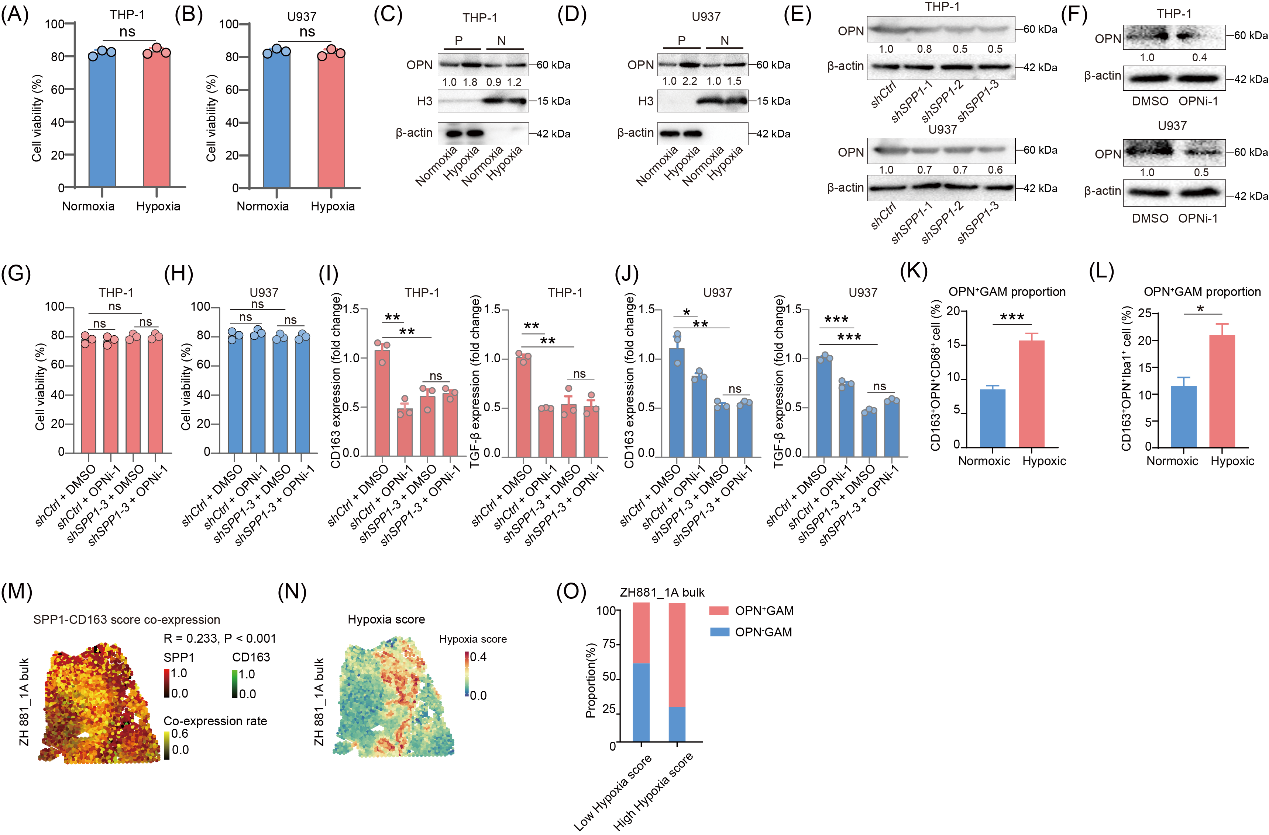


**Supplementary Figure S4. OPN supports macrophage polarization and is spatially enriched in hypoxic regions of gliomas.**

(A-B) Quantification of THP-1 (A) and U937 (B) cell viability under normoxic and hypoxic conditions using AO/PI staining (*n* = 3 per group).

(C-D) Western blotting of OPN expression in cytoplasmic (P) and nuclear (N) fractions of THP-1 (C) and U937 (D) cells cultured under normoxic and hypoxic conditions. β-actin and H3 were used as cytoplasmic and nuclear loading controls, respectively.

(E) Western blotting showing the knockdown effect of shSPP1 on OPN expression in THP-1 and U937 cell lines.

(F) Western blotting showing the effect of OPNi-1 on OPN expression in THP-1 and U937 cells.

(G-H) Quantification of THP-1 (G) and U937 (H) cell viability under indicated treatment conditions (*n* = 3 per group; shCtrl + DMSO*,* cells which were transinfected with negative control lentivirus treated with DMSO; shCtrl + OPNi-1*,* cells which were transinfected with negative control lentivirus treated with OPNi-1; shSPP1-3 + DMSO*,* cells which were transinfected with shSPP1-3 lentivirus treated with DMSO; shSPP1-3 + OPNi-1*,* cells which were transinfected with shSPP1-3 lentivirus treated with OPNi-1).

(I) QPCR analysis of representative GAM markers (CD163 and TGF-β) in THP-1cells induced by IL-4 and IL-13 cells under indicated treatment conditions (*n* = 3 per group).

(J) QPCR analysis of representative GAM markers (CD163 and TGF-β) in in THP-1cells induced by IL-4 and IL-13 cells under indicated treatment conditions (*n* = 3 per group).

(K) Quantification of the proportion of OPN⁺ GAMs (OPN⁺ CD163⁺ CD68^+^) among total GAMs (CD163⁺ CD68⁺) in hypoxic and normoxic regions from tumor sections of 50 GBM patients, based on immunohistochemical staining of matched regions from serial sections.

(L) Quantification of the proportion of OPN^+^ GAMs (OPN^+^ CD163⁺ Iba1⁺) among GAMs (CD163^+^ Iba1⁺) in hypoxic and normoxic regions, based on mIHC staining in tumor sections from 5 GBM patients.

(M) Spatial co-expression rate of SPP1 with CD163 in ZH_881 1A bulk spatial transcriptomic dataset.

(N) Spatial mapping of hypoxia score in ZH_881 1A bulk spatial transcriptomic dataset.

(O) Bar plot showing the proportion of OPN⁺ CD163⁺ spots and OPN⁻CD163⁺ spots in the spots with high or low hypoxia scores in in ZH_881 1A bulk spatial transcriptomic dataset.

Data are presented as the mean ± SD, ns, not significant, **P* < 0.05, ***P* < 0.01, ****P* < 0.001.

Abbreviations: OPN, osteopontin; CD68, cluster of differentiation 68; CD163, cluster of differentiation 163; TGF-β: transforming growth factor beta; GBM, glioblastoma; GAM, glioma-associated macrophage; OPN^+^ GAM, osteopontin positive glioma-associated macrophage; SPP1, secreted phosphoprotein 1; mIHC, multi-immunohistochemistry; Iba1, ionized calcium binding adapter molecule 1; qPCR, quantitative real-time PCR; KD, knockdown; SD, standard deviation; kDa, kilodalton.


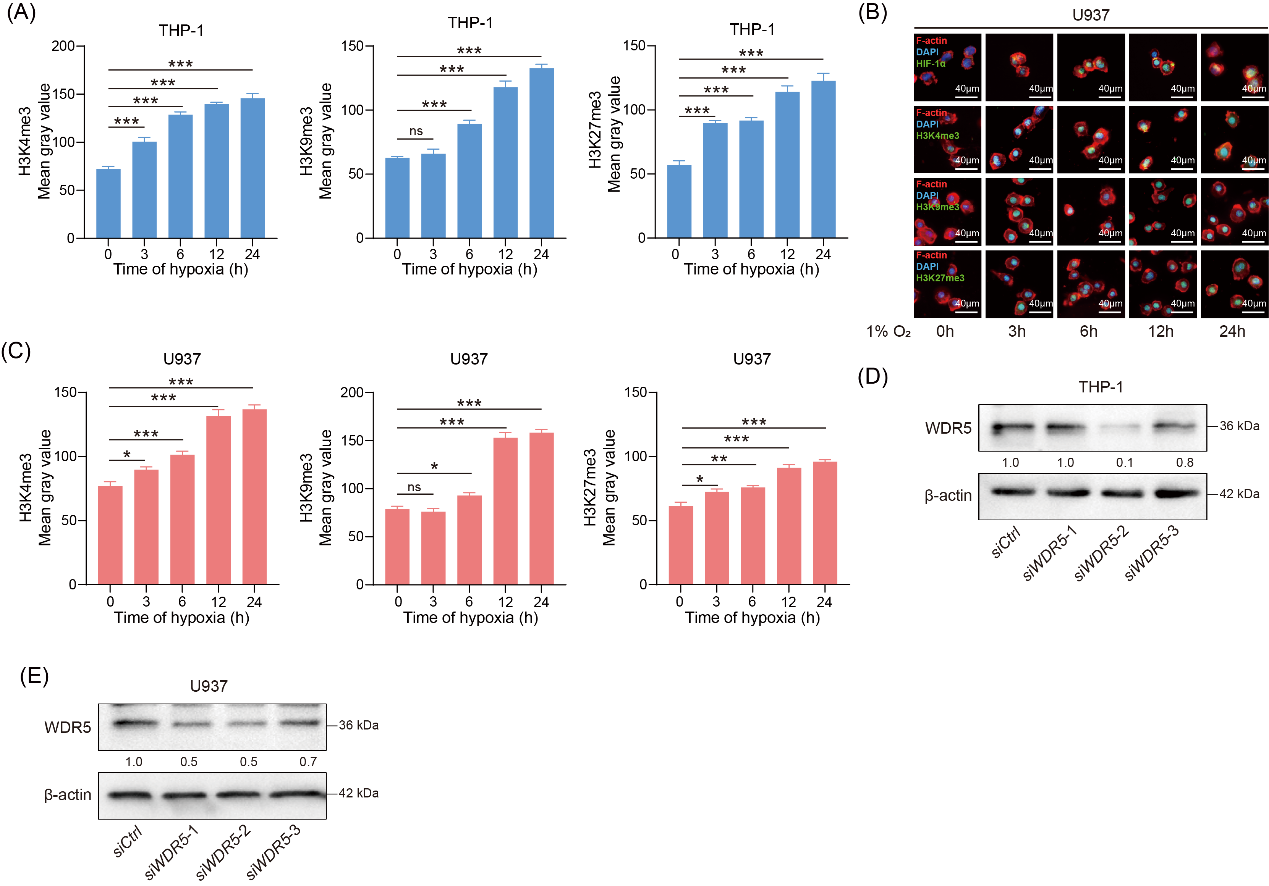


**Supplementary Figure S5. Hypoxia enhances histone trimethylation in macrophages.**

(A) Quantification of fluorescent intensity for H3K4me3, H3K9me3, and H3K27me3 in THP-1 cells exposed to 1% O₂ for the indicated durations (*n* = 5 per group).

(B) IF staining showing the levels of HIF-1α, H3K4me3, H3K9me3, and H3K27me3 in U937 cells exposed to 1% O₂ for the indicated durations. The target proteins are shown in green. DAPI was used to stain the nuclei, and F-actin was labeled to delineate the cytoplasmic region (scale bar = 40 μm).

(C) Quantification of fluorescent intensity for H3K4me3, H3K9me3, and H3K27me3 in U937 cells exposed to 1% O₂ for the indicated durations (*n* = 5 per group).

(D-E) Western blotting showing knockdown effect of WDR5 in THP-1 (D) and U937 (E) cell lines.

Data are presented as the mean ± SD, ns, not significant, **P* < 0.05, ***P* < 0.01, ****P* < 0.001.

Abbreviations: H3K4me3, histone 3 lysine 4 trimethylation; H3K9me3, histone 3 lysine 9 trimethylation; H3K27me3, histone 3 lysine 27 trimethylation; WDR5, WD40 repeat-containing protein 5; SD, standard deviation.


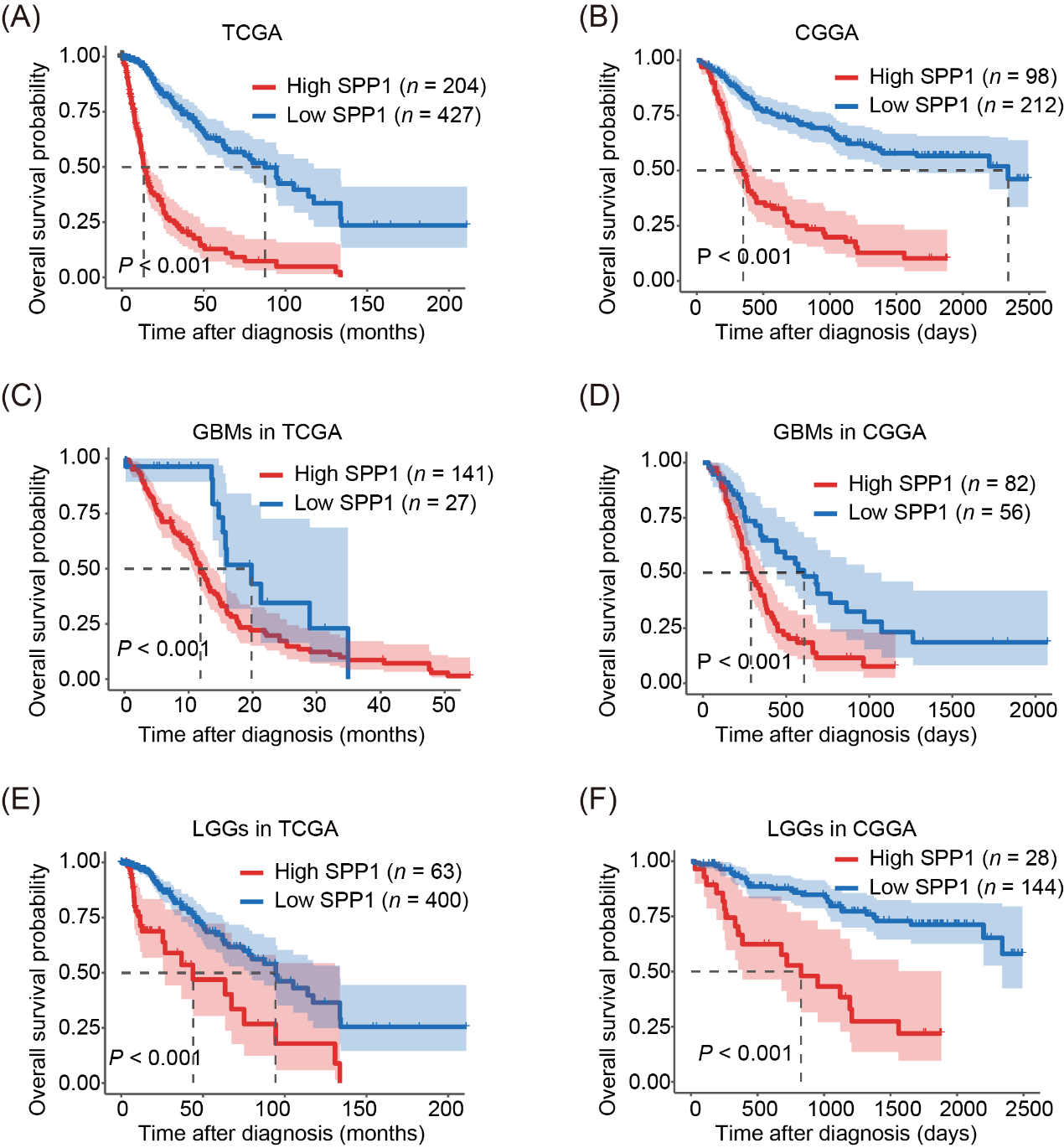


**Supplementary Figure S6. SPP1 expression is related to overall survival of glioma patients.**

(A) Kaplan-Meier survival analysis of overall survival in TCGA glioma patients stratified by SPP1 expression.

(B) Kaplan-Meier survival analysis of overall survival in CGGA glioma patients stratified by SPP1 expression.

(C) Kaplan-Meier survival analysis of overall survival in GBM patients from TCGA dataset stratified by SPP1 expression.

(D) Kaplan-Meier survival analysis of overall survival in GBM patients from CGGA dataset stratified by SPP1 expression.

(E) Kaplan-Meier survival analysis of overall survival in LGG patients from TCGA dataset stratified by SPP1 expression.

(F) Kaplan-Meier survival analysis of overall survival in LGG patients from CGGA dataset stratified by SPP1 expression.

Patients were divided into high and low SPP1 expression groups based on the optimal cutoff value determined by the surv_cutpoint function from the survminer R package. Statistical significance was evaluated using the log-rank test.

Abbreviations: TCGA, the Cancer Genome Atlas; CGGA: Chinese Glioma Genome Atlas; GBM, glioblastoma; LGG, lower grade glioma; SPP1 secreted phosphoprotein 1.


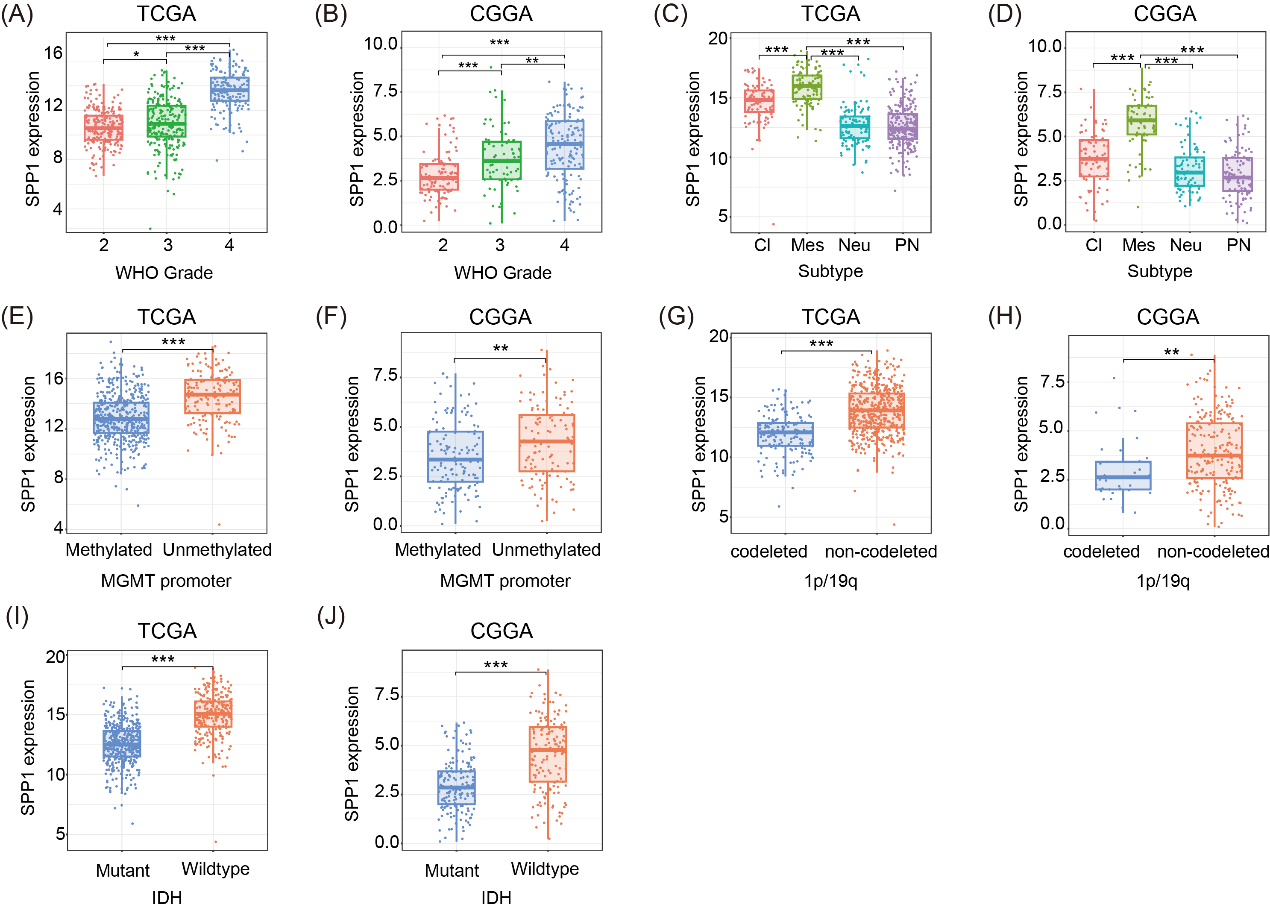


**Supplementary Figure S7. SPP1 showed higher expression level in malignant molecular phenotypes.**

(A) SPP1 expression distribution in the glioma patients of TCGA dataset stratified by WHO grade (WHO 2, *n* =224; WHO 3, *n* =246; WHO 4, *n* =168).

(B) SPP1 expression distribution in the glioma patients of CGGA dataset stratified by WHO grade (WHO 2, *n* = 105; WHO 3, *n* = 67; WHO 4, *n* = 138).

(C) SPP1 expression distribution in the glioma patients of TCGA dataset stratified by molecular subtype (Cl, classical, *n* = 89; MES, mesenchymal, *n* = 100; Neu, neural, *n* = 104; PN, proneural, *n* = 226).

(D) SPP1 expression distribution in the glioma patients of CGGA dataset stratified by molecular subtype (Cl, classical, *n* = 70; MES, mesenchymal, *n* = 65; Neu, neural, *n* = 76; PN, proneural, *n* = 99).

(E) SPP1 expression distribution in the glioma patients of TCGA dataset stratified by MGMT promoter methylation status (methylated, *n* = 438; unmethylated, *n* = 154).

(F) SPP1 expression distribution in the glioma patients of CGGA dataset stratified by MGMT promoter methylation status (methylated, *n* = 134; unmethylated, *n* = 110).

(G) SPP1 expression distribution in the glioma patients of TCGA dataset stratified by 1p/19q codeletion status (codeleted, *n* = 152; non-codeleted, *n* = 472).

(H) SPP1 expression distribution in the glioma patients of CGGA dataset stratified by 1p/19q codeletion status (codeleted, *n* = 36; non-codeleted, *n* = 218).

(I) SPP1 expression distribution in the glioma patients of TCGA dataset stratified by IDH mutation status (mutant, *n* = 386; wildtype, *n* = 236).

(J) SPP1 expression distribution in the glioma patients of CGGA dataset stratified by IDH mutation status (mutant, *n* = 160; wildtype, *n* = 150).

**P* < 0.05, ***P* < 0.01, ****P* < 0.001.

Abbreviations: TCGA, the Cancer Genome Atlas; GBM, glioblastoma; LGG, lower grade glioma; CGGA, Chinese Glioma Genome Atlas; SPP1, secreted phosphoprotein 1; MGMT, O-6-methylguanine-DNA methyltransferase; IDH, isocitrate dehydrogenase.


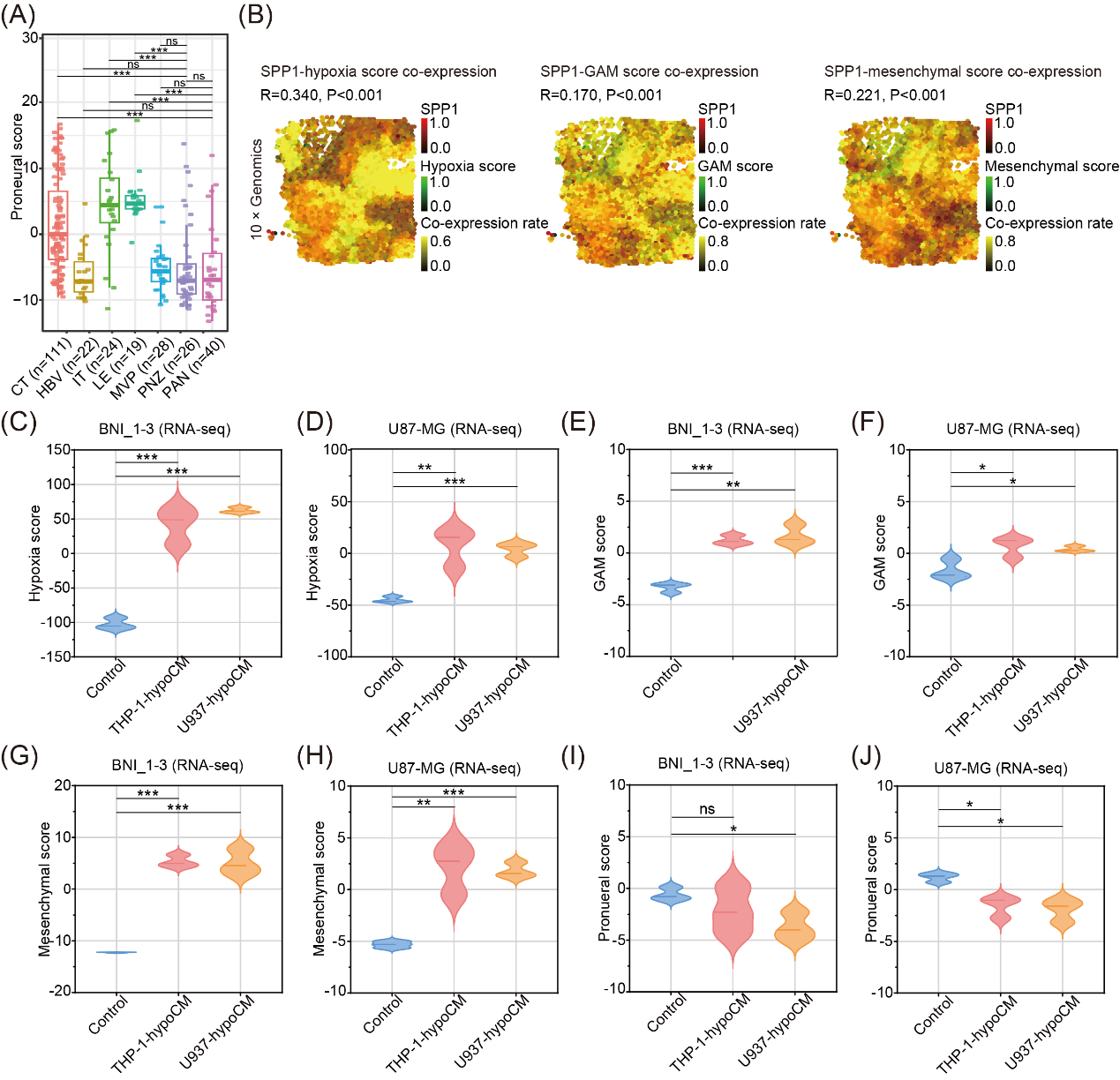


**Supplementary Figure S8. SPP1 is correlated with mesenchymal subtype of gliomas.**

(A) Distribution of proneural score in samples from the IVY Glioblastoma Atlas dataset.

(B) Spatial co-expression rate of SPP1 with hypoxia score, GAM score and mesenchymal score, respectively in 10 × Genomics spatial transcriptomic dataset.

(C-D) Violin plots illustrating hypoxia score generated from RNA-seq data of BNI_1-3 (C) and U87-MG (D) cells cultured with the indicated CM (*n* = 3 per group; control, standard growth medium for macrophages, THP-1-hypoCM, CM obtained from THP-1 cells cultured under hypoxic conditions for 24 h; U937-hypoCM, CM obtained from U937 cells cultured under hypoxic conditions for 24 h).

(E-F) Violin plots illustrating GAM score generated from RNA-seq data of BNI_1-3 (E) and U87-MG (F) cells cultured with the indicated CM (*n* = 3 per group).

(G-H) Violin plots illustrating mesenchymal score generated from RNA-seq data of BNI_1-3 (G) and U87-MG (H) cells cultured with the indicated CM (*n* = 3 per group).

(I-J) Violin plots illustrating proneural score generated from RNA-seq data of BNI_1-3 (I) and U87-MG (J) cells cultured with the indicated CM (*n* = 3 per group).

ns, not significant, **P* < 0.05, ***P* < 0.01, ****P* < 0.001.

Abbreviations: SPP1, secreted phosphoprotein 1; OPN, osteopontin; GAM, glioma-associated macrophage; CM, conditioned media; CT, cellular tumor; HBV, hyperplastic blood vessels; IT, infiltrating tumor; LE, leading edge; MVP, microvascular proliferation; PNZ, perinecrotic zone; PAN, pseudopalisading cells around necrosis.


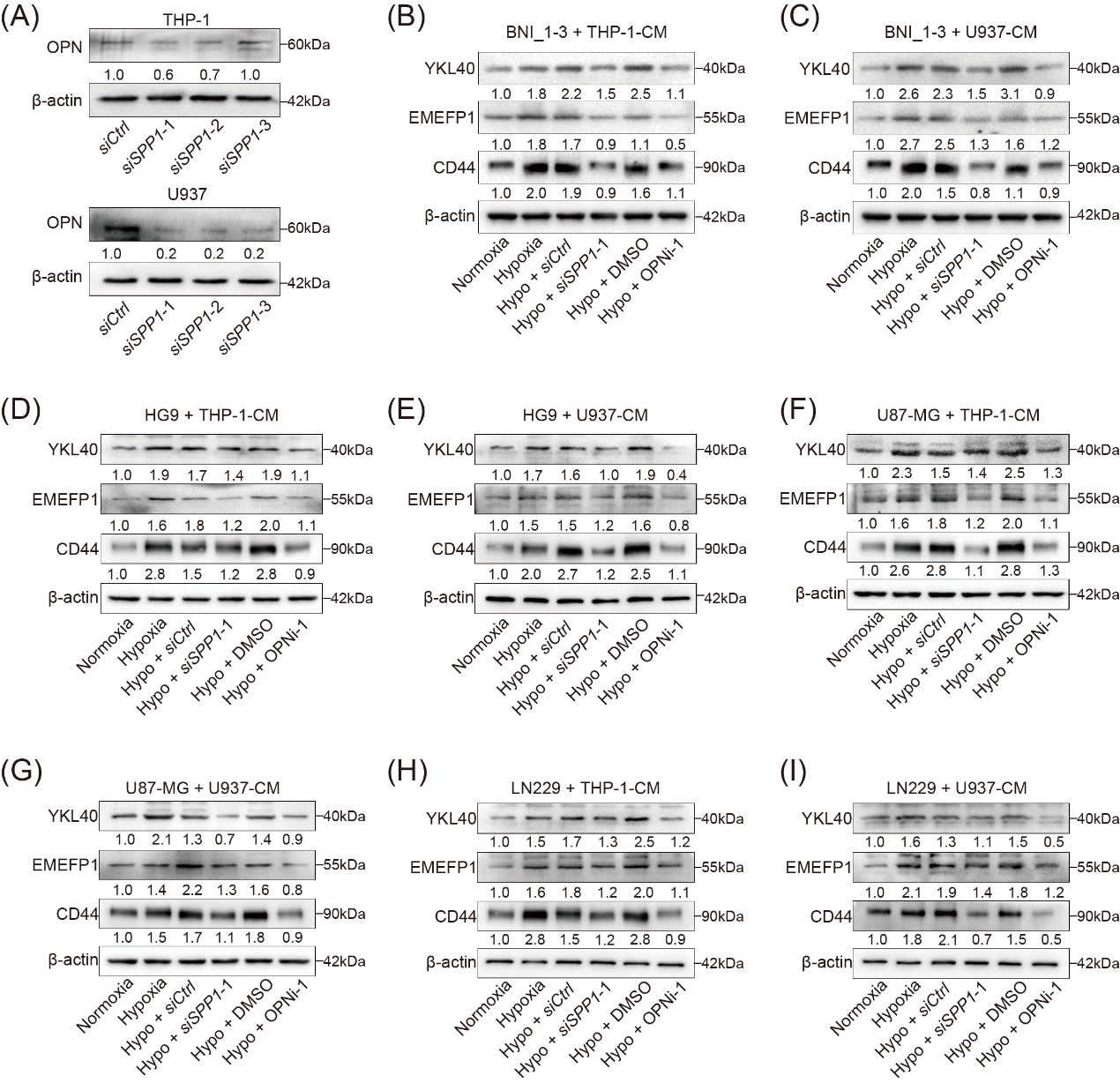


**Supplementary Figure S9. OPN induces mesenchymal transition of glioma cells.**

(A) Western blotting showing knockdown effect of si on OPN expression in THP-1 and U937 cells.

(B-C) Western blotting showing expression levels of representative mesenchymal phenotype related proteins (YKL40, EFEMP1, and CD44) in BNI_1-3 cultured with the CM derived from THP-1 (B) and U937 (C) cells under indicated treatment conditions (normoxia, CM obtained from THP-1 or U937 cells cultured under normoxic condition for 24 h; Hypoxia, CM obtained from THP-1 or U937 cells cultured under hypoxic condition for 24 h; Hypo + siCtrl, CM obtained from siCtrl transfected THP-1 or U937 cells cultured under hypoxic condition for 24 h; Hypo + siSPP1-1, CM obtained from siSPP1-1 transfected THP-1 or U937 cells cultured under hypoxic condition for 24 h; Hypo + DMSO, CM obtained from THP-1 or U937 cells cultured under hypoxic condition for 24 h in the presence of DMSO; Hypo + OPNi-1, CM obtained from THP-1 or U937 cells cultured under hypoxic condition for 24 h in the presence of OPNi-1).

(D-E) Western blotting showing expression levels of representative mesenchymal phenotype related proteins (YKL40, EFEMP1, and CD44) in HG9 cultured with the CM derived from THP-1 (D) and U937 (E) cells under indicated treatment conditions.

(F-G) Western blotting showing expression levels of representative mesenchymal phenotype related proteins (YKL40, EFEMP1, and CD44) in U87-MG cultured with the CM derived from THP-1 (F) and U937 (G) cells under indicated treatment conditions.

(H-I) Western blotting showing expression levels of representative mesenchymal phenotype related proteins (YKL40, EFEMP1, and CD44) in LN229 cultured with the CM derived from THP-1 (H) and U937 (I) cells under indicated treatment conditions.

Abbreviations: SPP1, secreted phosphoprotein 1; OPN, osteopontin; siRNA, small interfering RNA; CM, conditioned media; EFEMP1, EGF Containing Fibulin Extracellular Matrix Protein 1; YKL40, Chitinase-3-Like protein 1; CD44, cluster of differentiation 44; DMSO, Dimethyl sulfoxide; OPNi-1, OPN expression inhibitor 1; kDa, kilodalton.


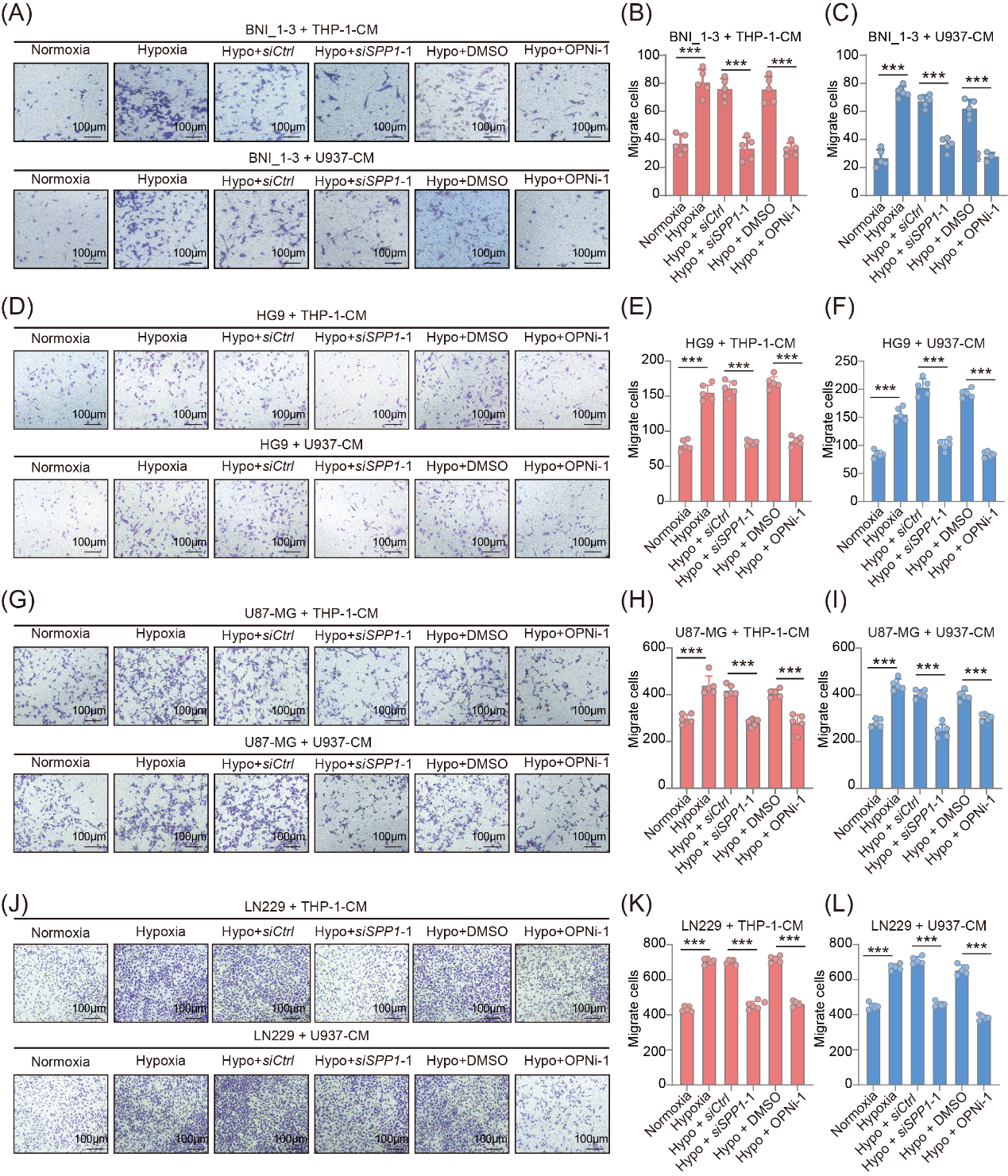


**Supplementary Figure S10. OPN influences migration ability of glioma cells in vitro.**

(A) Transwell assays showing the migration of BNI_1-3 cells cultured with the indicated CM from THP-1 and U937 cells (normoxia, CM obtained from THP-1 or U937 cells cultured under normoxic condition for 24 h; Hypoxia, CM obtained from THP-1 or U937 cells cultured under hypoxic condition for 24 h; Hypo + siCtrl, CM obtained from siCtrl transfected THP-1 or U937 cells cultured under hypoxic condition for 24 h; Hypo + siSPP1-1, CM obtained from siSPP1-1 transfected THP-1 or U937 cells cultured under hypoxic condition for 24 h; Hypo + DMSO, CM obtained from THP-1 or U937 cells cultured under hypoxic condition for 24 h in the presence of DMSO; Hypo + OPNi-1, CM obtained from THP-1 or U937 cells cultured under hypoxic condition for 24 h in the presence of OPNi-1; scale bar = 100 μm).

(B-C) Quantification of migrated BNI_1-3 cells cultured with indicated CM from CM THP-1 (B) and U937 (C) cells (*n* = 5 per group).

(D) Transwell assays showing the migration of HG9 cells cultured with the indicated CM from THP-1 and U937 cells (scale bar = 100 μm).

(E-F) Quantification of migrated HG9 cells cultured with indicated CM from THP-1 (E) and U937 (F) cells (*n* = 5 per group).

(G) Transwell assays showing the migration of U87-MG cells cultured with the indicated CM from THP-1 and U937 cells (scale bar = 100 μm).

(H-I) Quantification of migrated U87-MG cells cultured with indicated CM from THP-1 (H) and U937 (I) cells (*n* = 5 per group).

(J) Transwell assays showing the migration of LN229 cells cultured with the indicated CM from THP-1 and U937 cells (scale bar = 100 μm).

(K-L) Quantification of migrated LN229 cells cultured with indicated CM from THP-1 (K) and U937 (L) cells (*n* = 5 per group).

****P* < 0.001.

Abbreviations: SPP1, secreted phosphoprotein 1; OPN, osteopontin; CM, conditioned media; DMSO, Dimethyl sulfoxide; OPNi-1, OPN expression inhibitor 1.


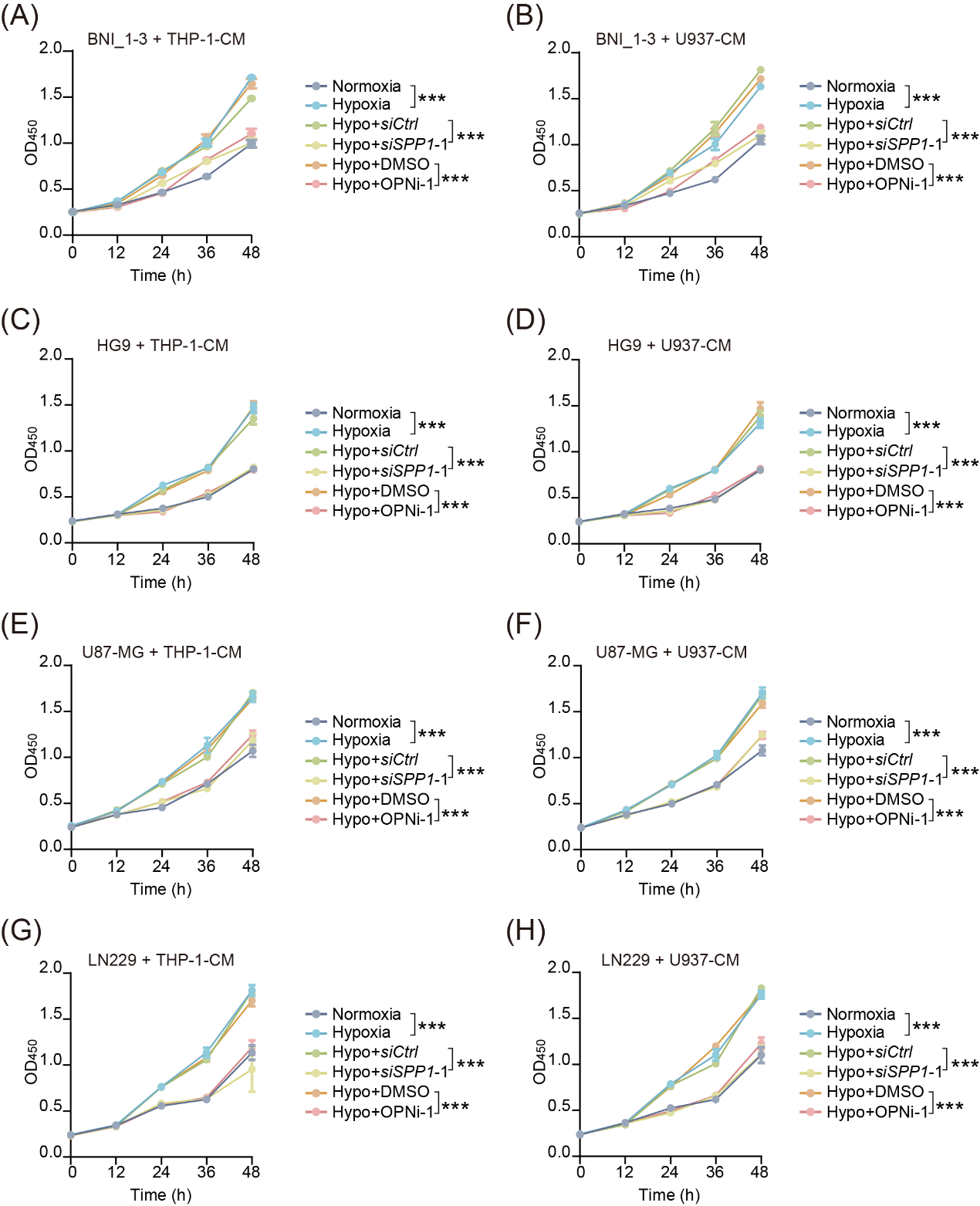


**Supplementary Figure S11. OPN influences proliferation ability of glioma cells in vitro.**

(A-B) CCK-8 assays presenting the proliferation ability of BNI_1-3 cells cultured with indicated CM from THP-1 (A) or U937 (B) cells (*n*=5 per group; normoxia, CM obtained from THP-1 or U937 cells cultured under normoxic condition for 24 h; Hypoxia, CM obtained from THP-1 or U937 cells cultured under hypoxic condition for 24 h; Hypo + siCtrl, CM obtained from siCtrl transfected THP-1 or U937 cells cultured under hypoxic condition for 24 h; Hypo + siSPP1-1, CM obtained from siSPP1-1 transfected THP-1 or U937 cells cultured under hypoxic condition for 24 h; Hypo + DMSO, CM obtained from THP-1 or U937 cells cultured under hypoxic condition for 24 h in the presence of DMSO; Hypo + OPNi-1, CM obtained from THP-1 or U937 cells cultured under hypoxic condition for 24 h in the presence of OPNi-1).

(C-D) CCK-8 assays presenting the proliferation ability of HG9 cells cultured with indicated CM from THP-1 (C) or U937 (D) cells (*n*=5 per group).

(E-F) CCK-8 assays presenting the proliferation ability of U87-MG cells cultured with indicated CM from THP-1 (E) or U937 (F) cells (*n*=5 per group).

(G-H) CCK-8 assays presenting the proliferation ability of LN229 cells cultured with indicated CM from THP-1 (G) or U937 (H) cells (*n*=5 per group).

Data are presented as the mean ± SD, ****P* < 0.001.

Abbreviations: CCK-8, cell counting kit-8; SPP1, secreted phosphoprotein 1; OPN, osteopontin; CM, conditioned media; DMSO, Dimethyl sulfoxide, OPNi-1, OPN expression inhibitor 1.


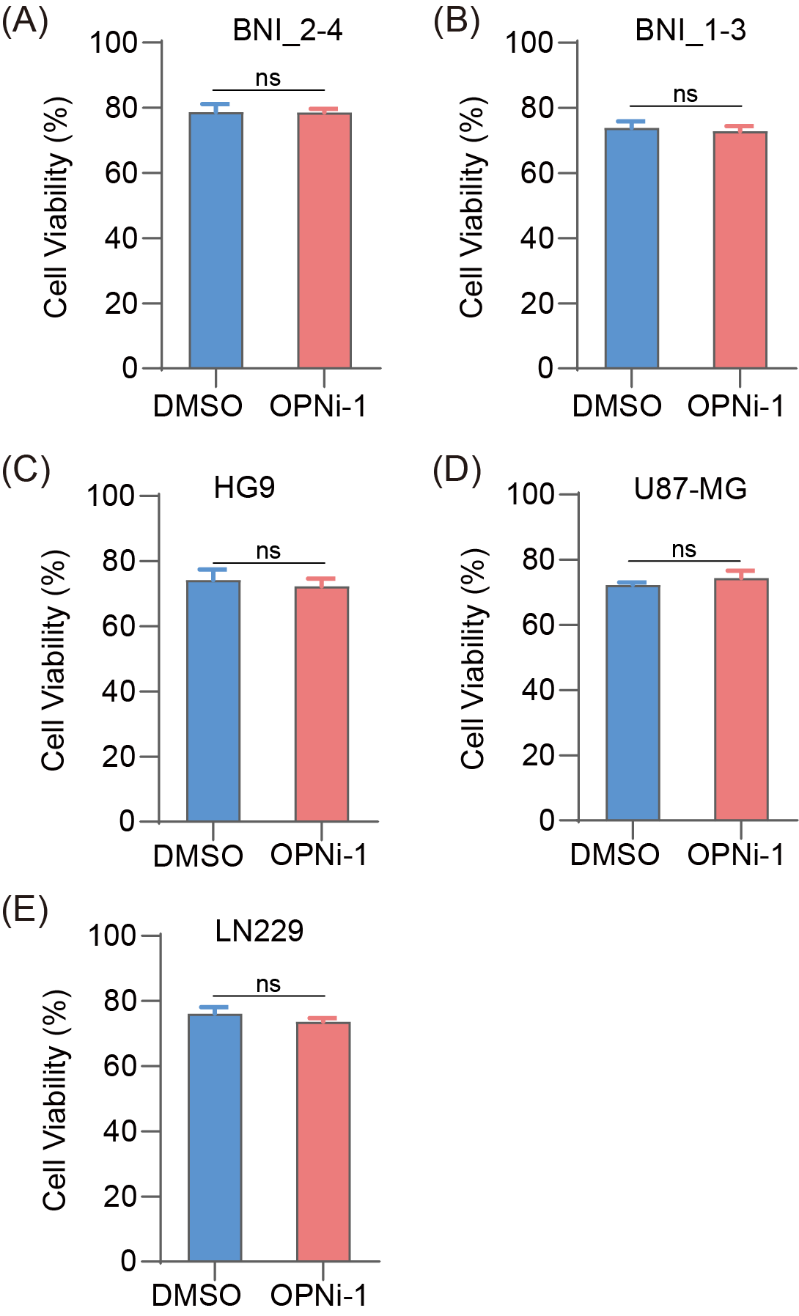


**Supplementary Figure S12. OPN expression inhibitor 1 has no significant effect on glioma cell viability.**

Cell viability of BNI_2-4 (A), BNI_1-3 (B), HG9 (C), U87MG (D), and LN229 (E) treated with DMSO or OPNi-1 (*n* = 3 per group).

Data are presented as the mean ± SD, **P* < 0.05, ***P* < 0.01, ****P* < 0.001.

Abbreviations: DMSO, dimethyl sulfoxide; OPNi-1, OPN expression inhibitor 1.


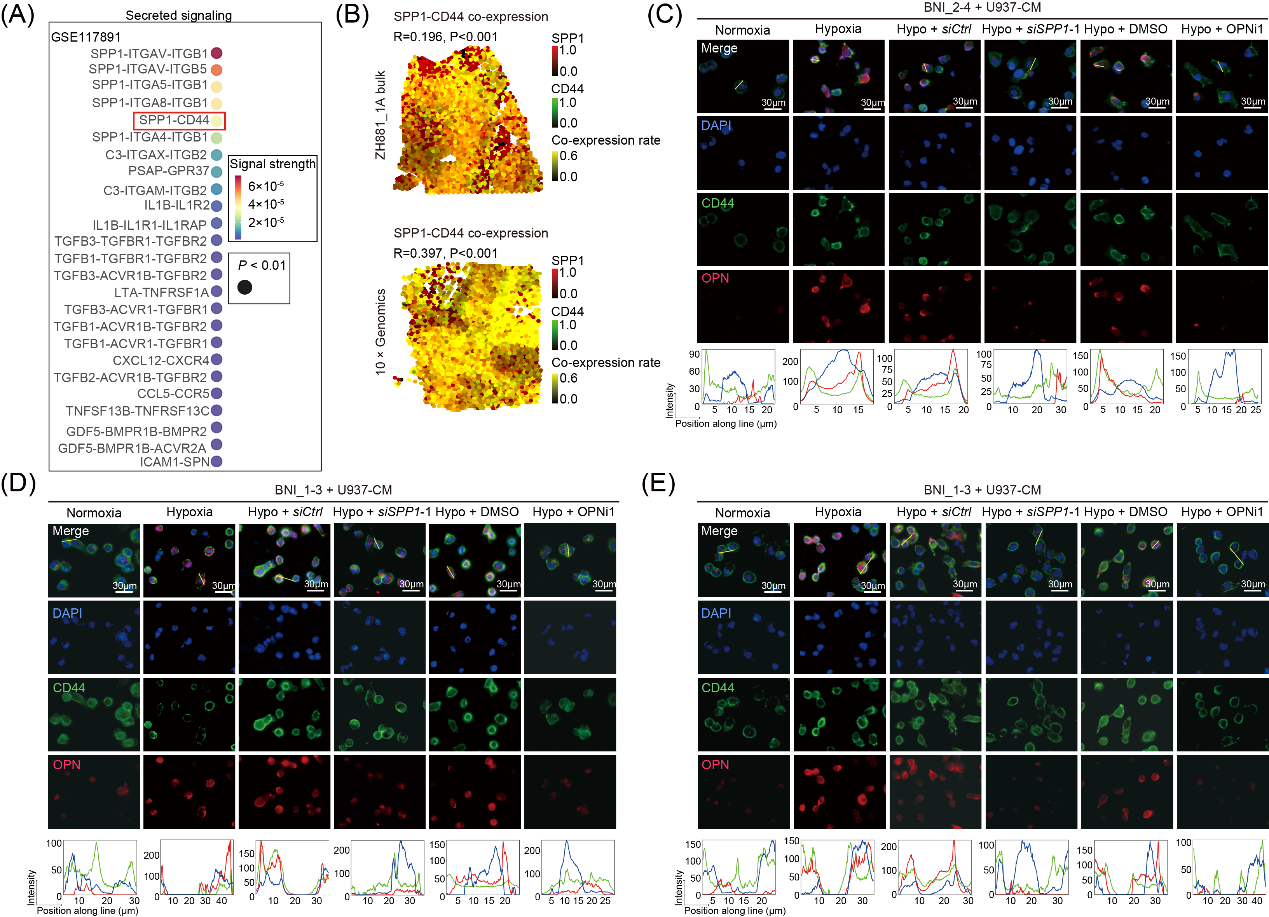


**Supplementary Figure S13. OPN-CD44 mediates intercellular crosstalk between macrophages and glioma cells.**

(A) Bubble plot showing ligand–receptor signaling from macrophage to glioma cells in GSE117891 dataset.

(B) Spatial co-expression rate of SPP1 and CD44 in ZH_881 1A bulk and 10 × Genomics spatial transcriptomic dataset.

(C) IF staining showing the colocalization of OPN (red) and CD44 (green) in BNI_2-4 cells cultured with the indicated CM derived from U937 cells (normoxia, CM obtained from THP-1 or U937 cells cultured under normoxic condition for 24 h; Hypoxia, CM obtained from THP-1 or U937 cells cultured under hypoxic condition for 24 h; Hypo + siCtrl, CM obtained from siCtrl transfected THP-1 or U937 cells cultured under hypoxic condition for 24 h; Hypo + siSPP1-1, CM obtained from siSPP1-1 transfected THP-1 or U937 cells cultured under hypoxic condition for 24 h; Hypo + DMSO, CM obtained from THP-1 or U937 cells cultured under hypoxic condition for 24 h in the presence of DMSO; Hypo + OPNi-1, CM obtained from THP-1 or U937 cells cultured under hypoxic condition for 24 h in the presence of OPNi-1). Nuclei were counterstained with DAPI (blue). The yellow line in the first row indicates the line of interest used for intensity analysis. Line intensity profiles below display the fluorescence signal distribution along the marked line.

(D-E) IF staining showing the colocalization of OPN (red) and CD44 (green) in BNI_1-3 cells cultured with the indicated CM derived from THP-1 (D) and U937 (E) cells. Nuclei were counterstained with DAPI (blue). The yellow line in the first row indicates the line of interest used for intensity analysis. Line intensity profiles below display the fluorescence signal distribution along the marked line.

Abbreviations: SPP1, secreted phosphoprotein 1; CD44, cluster of differentiation 44; OPN, osteopontin; CM, conditioned media; DMSO, Dimethyl sulfoxide; OPNi-1, OPN expression inhibitor 1.


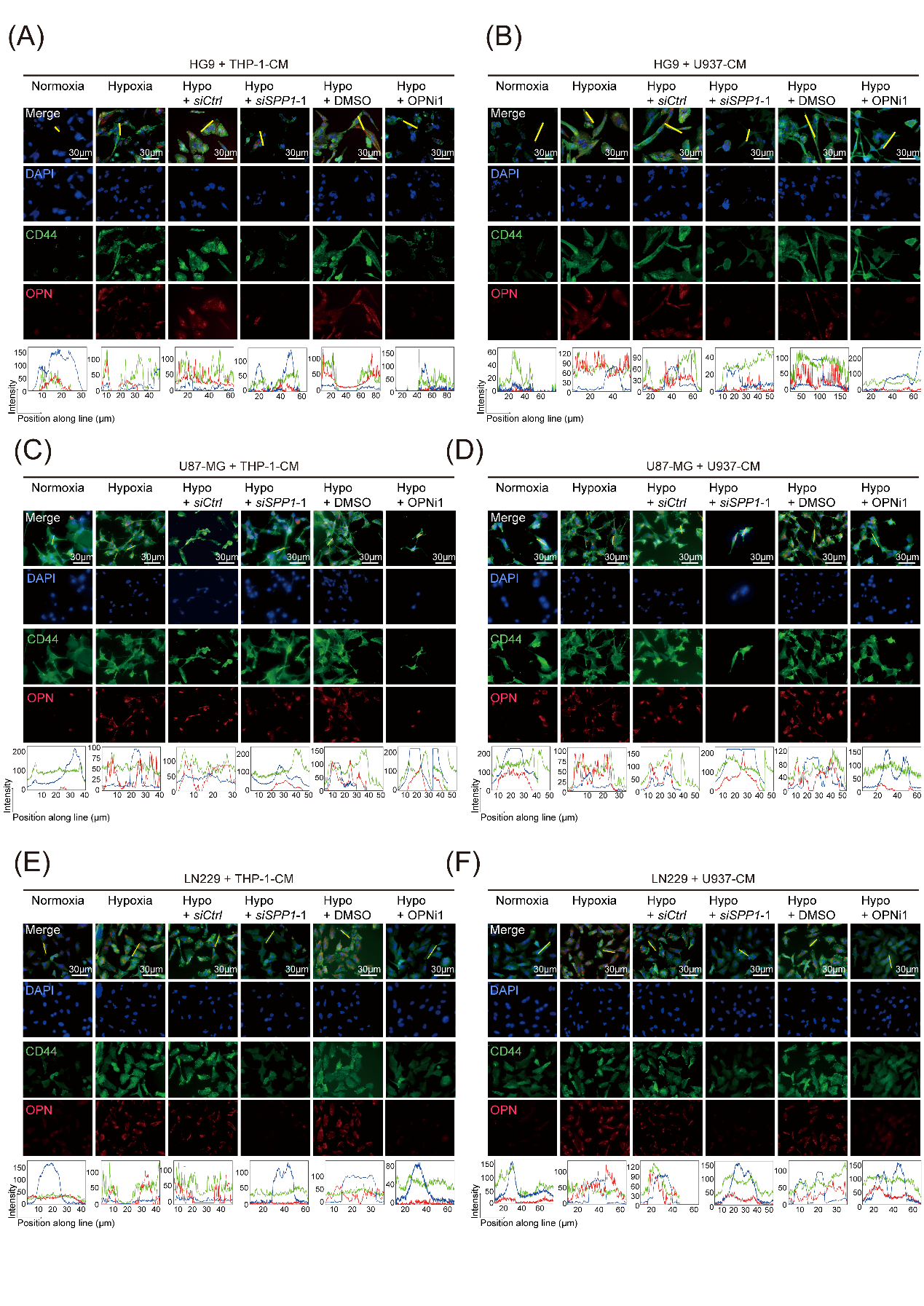


**Supplementary Figure S14. OPN binds with CD44 in vitro.**

(A-B) IF staining showing the colocalization of OPN (red) and CD44 (green) in HG9 cells cultured with the indicated CM derived from THP-1 (A) and U937 (B) cells (normoxia, CM obtained from THP-1 or U937 cells cultured under normoxic condition for 24 h; Hypoxia, CM obtained from THP-1 or U937 cells cultured under hypoxic condition for 24 h; Hypo + siCtrl, CM obtained from siCtrl transfected THP-1 or U937 cells cultured under hypoxic condition for 24 h; Hypo + siSPP1-1, CM obtained from siSPP1-1 transfected THP-1 or U937 cells cultured under hypoxic condition for 24 h; Hypo + DMSO, CM obtained from THP-1 or U937 cells cultured under hypoxic condition for 24 h in the presence of DMSO; Hypo + OPNi-1, CM obtained from THP-1 or U937 cells cultured under hypoxic condition for 24 h in the presence of OPNi-1). Nuclei were counterstained with DAPI (blue). The yellow line in the first row indicates the line of interest used for intensity analysis. Line intensity profiles below display the fluorescence signal distribution along the marked line.

(C-D) IF staining showing the colocalization of OPN (red) and CD44 (green) in U87-MG cells cultured with the indicated CM derived from THP-1 (C) and U937 (D) cells. Nuclei were counterstained with DAPI (blue). The yellow line in the first row indicates the line of interest used for intensity analysis. Line intensity profiles below display the fluorescence signal distribution along the marked line.

(E-F) IF staining showing the colocalization of OPN (red) and CD44 (green) in LN229 cells cultured with the indicated CM derived from THP-1 (C) and U937 (D) cells. Nuclei were counterstained with DAPI (blue). The yellow line in the first row indicates the line of interest used for intensity analysis. Line intensity profiles below display the fluorescence signal distribution along the marked line.

Abbreviations: OPN, osteopontin; CM, conditioned media DMSO, Dimethyl sulfoxide; OPNi-1, OPN expression inhibitor 1.


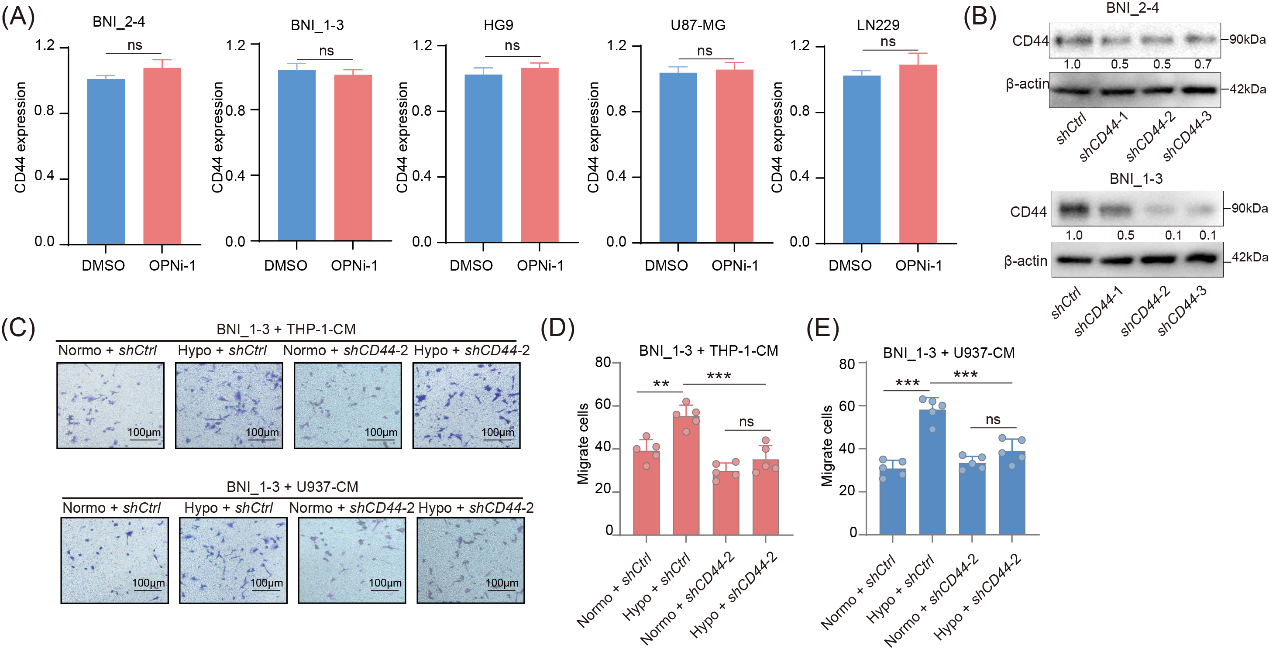


**Supplementary Figure S15. CD44 knockdown reduces the migrating ability of glioma cells.**

(A) QPCR analysis of CD44 expression in glioma cells treated with DMSO or OPNi-1 (*n* = 3 per group).

(B) Western blotting showing knockdown effect of shCD44 on CD44 expression in BNI_2-4 and BNI_1-3 cells.

(C) Transwell assays showing the migration of BNI_1-3 cells cultured under the indicated conditions with CM of THP-1 and U937 cells (normo + shCtrl, glioma cells which were transinfected with negative control lentivirus cultured in normoxic CM; hypo + shCtrl, glioma cells which were transinfected with negative control lentivirus cultured in hypxic CM; normo + shCD44-2, glioma cells which were transinfected with shCD44-2 lentivirus cultured in normoxic CM; hypo + shCD44-2, glioma cells which were transinfected with shCD44-2 lentivirus cultured in hypxic CM; scale bar = 100 μm).

(D-E) Quantification of migrated BNI_1-3 cells cultured under the indicated conditions with CM of THP-1 (D) and U937 (E) cells (*n* = 5 per group).

Data are presented as the mean ± SD, ns, not significant, ***P* < 0.01, ****P* < 0.001.

Abbreviations: DMSO, Dimethyl sulfoxide; OPNi-1, OPN expression inhibitor 1; CD44, cluster of differentiation 44; qPCR, quantitative real-time PCR; CM, conditioned medium; SD, standard deviation; kDa, kilodalton.


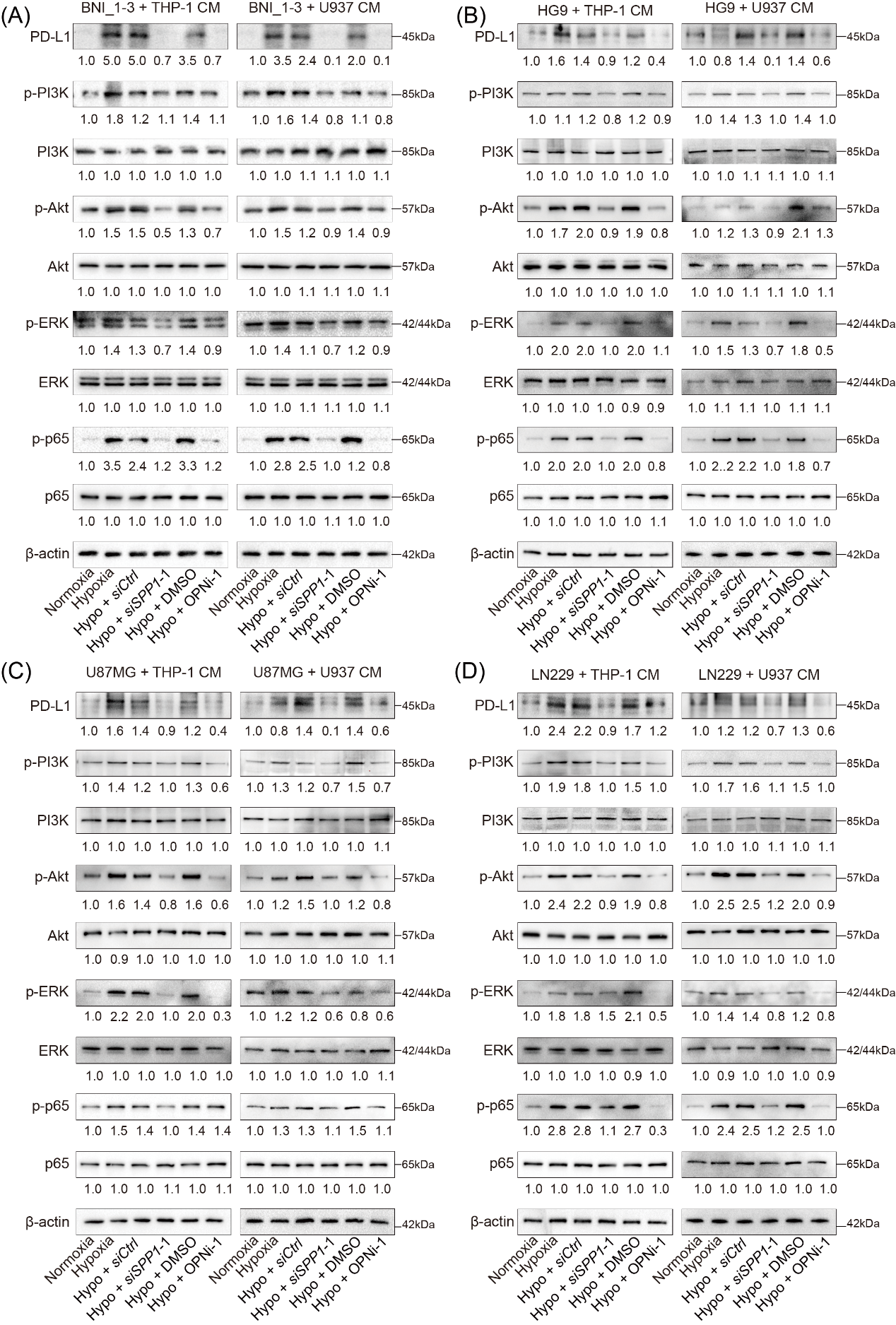


**Supplementary Figure S16. OPN activates NF-κB signaling pathway in glioma cells.**

Western blotting showing expression levels of PD-L1 and NF-κB signaling pathway related proteins in BNI_1-3 (A), HG9 (B), U87-MG (C), and LN229 (D) cells cultured with indicated CM derived from THP-1 and U937 cells (normoxia, CM obtained from THP-1 or U937 cells cultured under normoxic condition for 24 h; Hypoxia, CM obtained from THP-1 or U937 cells cultured under hypoxic condition for 24 h; Hypo + siCtrl, CM obtained from siCtrl transfected THP-1 or U937 cells cultured under hypoxic condition for 24 h; Hypo + siSPP1-1, CM obtained from siSPP1-1 transfected THP-1 or U937 cells cultured under hypoxic condition for 24 h; Hypo + DMSO, CM obtained from THP-1 or U937 cells cultured under hypoxic condition for 24 h in the presence of DMSO; Hypo + OPNi-1, CM obtained from THP-1 or U937 cells cultured under hypoxic condition for 24 h in the presence of OPNi-1).

Abbreviations: PD-L1, programmed cell death ligand 1; PI3K, phosphatidylinositol kinase 3; p-PI3K, phosphorylated phosphatidylinositol kinase 3; Akt, protein kinase B; p-AKT, phosphorylated protein kinase B; ERK1/2, extracellular signal-regulated protein kinases 1/2; p-ERK1/2, phosphorylated extracellular signal-regulated protein kinases 1/2; NF-κB, nuclear factor kappa-B; p-p65, phosphorylated nuclear factor kappa-B; CM, conditioned media; DMSO, dimethyl sulfoxide; OPNi-1, OPN expression inhibitor 1; kDa, kilodaltons.


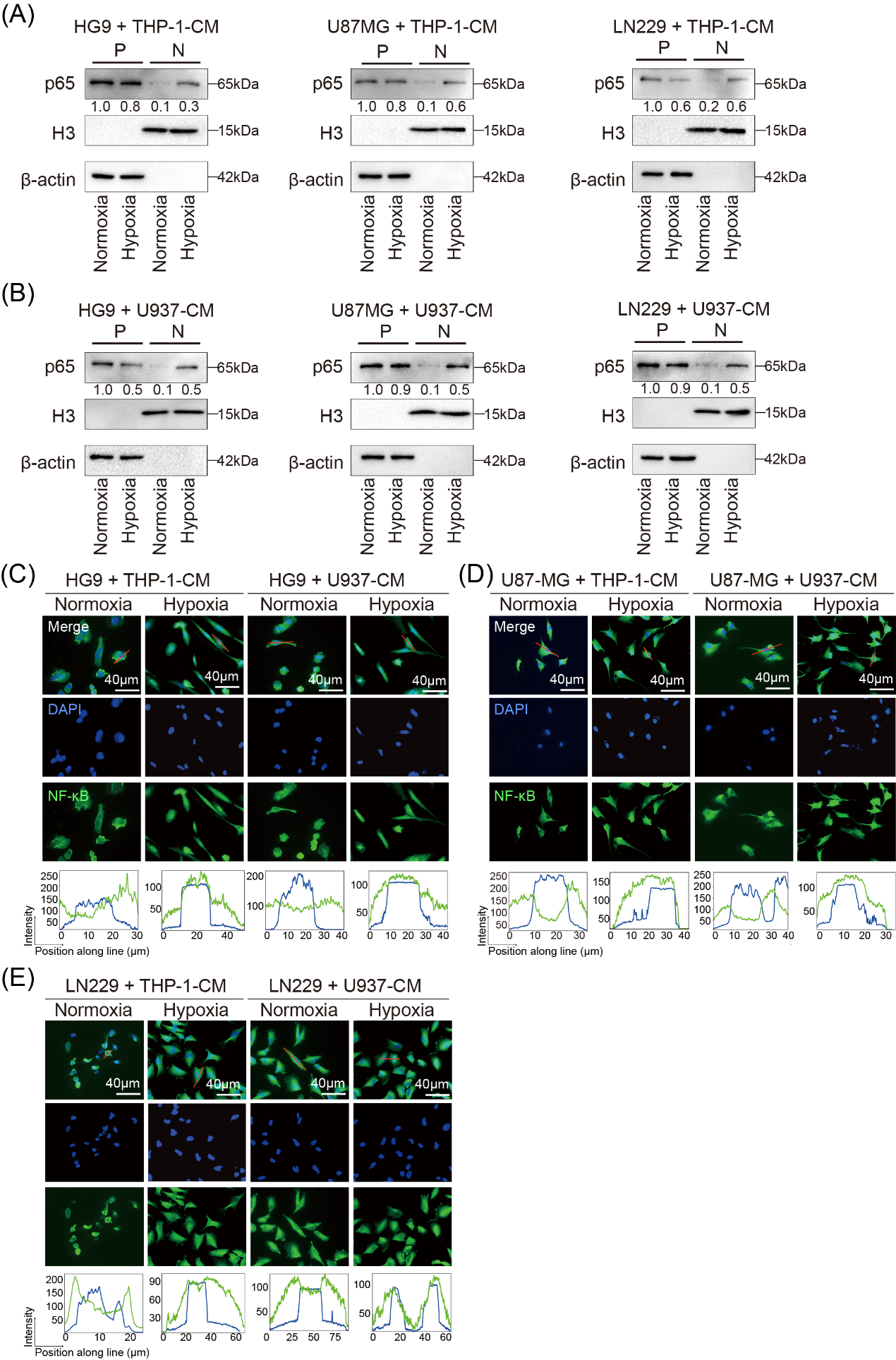


**Supplementary Figure S17. Hypoxic CM induces NF-κB activation and nuclear translocation in glioma cells.**

(A) Western blot assays showing NF-κB (p65 subunit) expression in cytoplasmic and nuclear fractions of HG9, U87-MG, and LN229 cells cultured with normoxic and hypoxic CM derived from THP-1 cells. β-actin and H3 were used as cytoplasmic and nuclear loading controls, respectively (P, cytoplasm; N, nuclei).

(B) Western blotting showing NF-κB (p65 subunit) expression in cytoplasmic and nuclear fractions of HG9, U87MG, and LN229 cells cultured with normoxic and hypoxic CM derived from U937 cells. β-actin and H3 were used as cytoplasmic and nuclear loading controls, respectively (P, cytoplasm; N, nuclei).

(C-E) IF staining showing the localization of NF-κB (p65 subunit, green) in HG9 (C), U87-MG (D), and LN229 (E) cells cultured with normoxic and hypoxic CM derived from THP-1 and U937 cells. Nuclei were counterstained with DAPI (blue). The red line in the first row indicates the line of interest used for intensity analysis. The line intensity profiles below display the fluorescence signal distribution along the marked line.

Abbreviations: NF-κB, nuclear factor kappa-B; CM, conditioned media; kDa, kilodalton.


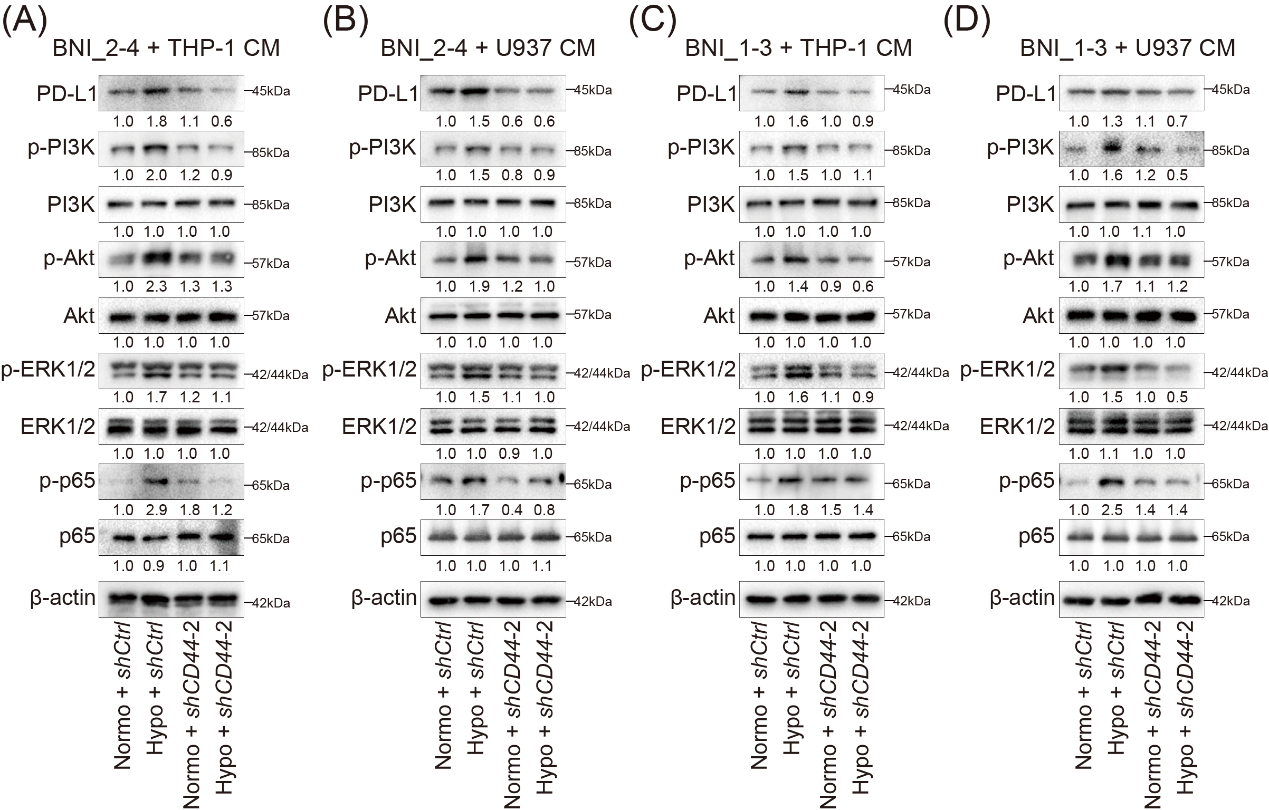


**Supplementary Figure S18. CD44 knockdown reduces activation of the NF-κB signaling pathway in glioma cells cultured with OPN-containing conditioned medium.**

(A-B) Western blotting showing expression levels of PD-L1 and NF-κB signaling pathway related proteins in BNI_2-4 cells cultured with THP-1 (A) and U937 (B) CM under the indicated conditions (normo + shCtrl, glioma cells which were transinfected with negative control lentivirus cultured in normoxic CM; hypo + shCtrl, glioma cells which were transinfected with negative control lentivirus cultured in hypxic CM; normo + shCD44-2, glioma cells which were transinfected with shCD44-2 lentivirus cultured in normoxic CM; hypo + shCD44-2, glioma cells which were transinfected with shCD44-2 lentivirus cultured in hypxic CM).

(C-D) Western blotting showing expression levels of PD-L1 and NF-κB signaling pathway related proteins in BNI_1-3 cells cultured with THP-1 (C) and U937 (D) CM under the indicated conditions.

Abbreviations: PD-L1, programmed cell death ligand 1; PI3K, phosphatidylinositol kinase 3; p-PI3K, phosphorylated phosphatidylinositol kinase 3; Akt, protein kinase B; p-AKT, phosphorylated protein kinase B; ERK1/2, extracellular regulated protein kinases 1/2; p-ERK1/2, phosphorylated extracellular regulated protein kinases 1/2; NF-κB, nuclear factor kappa-B; p-p65, phosphorylated nuclear factor kappa-B; CM, conditioned media; kDa, kilodalton.


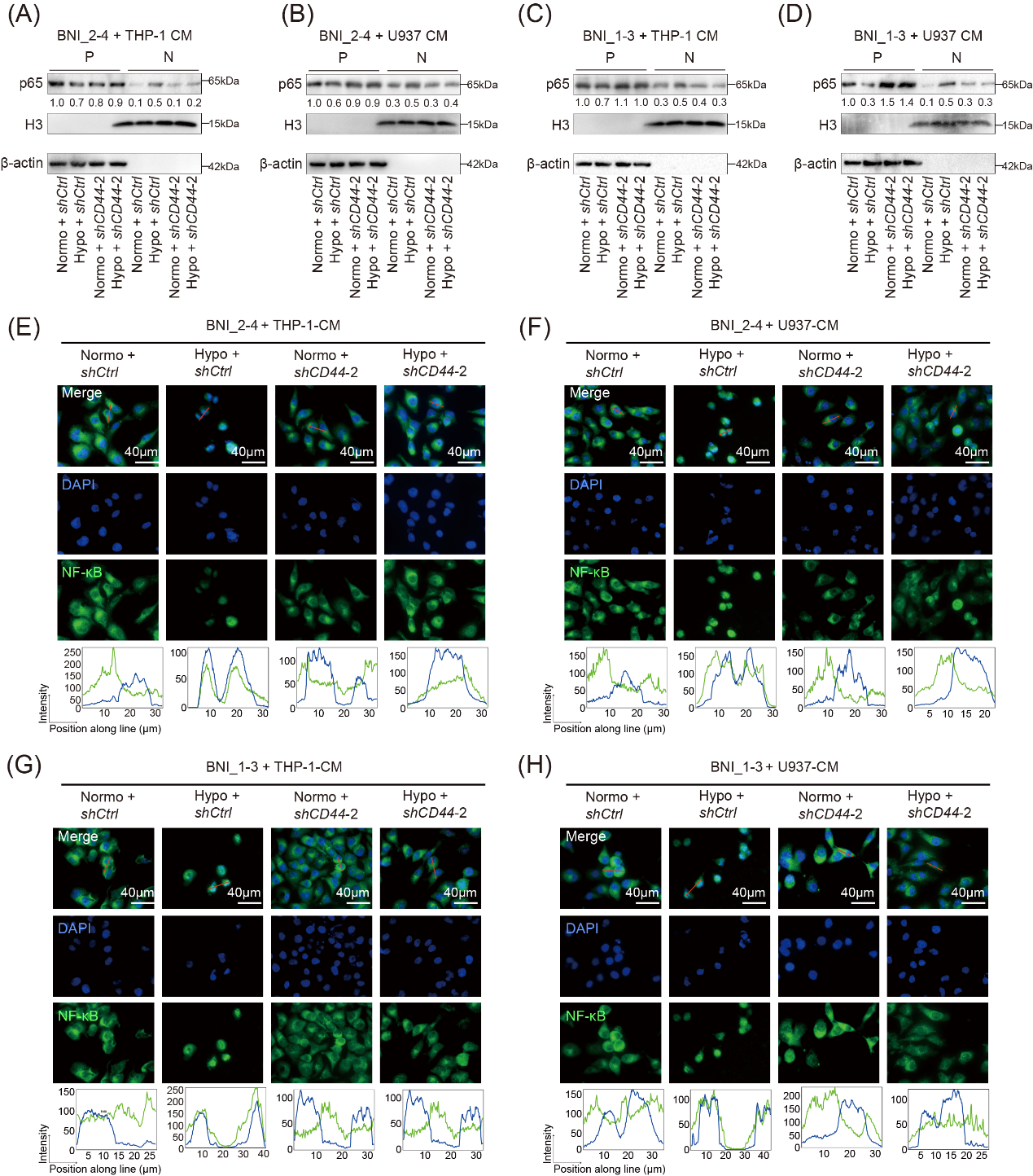


**Supplementary Figure S19. CD44 knockdown reduces NF-κB translocation in glioma cells cultured with OPN-containing conditioned medium.**

(A-B) Western blotting showing NF-κB (p65 subunit) expression in cytoplasmic and nuclear fractions of BNI_2-4 cells cultured with CM derived from THP-1 (A) and U937 (B) cells under indicated conditions (normo + shCtrl, glioma cells which were transinfected with negative control lentivirus cultured in normoxic CM; hypo + shCtrl, glioma cells which were transinfected with negative control lentivirus cultured in hypxic CM; normo + shCD44-2, glioma cells which were transinfected with shCD44-2 lentivirus cultured in normoxic CM; hypo + shCD44-2, glioma cells which were transinfected with shCD44-2 lentivirus cultured in hypxic CM). β-actin and H3 were used as cytoplasmic and nuclear loading controls, respectively (P, cytoplasm; N, nuclei).

(C-D) Western blotting showing NF-κB (p65 subunit) expression in cytoplasmic and nuclear fractions of BNI_1-3 cells cultured with CM derived from THP-1 (C) and U937 (D) cells under indicated conditions. β-actin and H3 were used as cytoplasmic and nuclear loading controls, respectively (P, cytoplasm; N, nuclei).

(E-F) IF staining showing the localization of NF-κB (p65 subunit, green) in BNI_2-4 cells cultured with normoxic and hypoxic CM derived from THP-1 (E) and U937 cells (F) under indicated conditions. Nuclei were counterstained with DAPI (blue). The red line in the first row indicates the line of interest used for intensity analysis. The line intensity profiles below display the fluorescence signal distribution along the marked line.

(G-H) IF staining showing the localization of NF-κB (p65 subunit, green) in BNI_1-3 cells cultured with normoxic and hypoxic CM derived from THP-1 (G) and U937 cells (H) under indicated conditions. Nuclei were counterstained with DAPI (blue). The red line in the first row indicates the line of interest used for intensity analysis. The line intensity profiles below display the fluorescence signal distribution along the marked line.

Abbreviations: NF-κB, nuclear factor kappa-B; CM, conditioned media; kDa, kilodalton.


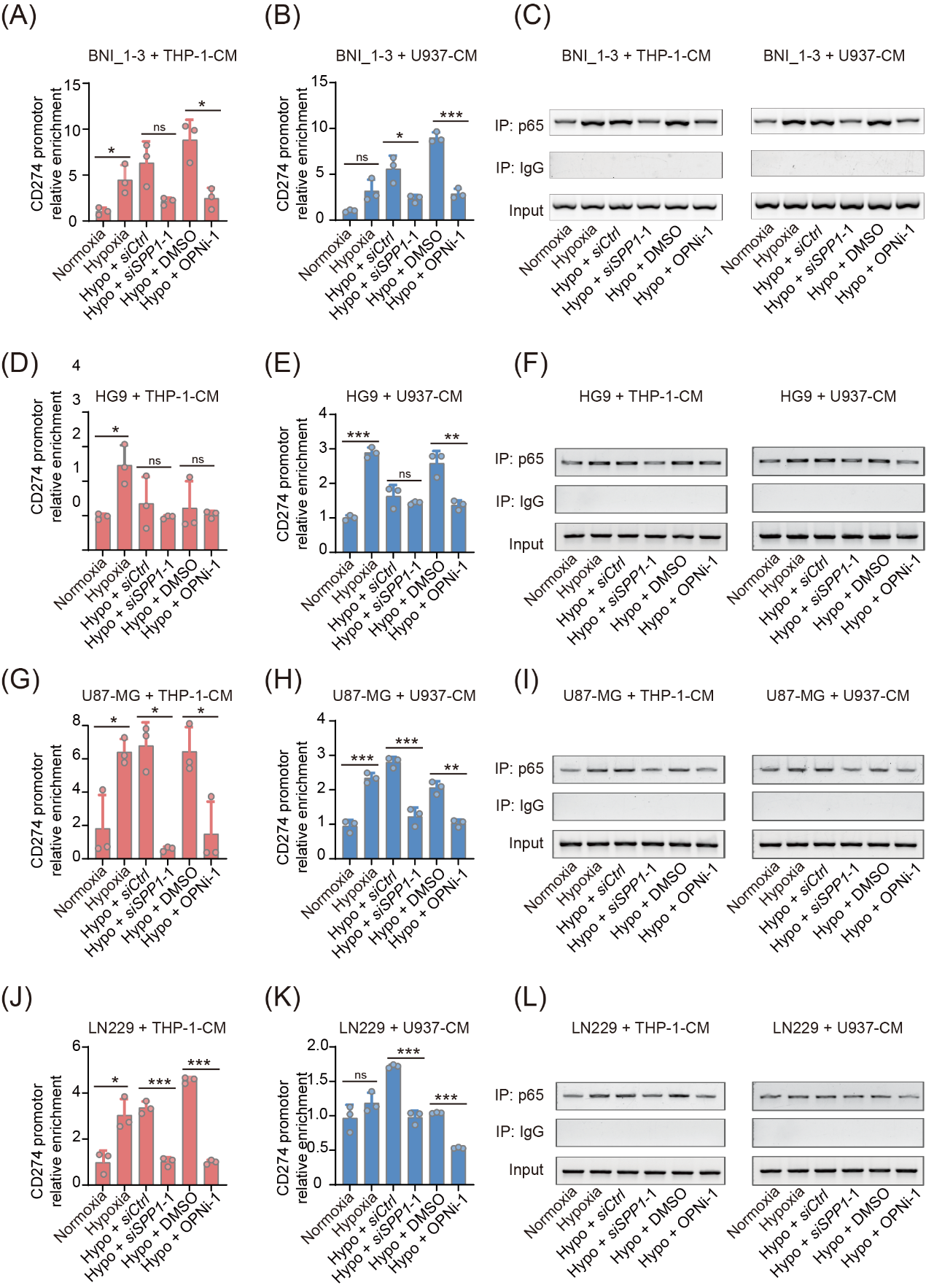


**Supplementary Figure S20. ChIP analysis confirms p65 binding to the CD274 promoter.**

(A-B) QPCR analysis of NF-κB (p65 subunit) enrichment at CD274 promoter area in BNI_1-3 cells cultured with indicated CM derived from THP-1 (A) and U937 (B) cells (*n* = 3 per group).

(C) Agarose gel electrophoresis showing ChIP-PCR products (CD274 promoter sequence) from BNI_1-3 cells cultured with indicated CM derived from THP-1 and U937 cells. Chromatin was immunoprecipitated using anti-NF-κB (p65 subunit) and IgG control antibodies.

(D-E) QPCR analysis of NF-κB (p65 subunit) enrichment at CD274 promoter area in HG9 cells cultured with indicated CM derived from THP-1 (D) and U937 (E) cells (*n* = 3 per group).

(F) Agarose gel electrophoresis showing ChIP-PCR products (CD274 promoter sequence) from HG9 cells cultured with indicated CM derived from THP-1 and U937 cells. Chromatin was immunoprecipitated using anti-NF-κB (p65 subunit) and IgG control antibodies.

(G-H) QPCR analysis of NF-κB (p65 subunit) enrichment at CD274 promoter area in U87-MG cells cultured with the indicated CM derived from THP-1(G) and U937 (H) cells (*n* = 3 per group).

(I) Agarose gel electrophoresis showing ChIP-PCR products (CD274 promoter sequence) from U87-MG cells cultured with indicated CM derived from THP-1 and U937 cells. Chromatin was immunoprecipitated using anti-NF-κB (p65 subunit) and IgG control antibodies.

(J-K) QPCR analysis of NF-κB (p65 subunit) enrichment at CD274 promoter area in LN229 cells cultured with indicated CM derived from THP-1 (J) and U937 (K) cells (*n* = 3 per group).

(L) Agarose gel electrophoresis showing ChIP-PCR products (CD274 promoter sequence) from LN229 cells cultured with indicated CM derived from THP-1 and U937 cells. Chromatin was immunoprecipitated using anti-NF-κB (p65 subunit) and IgG control antibodies.

Data are presented as the mean ± SD, ns, not significant **P* < 0.05; ***P* < 0.01; ****P* < 0.001.

Abbreviations: NF-κB, nuclear factor kappa-B; CM, conditioned media, ChIP, chromatin immunoprecipitation, qPCR, quantitative real-time PCR.


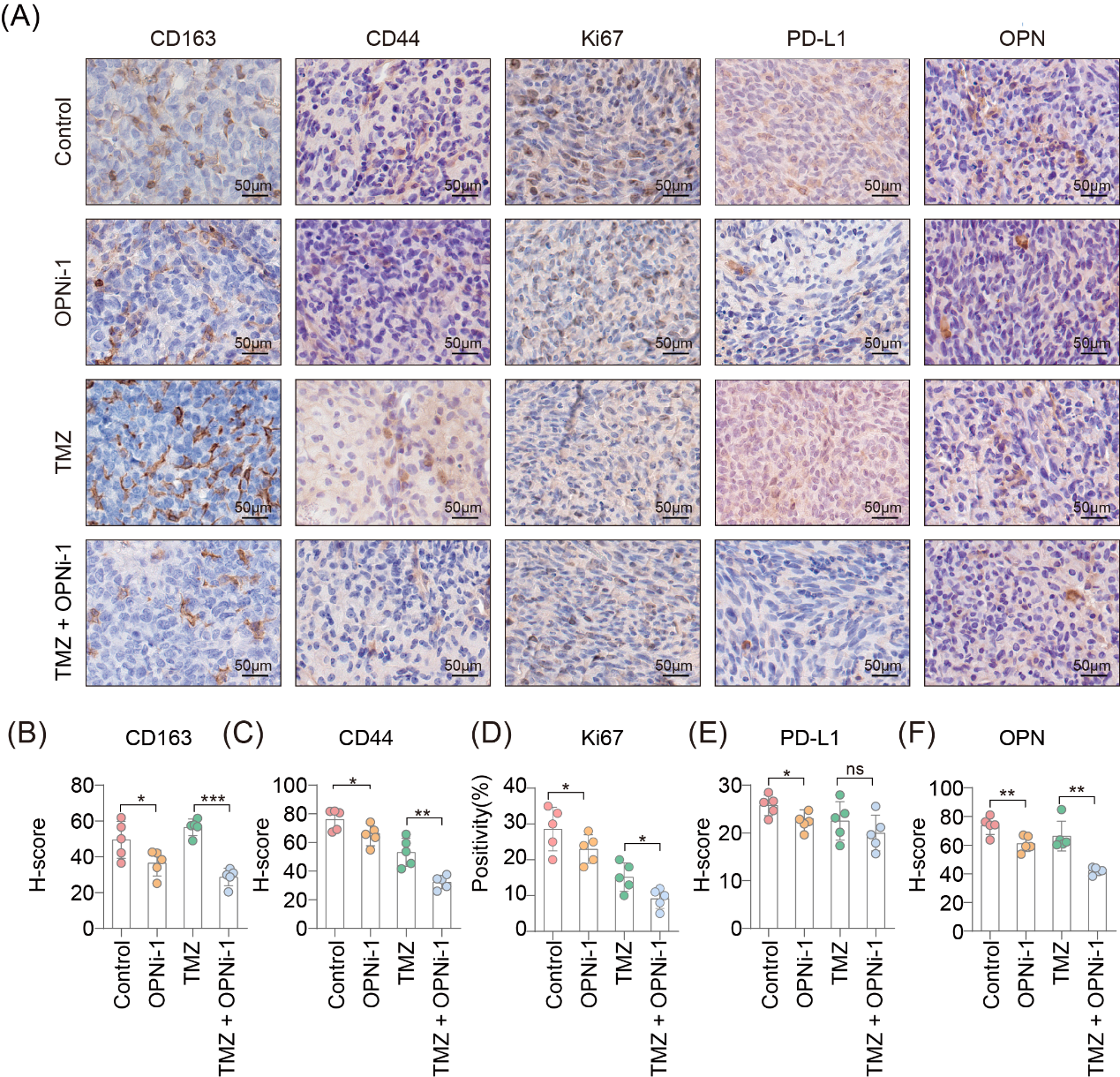


**Supplementary Figure S21. OPNi-1 facilitates glioma chemotherapy by reducing OPN expression in vivo.**

(A) Representative images of IHC staining for CD163, CD44, Ki67, PD-L1, and OPN in glioma tissues from C57BL/6J mice in the indicated treatment groups (control, treatment with PBS; OPNi-1, treatment with OPNi-1 alone; TMZ, treatment with TMZ alone; TMZ + OPNi-1, combined treatment with TMZ and OPNi-1).

(B) Quantification of IHC staining for CD163 in glioma tissues from mice in the indicated treatment groups was performed by calculating H-scores (*n* = 5 per group).

(C) Quantification of IHC staining for CD44 in glioma tissues from mice in the indicated treatment groups by calculating H-scores (*n* = 5 per group).

(D) Quantification of IHC staining for Ki67 in glioma tissues from mice in the indicated treatment groups by estimating positive staining (*n* = 5 per group).

(E) Quantification of IHC staining for PD-L1 in glioma tissues from mice in the indicated treatment groups by calculating H-scores (*n* = 5 per group).

(F) Quantification of IHC staining for OPN in glioma tissues of mice from different groups by calculating H-scores (*n* = 5 per group).

The data are presented as the means ± SDs, ns, not significant, **P* < 0.05, ***P* < 0.01, ****P* < 0.001.

Abbreviations: IHC, immunohistochemistry; PBS, phosphate buffered saline; TMZ, temozolomide; PD-L1, programmed cell death ligand 1; CD44, CD44, cluster of differentiation 44; OPN, osteopontin; OPNi-1, OPN expression inhibitor 1; CD163, cluster of differentiation 163; SD, standard deviation.


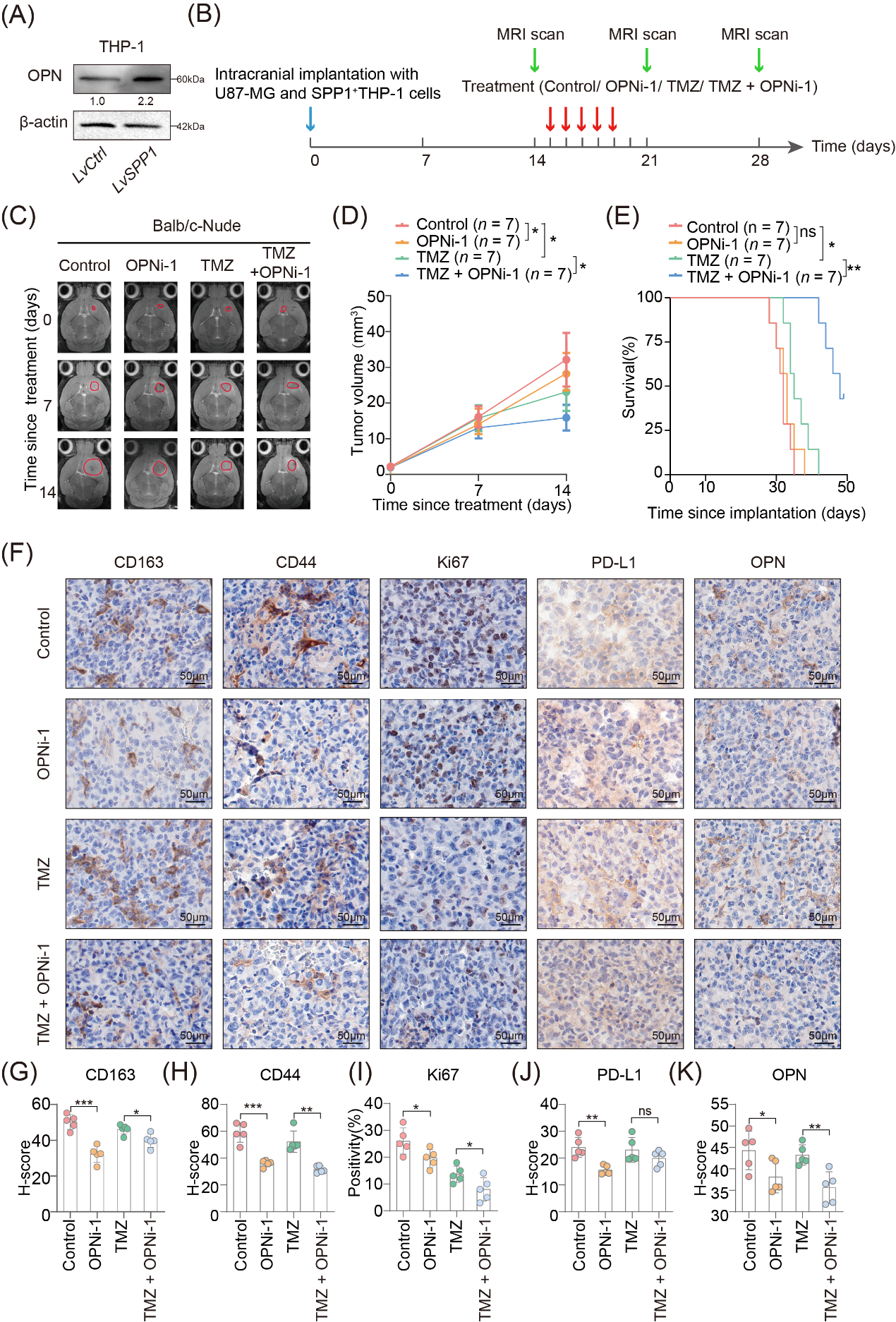


**Supplementary Figure S22. Targeting OPN enhances the therapeutic efficacy of TMZ in Balb/c-nude in vivo glioma model.**

(A) Western blotting confirming the overexpression of OPN in THP-1 cells treated with SPP1 overexpression lentivirus.

(B) Schematic illustration of the in vivo experimental design in Balb/c-nude mice. The blue arrow represents intracranial implantation; the red arrows represent treatments (control, treatment with PBS; OPNi-1, treatment with OPNi-1 alone; TMZ, treatment with TMZ alone; TMZ + OPNi-1, combined treatment with TMZ and OPNi-1); the green arrows represent MRI scans.

(C) Representative MR images showing the intracranial tumor burden in Balb/c-nude mice from the indicated treatment groups.

(D) Quantification of the tumor volume in Balb/c-nude mice from the indicated groups on day, day 7, and day 14 since initial treatment (*n* = 7 mice per group).

(E) Kaplan–Meier survival curves of glioma-bearing mice receiving the indicated treatments (*n* = 7 mice per group).

(F) Representative images IHC staining for CD163, CD44, Ki67, PD-L1, and OPN in tumors of Balb/c-nude mice from indicated treatment groups.

(G) Quantification of IHC staining for CD163 in glioma tissues of mice from indicated treatment groups using H-score calculating (*n* = 5 per group).

(H) Quantification of IHC staining for CD44 in glioma tissues of mice from indicated treatment groups using H-score calculating (*n* = 5 per group).

(I) Quantification of IHC staining for Ki67 in glioma tissues of mice from indicated treatment groups by positivity estimating (*n* = 5 per group).

(J) Quantification of IHC staining for PD-L1 in glioma tissues of mice from indicated treatment groups using H-score calculating (*n* = 5 per group).

(K) Quantification of IHC staining for OPN in glioma tissues of mice from indicated treatment groups using H-score calculating (*n* = 5 per group).

Data are presented as the mean ± SD, ns, not significant, **P* < 0.05, ***P* < 0.01, ****P* < 0.001.

Abbreviations: PBS, phosphate buffered saline; TMZ, temozolomide; PD-L1, programmed cell death ligand 1; CD44, cluster of differentiation 44; OPN, osteopontin; OPNi-1, OPN expression inhibitor 1; CD163, cluster of differentiation 163; SD, standard deviation.


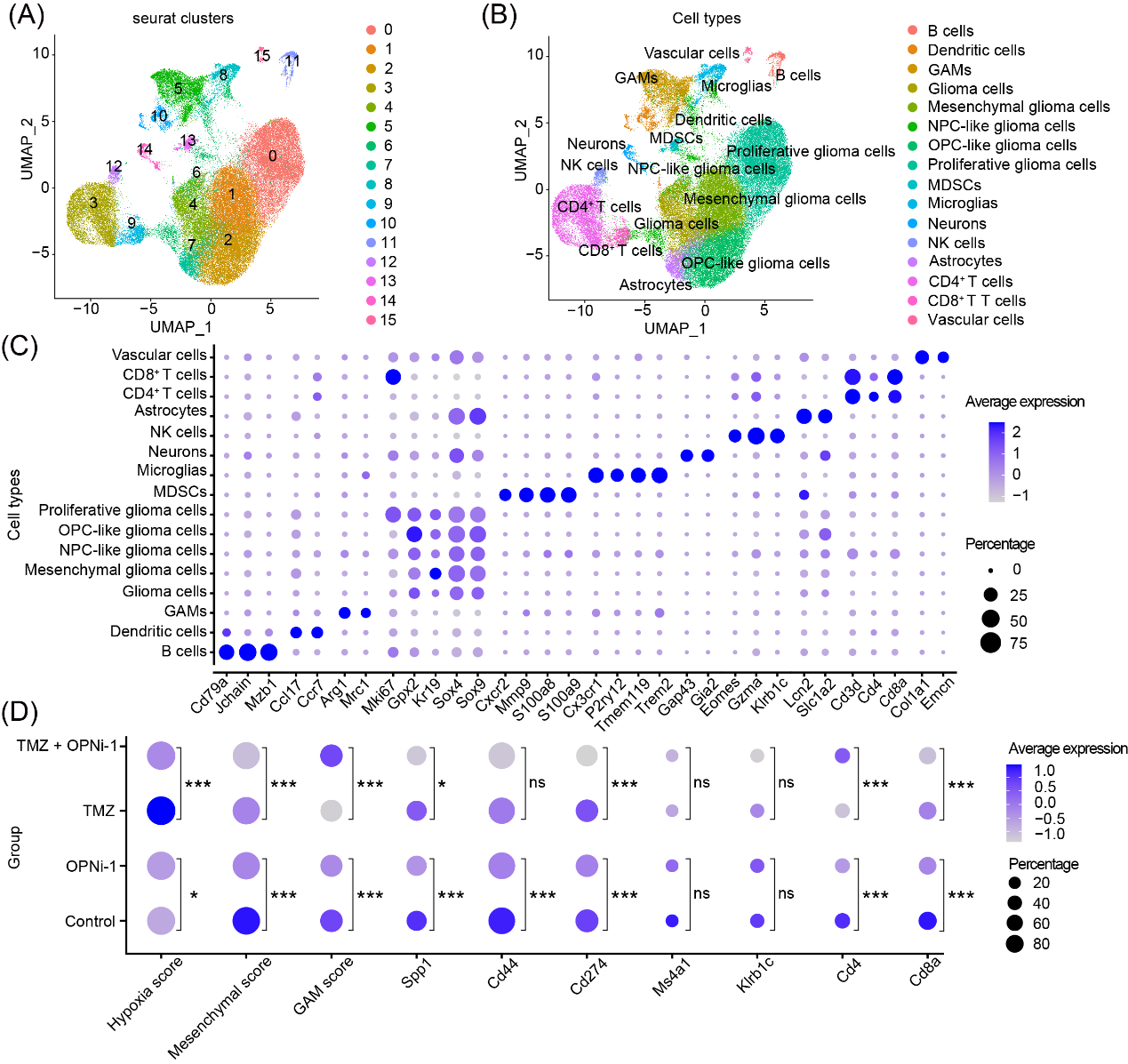


**Supplementary Figure S23. OPNi-1 influences glioma intratumoral immune microenvironment.**

(A) UMAP plot of scRNA-seq data from 12 glioma samples derived from C57BL/6J mice across four treatment groups (control, treatment with PBS; OPNi-1, treatment with OPNi-1 alone; TMZ, treatment with TMZ alone; TMZ + OPNi-1, combined treatment with TMZ and OPNi-1, *n* = 3 per group). Cells were grouped into distinct clusters by unsupervised clustering.

(B) UMAP plot of mice glioma scRNA-seq data with clusters annotated into 16 cell types based on canonical marker genes.

(C) Dot plot displaying the expression of representative marker genes for all cell types identified in mice glioma samples. The size of the dots reflects the proportion of cells expressing each gene in each cell type, while the color denotes the average expression level.

(D) Dot plot showing hypoxia, mesenchymal and GAM scores, as well as the expression of Spp1, Cd44, Cd274, Ms4a1, Klrb1c, Cd4 and Cd8a in mice gliomas from the four treatment groups. Dot size indicates the percentage of cells expressing the corresponding gene or score, and dot color denotes the scaled average expression level.

ns, not significant, **P* < 0.05, ****P* < 0.001.

Abbreviations: PBS, phosphate buffered saline; TMZ, temozolomide; Cd274, programmed cell death ligand 1; Cd44, cluster of differentiation 44; OPN, osteopontin; OPNi-1, OPN expression inhibitor 1; NK, nature killer; MDSC, myeloid-derived suppressor cell; OPC, oligodendrocyte precursor cell; NPC, neural progenitor cell; Cd163, cluster of differentiation 163; GAM, glioma associated macrophage; Ms4a1, membrane spanning 4-domains A1; Klrb1c, killer cell lectin-like receptor subfamily B member 1C; Cd4, cluster of differentiation 4; Cd8a, Cd8 subunit alpha.

**Supplementary Table S1. Sequences of siRNAs used in this study.**

| **siRNA** | **Sequence (5’-3’)** | | **Application** |
| --- | --- | --- | --- |
| siWDR5-1 | sense | GCUCAGAGGAUAACCUUGUTT | Gene knockdown |
|  | anti-sense | ACAAGGUUAUCCUCUGAGCTT |  |
| siWDR5-2 | sense | CCCAGUCCAACCUUAUUGUTT | Gene knockdown |
|  | anti-sense | ACAAUAAGGUUGGACUGGGTT |  |
| siWDR5-3 | sense | GUCGUCAGAUUCUAACCUUTT | Gene knockdown |
|  | anti-sense | AAGGUUAGAAUCUGACGACTT |  |
| siSPP1-1 | sense | GGAGUUGAAUGGUGCAUACAATT | Gene knockdown |
|  | anti-sense | UUGUAUGCACCAUUCAACUCCTT |  |
| siSPP1-2 | sense | GCAUCUUCUGAGGUCAAUUAATT | Gene knockdown |
|  | anti-sense | UUAAUUGACCUCAGAAGAUGCTT |  |
| siSPP1-3 | sense | UUAAUUGACCUCAGAAGAUGCTT | Gene knockdown |
|  | anti-sense | UGUUUAACUGGUAUGGCACTT |  |

**Supplementary Table S2. Sequences of shRNAs used in this study.**

| **shRNA** | **Sequence (5’-3’)** | **Application** |
| --- | --- | --- |
| shSPP1-1 | GGCTGATTCTGGAAGTTCTGA | Gene knockdown |
| shSPP1-2 | GCCATGAATTTCACAGCCATG | Gene knockdown |
| shSPP1-3 | ACCCTTCCAAGTAAGTCCAAC | Gene knockdown |
| shCD44-1 | GACCTCTGCAAGGCTTTCAAT | Gene knockdown |
| shCD44-2 | CTGCCGCTTTGCAGGTGTATT | Gene knockdown |
| shCD44-3 | GAGCATCGGATTTGAGACCTG | Gene knockdown |

**Supplementary Table S3. Lentiviral vectors used in this study.**

| **Lentiviral vector** | **Description of vector** | **Application** |
| --- | --- | --- |
| GV493 | hU6-MCS-CBh-gcGFP-IRES-puromycin | Knockdown of SPP1, knockdown of CD44 |
| GV721 | CMV enhancer-MCS-3FLAG-EF1a-firefly_Luciferase-SV40-Puromycin | Over expression of SPP1 |

**Supplementary Table S4. Sequences of primers used in this study.**

| **Primer** | **Sequence (5’-3’)** | | **Application** |
| --- | --- | --- | --- |
| ACTB | Forward | CACCATTGGCAATGAGCGGTTC | qPCR |
|  | Reverse | AGGTCTTTGCGGATGTCCACGT |  |
| CD44 | Forward | CTGCCGCTTTGCAGGTGTA | qPCR |
|  | Reverse | CATTGTGGGCAAGGTGCTATT |  |
| CD163 | Forward | ACATAGATCATGCATCTGTCATTTG | qPCR |
|  | Reverse | CATTCTCCTTGGAATCTCACTTCTA |  |
| IL-10 | Forward | TCCCTGTGAAAACAAGAGCA | qPCR |
|  | Reverse | ATAGAGTCGCCACCCTGATG |  |
| TGF-β | Forward | GAGCCCAAGGGCTACCAT | qPCR |
|  | Reverse | CTTGCGGCCCACGTAGTA |  |
| SPP1 | Forward | AGGTCTTTGCGGATGTCCACGT | qPCR |
|  | Reverse | CAGGTCTGCGAAACTTCTTAGAT |  |
| SPP1 promotor | Forward | AAACGCCGACCAAGGTACAG | ChIP-PCR |
|  | Reverse | GCATCGGTGGTTTCCGTTCT |  |
| CD274 promotor | Forward | CTAGAAGTTCAGCGCGGGAT | ChIP-PCR |
|  | Reverse | ATCGGCGGAAGCTTTCAGTT |  |

Abbreviations: qPCR, quantitative real-time PCR; ChIP-PCR, chromatin immunoprecipitation-PCR.

**Supplementary Table S5. Primary antibodies used in this study.**

| **Primary antibody** | **Supplier** | **Catalog No.** | **Dilution (application)** |
| --- | --- | --- | --- |
| Akt | Abcam | ab185633 | 1:2000 (WB) |
| CA9 | Proteintech Group | 11071-1-AP | 1:200 (mIHC), 1:500 (IHC) |
| CD163 | Abcam | ab182422 | 1:500 (IHC), 1:500 (mIHC) |
| CD20 | Abcam | ab64088 | 1:200 (mIHC) |
| CD4 | Abcam | ab183685 | 1:200 (mIHC) |
| CD44 (mouse) | Cell signaling Technology | 156-3L11 | 1:500 (WB), 1:200 (ICH),  1:100 (IF) |
| CD44 (Rabbit) | Abcam | ab243894 | 1:200 (IHC) |
| CD8a | Abcam | ab316778 | 1:200 (mIHC) |
| EFEMP1 | Abacm | ab228797 | 1:1000 (WB) |
| ERK1/2 | Abcam | ab184699 | 1:2500 (WB) |
| F4/80 | Abcam | ab300421 | 1:200 (mIHC) |
| H3K4me3 | Active Motif | 61379 | 1:500 (WB),1:200 (IF) |
| H3K9me3 | Active Motif | 39161 | 1:500 (WB),1:200 (IF) |
| HIF-1α | Proteintech Group | 20960-1-AP | 1:500 (WB), 1:200 (IF) |
| Histone H3 | Lablead | H0101 | 1:300 (WB) |
| Iba1 | Abcam | ab178847 | 1:200 (mIHC) |
| K3K27me3 | Active Motif | 61017 | 1:500 (WB),1:200 (IF) |
| Ki-67 | Proteintech Group | 27309-1-AP | 1:500 (IHC) |
| NF-κB (p65) | Abcam | ab16502 | 1:5000 (WB); 1:500 (IF) |
| NK1.1 | Cell signaling technology | E6Y9G | 1:200 (mIHC) |
| Osteopontin | Abcam | ab283656 | 1:2000 (WB), 1:500 (IHC),  1:500 (mIHC), 1:200 (IF) |
| p-AKT | Abcam | ab192623 | 1:1000 (WB) |
| PD-L1 | Abcam | ab213524 | 1:1000 (WB), 1:200 (IHC), 1:200 (mIHC) |
| p-ERK1/2 | Abcam | ab223500 | 1:1000 (WB) |
| PI3K (p85) | Cell Signaling Technology | 4257S | 1:500 (WB) |
| p-NF-κB (p65) | Abcam | ab76302 | 1:1000 (WB) |
| p-PI3K (p85) | Cell Signaling Technology | 17366S | 1:500 (WB) |
| WDR5 | Abcam | 15544-1-AP | 1:500 (WB) |
| YKL-40 | ThermoFisher | PA5-37357 | 1:2000 (WB) |
| β-actin | Proteintech Group | 66009-1-Ig | 1:2000 (WB) |

Abbreviations: mIHC, multi-immunohistochemistry; WB, Western blotting; IF, immunofluorescence; IHC, immunohistochemistry.

**Supplementary Table S6. DEGs in hypoxia-induced macrophages compared with normoxia.**

| **DEG** | **Expression** |
| --- | --- |
| ALDOC | Upregulated |
| FCGR2C | Upregulated |
| ANGPTL4 | Upregulated |
| ZNF395 | Upregulated |
| SEMA4B | Upregulated |
| FANCE | Upregulated |
| JAML | Upregulated |
| CALHM6 | Upregulated |
| CXCR4 | Upregulated |
| ETV3L | Upregulated |
| GPRC5C | Upregulated |
| RIN3 | Upregulated |
| NUPR1 | Upregulated |
| CD70 | Upregulated |
| PIGR | Upregulated |
| AK4 | Upregulated |
| PLOD2 | Upregulated |
| P4HA1 | Upregulated |
| FCRLB | Upregulated |
| DDIT4 | Upregulated |
| RGS16 | Upregulated |
| IRF8 | Upregulated |
| TMEM51 | Upregulated |
| ADRA2C | Upregulated |
| CYTIP | Upregulated |
| HIF1A-AS2 | Upregulated |
| ASCL2 | Upregulated |
| EFEMP2 | Upregulated |
| FCRLA | Upregulated |
| EEF1A2 | Upregulated |
| ATP8B3 | Upregulated |
| KDM3A | Upregulated |
| PDGFB | Upregulated |
| AC007663.2 | Upregulated |
| LPO | Upregulated |
| S100A10 | Upregulated |
| APBB1 | Upregulated |
| NAPSB | Upregulated |
| TBC1D30 | Upregulated |
| RGCC | Upregulated |
| AL360181.2 | Upregulated |
| LINC01127 | Upregulated |
| HILPDA | Upregulated |
| TBC1D22A | Upregulated |
| KIF26A | Upregulated |
| ADM | Upregulated |
| TIFAB | Upregulated |
| BNIP3 | Upregulated |
| TCAF2 | Upregulated |
| ANKRD37 | Upregulated |
| BNIP3L | Upregulated |
| LDHA | Upregulated |
| C16orf74 | Upregulated |
| AK4P1 | Upregulated |
| ADSSL1 | Upregulated |
| HIST1H2BD | Upregulated |
| RASSF7 | Upregulated |
| WDR66 | Upregulated |
| AC004865.2 | Upregulated |
| SHISA8 | Upregulated |
| FCGR2B | Upregulated |
| PNCK | Upregulated |
| SPP1 | Upregulated |
| SLC2A3 | Upregulated |
| CERCAM | Upregulated |
| SYTL1 | Upregulated |
| SLAMF9 | Upregulated |
| SLC2A5 | Upregulated |
| MXI1 | Upregulated |
| LUCAT1 | Upregulated |
| CDHR5 | Upregulated |
| TM7SF2 | Upregulated |
| AL357992.1 | Upregulated |
| AP002954.1 | Upregulated |
| NOXA1 | Upregulated |
| TMEM45A | Upregulated |
| EGLN3 | Upregulated |
| HSPA7 | Upregulated |
| CERNA2 | Upregulated |
| SLC2A1 | Upregulated |
| TMEM91 | Upregulated |
| ENO2 | Upregulated |
| SCN5A | Upregulated |
| TENT5C | Upregulated |
| ZFYVE28 | Upregulated |
| RGS13 | Upregulated |
| ICAM5 | Upregulated |
| ANGPTL6 | Upregulated |
| LSP1 | Upregulated |
| RAB17 | Upregulated |
| MIR210HG | Upregulated |
| SULF2 | Upregulated |
| SLC6A8 | Upregulated |
| GBE1 | Upregulated |
| OR7E47P | Upregulated |
| NDRG1 | Upregulated |
| SPAG4 | Upregulated |
| MPPED1 | Upregulated |
| AGXT | Upregulated |
| C6orf223 | Upregulated |
| ENHO | Upregulated |
| LIPG | Upregulated |
| RAB26 | Upregulated |
| NARF | Upregulated |
| MSMO1 | Upregulated |
| CASS4 | Downregulated |
| PTPRK | Downregulated |
| NECTIN3 | Downregulated |
| SLC39A14 | Downregulated |
| CHAC1 | Downregulated |
| SLC7A11 | Downregulated |
| URB1 | Downregulated |
| FBN2 | Downregulated |
| FAM84B | Downregulated |
| MYORG | Downregulated |
| SLC7A1 | Downregulated |
| HIF1A | Downregulated |
| PSAT1 | Downregulated |
| GTF2IP1 | Downregulated |
| ITGA6 | Downregulated |

**Supplementary Table S7 Univariate and multivariate Cox analyses of overall survival in IDH-WT glioma patients from the** **TCGA cohort.**

| **Variable** | **Univariate analysis** | | **Multivariate analysis** | |
| --- | --- | --- | --- | --- |
|  | **HR**  **(95% CI)** | ***P* value** | **HR**  **(95% CI)** | ***P* value** |
| SPP1 expression  (continuous) | 1.285  (1.145-1.443) | < 0.001 | 1.243  (1.093-1.413) | <0.001 |
| Age at diagnosis  (continuous) | 1.057  (1.039-1.075) | < 0.001 | 1.053  (1.032-1.074) | 0.001 |
| Gender  (female vs male) | 0.867  (0.712-1.058) | 0.160 | 0.952  (0.773-1.172) | 0.641 |
| WHO grade  (grade 4 vs grade 2/3) | 2.508  (1.647 -3.820) | < 0.001 | 1.164  (0.718-1.886) | 0.537 |
| MGMT methylation  (methylated vs unmethylated) | 1.056  (0.870-1.282) | 0.582 | 0.931  (0.757-1.145) | 0.497 |

Abbreviations: IHD-WT, isocitrate dehydrogenase wild type; SPP1, secreted phosphoprotein 1; MGMT, O-6-methylguanine-DNA methyltransferase. WHO, World Health Organization; HR, hazard ratio; CI, confidence interval; TCGA, the Cancer Genome Atlas.

**Supplementary Table S8 Univariate and multivariate Cox analyses of overall survival in IDH-WT glioma patients from the CGGA cohort.**

| **Variable** | **Univariate analysis** | | **Multivariate analysis** | |
| --- | --- | --- | --- | --- |
|  | **HR**  **(95% CI)** | ***P* value** | **HR**  **(95% CI)** | ***P* value** |
| SPP1 expression  (continuous) | 1.263  (1.108-1.439) | < 0.001 | 1.225 (1.090-1.445) | 0.002 |
| Age at diagnosis  (continuous) | 1.010  (0.991-1.028) | 0.279 | 1.053 (1.032-1.074) | 0.001 |
| Gender  (female vs. male) | 0.876  (0.702-1.093) | 0.242 | 0.979 (0.768-1.248) | 0.864 |
| WHO grade  (grade 4 vs. grade 2/3) | 2.134  (1.287-3.538) | 0.003 | 1.778  (1.061-2.980) | 0.029 |
| MGMT methylation  (methylated vs. unmethylated) | 0.811  (0.649-1.012) | 0.063 | 0.761  (0.606-0.9578) | 0.020 |

Abbreviations: IHD-WT, isocitrate dehydrogenase wild type; SPP1, secreted phosphoprotein 1; MGMT, O-6-methylguanine-DNA methyltransferase. WHO, World Health Organization; HR, hazard ratio; CI, Confidence interval; TCGA, the Cancer Genome Atlas.

**Supplementary Table S9. Consistently upregulated DEGs in BNI_2-4, BNI_1-3, and U87-MG cells cultured with hypoxic CM.**

| **DEG** | **Expression** |
| --- | --- |
| SERPINE1 | Upregulated |
| TGFBI | Upregulated |
| IGFBP3 | Upregulated |
| F3 | Upregulated |
| DEPP1 | Upregulated |
| ADM | Upregulated |
| IL1B | Upregulated |
| LOXL3 | Upregulated |
| PLAUR | Upregulated |
| C3 | Upregulated |
| MT1X | Upregulated |
| ALDOC | Upregulated |
| SLC16A3 | Upregulated |
| LOXL2 | Upregulated |
| NDRG1 | Upregulated |
| CYBA | Upregulated |
| GADD45B | Upregulated |
| IL6 | Upregulated |
| SCG5 | Upregulated |
| GPR176 | Upregulated |
| CSF2 | Upregulated |
| SLC2A1 | Upregulated |
| LOX | Upregulated |
| SRGN | Upregulated |
| BNIP3 | Upregulated |
| P4HA2 | Upregulated |
| CSF3 | Upregulated |
| PARVB | Upregulated |
| ITGB3 | Upregulated |
| ITGA5 | Upregulated |
| HILPDA | Upregulated |
| NRXN2 | Upregulated |
| PI3 | Upregulated |
| ENO2 | Upregulated |
| SYTL3 | Upregulated |
| MT2A | Upregulated |
| CLEC11A | Upregulated |
| RASSF4 | Upregulated |
| ADPRHL1 | Upregulated |
| RIPOR3 | Upregulated |
| TBX3 | Upregulated |
| EGR2 | Upregulated |
| C1QTNF1 | Upregulated |
| DUSP1 | Upregulated |
| PLOD2 | Upregulated |
| ANGPTL4 | Upregulated |
| CDH13 | Upregulated |
| IL32 | Upregulated |
| HK2 | Upregulated |
| CXCL8 | Upregulated |
| METRNL | Upregulated |
| BASP1 | Upregulated |
| IER3 | Upregulated |
| ARTN | Upregulated |
| PTX3 | Upregulated |
| ZP1 | Upregulated |
| SOD2 | Upregulated |
| TMEM255B | Upregulated |
| PPP2R5B | Upregulated |
| LBH | Upregulated |
| TNFRSF1B | Upregulated |
| TUBB6 | Upregulated |
| TMEM45A | Upregulated |
| UPP1 | Upregulated |
| CA9 | Upregulated |
| CD82 | Upregulated |
| JUN | Upregulated |
| MFAP2 | Upregulated |
| MAFF | Upregulated |
| KCTD11 | Upregulated |
| NRN1 | Upregulated |
| MT3 | Upregulated |
| BEND5 | Upregulated |
| SMTN | Upregulated |
| TIMP1 | Upregulated |
| CRACR2B | Upregulated |
| ZNF503 | Upregulated |
| S100A10 | Upregulated |
| AP000769.1 | Upregulated |
| SNHG18 | Upregulated |
| GNA15 | Upregulated |
| IGFBP6 | Upregulated |
| FERMT3 | Upregulated |
| PTPRN2 | Upregulated |
| HLA-B | Upregulated |
| FIBCD1 | Upregulated |
| PCBP3 | Upregulated |
| GPR68 | Upregulated |
| MTMR4 | Upregulated |
| TNIP1 | Upregulated |
| DPYSL4 | Upregulated |
| 3-3月 | Upregulated |
| GLTP | Upregulated |
| SLC2A5 | Upregulated |
| TREM1 | Upregulated |
| TFPI2 | Upregulated |
| VEGFA | Upregulated |
| SHANK1 | Upregulated |
| CTSS | Upregulated |
| PLXNB3 | Upregulated |
| NDNF | Upregulated |
| JPH2 | Upregulated |
| FAM162A | Upregulated |
| GAPDH | Upregulated |
| AC079949.2 | Upregulated |
| PCSK5 | Upregulated |
| EGLN3 | Upregulated |
| RRAS | Upregulated |
| ANXA5 | Upregulated |
| NRIP3 | Upregulated |
| KRT79 | Upregulated |
| SNAI2 | Upregulated |
| APLN | Upregulated |
| ENO1 | Upregulated |
| AP002852.1 | Upregulated |
| SDC4 | Upregulated |
| LRIG1 | Upregulated |
| TMEM132B | Upregulated |
| ITGB1-DT | Upregulated |
| ABLIM3 | Upregulated |
| GLUL | Upregulated |
| PNRC1 | Upregulated |
| SNX33 | Upregulated |
| PGK1 | Upregulated |
| EIF4EBP1 | Upregulated |
| SAA1 | Upregulated |
| JAM2 | Upregulated |
| FOXL1 | Upregulated |
| IQCG | Upregulated |
| CLEC2B | Upregulated |
| ANXA8 | Upregulated |
| SERINC2 | Upregulated |
| ARHGAP22 | Upregulated |
| MSANTD3 | Upregulated |
| TMSB10 | Upregulated |
| PPP1R3C | Upregulated |
| GDNF | Upregulated |
| LMCD1 | Upregulated |
| ATF3 | Upregulated |
| LINC01411 | Upregulated |
| NFKBIA | Upregulated |
| C4orf3 | Upregulated |
| WNT5A | Upregulated |
| NFIL3 | Upregulated |
| ANKRD37 | Upregulated |
| GYS1 | Upregulated |
| IL11 | Upregulated |
| PTPRH | Upregulated |
| VKORC1 | Upregulated |
| BDKRB2 | Upregulated |
| CITED2 | Upregulated |
| RAB20 | Upregulated |
| ZNF175 | Upregulated |
| RPSAP52 | Upregulated |
| SYNDIG1 | Upregulated |
| FOSL2 | Upregulated |
| UNC119 | Upregulated |
| TLN1 | Upregulated |
| LRP1 | Upregulated |
| SIRPA | Upregulated |
| AK2 | Upregulated |
| CEBPB | Upregulated |
| SRD5A3 | Upregulated |
| CABP1 | Upregulated |
| MFSD2A | Upregulated |
| KCMF1 | Upregulated |
| POU2F2 | Upregulated |
| TMOD1 | Upregulated |
| SPAG4 | Upregulated |
| WFDC3 | Upregulated |
| NGLY1 | Upregulated |
| RELB | Upregulated |
| PAQR7 | Upregulated |
| PFKFB4 | Upregulated |
| UBA6-AS1 | Upregulated |
| RPL23AP82 | Upregulated |
| AFAP1L1 | Upregulated |
| SH3D21 | Upregulated |
| HLA-E | Upregulated |
| UCN2 | Upregulated |
| MCF2L | Upregulated |
| GOLGA7B | Upregulated |
| P4HB | Upregulated |
| NAGS | Upregulated |
| GALNT18 | Upregulated |
| STEAP3 | Upregulated |
| BHLHE41 | Upregulated |
| RCAN1 | Upregulated |
| LRRFIP1 | Upregulated |
| TNNT1 | Upregulated |
| SYDE1 | Upregulated |
| SERTAD1 | Upregulated |
| TGIF1 | Upregulated |
| AC079949.1 | Upregulated |
| MPP2 | Upregulated |
| C10orf90 | Upregulated |
| SLC6A8 | Upregulated |
| UBE2E2 | Upregulated |
| ALDOA | Upregulated |
| TRPM2 | Upregulated |
| DACT1 | Upregulated |
| ICAM5 | Upregulated |
| SEC14L2 | Upregulated |
| SYCE1L | Upregulated |
| PFKFB3 | Upregulated |
| PCDH1 | Upregulated |
| ARID3A | Upregulated |
| S100A11 | Upregulated |
| EPS8L1 | Upregulated |
| COL7A1 | Upregulated |
| PGM1 | Upregulated |
| SIK1B | Upregulated |
| IRF7 | Upregulated |
| SAP30 | Upregulated |
| MAP2K1 | Upregulated |
| LINC00887 | Upregulated |
| EMP1 | Upregulated |
| CENPBD1P1 | Upregulated |
| AL591845.1 | Upregulated |
| RNF122 | Upregulated |
| DDX41 | Upregulated |
| PGAM1 | Upregulated |
| EPOR | Upregulated |
| MYO1D | Upregulated |
| CSRP2 | Upregulated |
| SAMD14 | Upregulated |
| TMEM132A | Upregulated |
| CRYBB2P1 | Upregulated |
| RNF144B | Upregulated |
| ISG20 | Upregulated |
| HSD11B1 | Upregulated |
| SLC45A1 | Upregulated |
| BDKRB1 | Upregulated |
| NLRP3 | Upregulated |
| C16orf74 | Upregulated |
| RPL29P19 | Upregulated |
| RPS28P7 | Upregulated |
| IMP3 | Upregulated |
| MIR210HG | Upregulated |
| LAPTM5 | Upregulated |
| GLRX | Upregulated |
| PPFIA4 | Upregulated |
| SIK1 | Upregulated |
| FAM219A | Upregulated |
| COL5A1 | Upregulated |
| MAP7D2 | Upregulated |
| TWIST1 | Upregulated |
| SNTA1 | Upregulated |
| NOG | Upregulated |
| AC009549.1 | Upregulated |
| RPS28 | Upregulated |
| SSR4 | Upregulated |
| KDM7A-DT | Upregulated |
| TBC1D22A | Upregulated |
| CCL20 | Upregulated |
| ZNF503-AS2 | Upregulated |
| AMPD3 | Upregulated |
| JMJD6 | Upregulated |
| PPP1R3G | Upregulated |
| PLOD1 | Upregulated |
| MSC | Upregulated |
| AC079466.1 | Upregulated |
| CALCOCO1 | Upregulated |
| AC004585.1 | Upregulated |
| AMPD2 | Upregulated |
| SCNN1B | Upregulated |
| PRSS3 | Upregulated |
| PLIN2 | Upregulated |
| DCUN1D3 | Upregulated |
| EEF1A2 | Upregulated |
| TIE1 | Upregulated |
| ANXA8L1 | Upregulated |
| AC003092.1 | Upregulated |
| CCDC107 | Upregulated |
| MYDGF | Upregulated |
| LUCAT1 | Upregulated |
| QSOX1 | Upregulated |
| HS3ST3B1 | Upregulated |
| ZC3H12A | Upregulated |
| DAB2 | Upregulated |
| RABEP2 | Upregulated |
| LINC01588 | Upregulated |
| PHPT1 | Upregulated |
| TBC1D9B | Upregulated |
| GYPC | Upregulated |
| PFKP | Upregulated |
| DBH-AS1 | Upregulated |
| AP000695.2 | Upregulated |
| LAMB3 | Upregulated |
| TNS1 | Upregulated |
| SEC61G | Upregulated |
| NCK2 | Upregulated |
| INSIG2 | Upregulated |
| RASSF1 | Upregulated |
| FKBP8 | Upregulated |
| VSIR | Upregulated |
| AC097534.2 | Upregulated |
| RELA | Upregulated |
| UBXN6 | Upregulated |
| AC093673.1 | Upregulated |
| TGFA | Upregulated |
| PLCH2 | Upregulated |
| HM13 | Upregulated |
| AL583856.1 | Upregulated |
| CEBPD | Upregulated |
| RHOB | Upregulated |
| OPTN | Upregulated |
| SERPINB2 | Upregulated |
| MKRN1 | Upregulated |
| STK10 | Upregulated |
| TNFRSF10D | Upregulated |
| LIF | Upregulated |
| RPL23AP7 | Upregulated |
| EHD2 | Upregulated |
| IGFBP4 | Upregulated |
| STARD10 | Upregulated |
| BCL2A1 | Upregulated |
| RIMKLA | Upregulated |
| TMED9 | Upregulated |
| IL1A | Upregulated |
| RPLP0P2 | Upregulated |
| HELZ2 | Upregulated |
| EVA1B | Upregulated |
| ERCC1 | Upregulated |
| PLEK2 | Upregulated |
| CD99 | Upregulated |
| SH3GL1 | Upregulated |
| HCLS1 | Upregulated |
| FADS3 | Upregulated |
| SSR2 | Upregulated |
| ZNF395 | Upregulated |
| HLA-A | Upregulated |
| GUK1 | Upregulated |
| AC022092.1 | Upregulated |
| ZNF581 | Upregulated |
| PDE2A | Upregulated |
| SLC16A6 | Upregulated |
| IL1RN | Upregulated |
| RRAGA | Upregulated |
| SLC38A5 | Upregulated |
| DNAJB2 | Upregulated |
| CLK3 | Upregulated |
| CCNG2 | Upregulated |
| SPACA6 | Upregulated |
| BRSK1 | Upregulated |
| BID | Upregulated |
| CD6 | Upregulated |
| BCAP31 | Upregulated |
| APBA3 | Upregulated |
| INHBA | Upregulated |
| DOCK5 | Upregulated |
| WDR13 | Upregulated |
| STARD13 | Upregulated |
| CES1 | Upregulated |
| WDR45B | Upregulated |
| RSPH9 | Upregulated |
| CSRNP1 | Upregulated |
| AC099552.1 | Upregulated |
| SYP | Upregulated |
| RNF227 | Upregulated |
| APOH | Upregulated |
| UPRT | Upregulated |
| MT1L | Upregulated |
| PRDM1 | Upregulated |
| SLC6A6 | Upregulated |
| TNFSF13B | Upregulated |
| GBE1 | Upregulated |
| WDR66 | Upregulated |
| ITGAX | Upregulated |
| IRS2 | Upregulated |
| ALK | Upregulated |
| HIF3A | Upregulated |
| EMD | Upregulated |
| BX640514.2 | Upregulated |
| BTG1 | Upregulated |
| FTH1P7 | Upregulated |
| LINC01133 | Upregulated |
| CD300A | Upregulated |
| ARHGAP30 | Upregulated |
| HLA-C | Upregulated |
| MME | Upregulated |
| CLDN7 | Upregulated |
| SERPINA1 | Upregulated |
| NFKB2 | Upregulated |
| ERO1A | Upregulated |
| ALKBH5 | Upregulated |
| ACADVL | Upregulated |
| C12orf57 | Upregulated |
| GDI1 | Upregulated |
| CHIC2 | Upregulated |
| POFUT2 | Upregulated |
| C11orf96 | Upregulated |
| NFKBIE | Upregulated |
| KANK3 | Upregulated |
| PCAT6 | Upregulated |
| PAG1 | Upregulated |
| P4HA1 | Upregulated |
| ARID5A | Upregulated |
| FUT11 | Upregulated |
| MMP9 | Upregulated |
| ADRA1B | Upregulated |
| NECAB2 | Upregulated |
| NAA80 | Upregulated |
| PHF1 | Upregulated |
| SHB | Upregulated |
| BCL11B | Upregulated |
| PROSER2 | Upregulated |
| SAMD4A | Upregulated |
| IL1RAP | Upregulated |
| MYOSLID | Upregulated |
| PTGS2 | Upregulated |
| VPS37C | Upregulated |
| MGARP | Upregulated |
| CLSTN1 | Upregulated |
| BNIP3L | Upregulated |
| ERF | Upregulated |
| METTL26 | Upregulated |
| PMEPA1 | Upregulated |
| SBNO2 | Upregulated |
| ING1 | Upregulated |
| NOP53 | Upregulated |
| GPSM3 | Upregulated |
| TMEM51 | Upregulated |
| TNKS1BP1 | Upregulated |
| NCKIPSD | Upregulated |
| GDNF-AS1 | Upregulated |
| FEZF1-AS1 | Upregulated |
| AP000695.1 | Upregulated |
| ANG | Upregulated |
| PSD4 | Upregulated |
| ARHGAP45 | Upregulated |
| ACAP1 | Upregulated |
| MMP19 | Upregulated |
| FTH1P23 | Upregulated |
| DBH | Upregulated |
| SLAMF9 | Upregulated |
| SERPING1 | Upregulated |
| JUNB | Upregulated |
| TRIM8 | Upregulated |
| PREP | Upregulated |
| FAM3A | Upregulated |
| ELL | Upregulated |
| VLDLR | Upregulated |
| LDHA | Upregulated |
| LTBP3 | Upregulated |
| HMGN2P46 | Upregulated |
| CCL3 | Upregulated |
| ADAMTS16 | Upregulated |
| TNFAIP3 | Upregulated |
| PPP1R3B | Upregulated |
| MZF1 | Upregulated |
| SYT7 | Upregulated |
| TFF2 | Upregulated |
| MRGPRX3 | Upregulated |
| VPS37D | Upregulated |
| MYO1B | Upregulated |
| LZTS2 | Upregulated |
| ACKR3 | Upregulated |
| IDH3G | Upregulated |
| PKP1 | Upregulated |
| AC012085.1 | Upregulated |
| AC090197.1 | Upregulated |
| SLC29A4 | Upregulated |
| ZBTB17 | Upregulated |
| KISS1R | Upregulated |
| ASL | Upregulated |
| ABTB1 | Upregulated |
| STAC2 | Upregulated |
| MAP3K8 | Upregulated |
| AL118516.1 | Upregulated |
| YKT6 | Upregulated |
| MEX3B | Upregulated |
| SARDH | Upregulated |
| MRGBP | Upregulated |
| TNIP2 | Upregulated |
| ARHGEF5 | Upregulated |
| C8orf58 | Upregulated |
| AC080023.1 | Upregulated |
| TBC1D8B | Upregulated |
| RPL12P14 | Upregulated |
| UBAP1 | Upregulated |
| RNF187 | Upregulated |
| CCDC9 | Upregulated |
| CCL3L1 | Upregulated |
| AC009139.1 | Upregulated |
| SLC27A1 | Upregulated |
| AC078819.1 | Upregulated |
| PFKL | Upregulated |
| UBE2E1 | Upregulated |
| HLA-H | Upregulated |
| CIART | Upregulated |
| CSNK1E | Upregulated |
| SIGIRR | Upregulated |
| SIAH2 | Upregulated |
| BNIP3P1 | Upregulated |
| CCDC102A | Upregulated |
| LHX5 | Upregulated |
| DPP9 | Upregulated |
| IFFO2 | Upregulated |
| AC025580.2 | Upregulated |
| CDK5R2 | Upregulated |
| STEAP3-AS1 | Upregulated |
| NIM1K | Upregulated |
| EGLN1 | Upregulated |
| HCAR2 | Upregulated |
| SHROOM4 | Upregulated |
| FRMD3 | Upregulated |
| TGFB1 | Upregulated |
| COL6A2 | Upregulated |
| AJ239328.1 | Upregulated |
| KDM4B | Upregulated |
| P2RY8 | Upregulated |
| SLC35E1 | Upregulated |
| SH2B2 | Upregulated |
| ABL1 | Upregulated |
| PRKCB | Upregulated |
| MFGE8 | Upregulated |
| ROPN1L | Upregulated |
| RTN2 | Upregulated |
| PTGS1 | Upregulated |
| IL24 | Upregulated |
| HLA-V | Upregulated |
| LPO | Upregulated |
| AC008659.1 | Upregulated |
| KLF7 | Upregulated |
| IRF2BP2 | Upregulated |
| NAB2 | Upregulated |
| GPT2 | Upregulated |
| SEC61A1 | Upregulated |
| IGSF8 | Upregulated |
| AC245297.3 | Upregulated |
| AC144530.1 | Upregulated |
| AC044787.1 | Upregulated |
| MARK4 | Upregulated |
| TMEM191A | Upregulated |
| LONP1 | Upregulated |
| GCGR | Upregulated |
| CNOT8 | Upregulated |
| C3orf58 | Upregulated |
| CIC | Upregulated |
| PLA2G4D | Upregulated |
| HDHD5 | Upregulated |
| MEIS3 | Upregulated |
| NOL3 | Upregulated |
| SLA | Upregulated |
| ISM2 | Upregulated |
| EFNA3 | Upregulated |
| GCSAML | Upregulated |
| ULK1 | Upregulated |
| MYADM | Upregulated |
| RASSF2 | Upregulated |
| AP005264.1 | Upregulated |
| MIB2 | Upregulated |
| MANF | Upregulated |
| TUBB2B | Upregulated |
| MEI1 | Upregulated |
| PPM1J | Upregulated |
| HSPA5 | Upregulated |
| MGC12916 | Upregulated |
| CACNA1B | Upregulated |
| AC010247.2 | Upregulated |
| MMP7 | Upregulated |
| PRKRIP1 | Upregulated |
| DYRK1B | Upregulated |
| AL139220.2 | Upregulated |
| KRT4 | Upregulated |
| AL031595.3 | Upregulated |
| FAM129B | Upregulated |
| ZNRF1 | Upregulated |
| GPR146 | Upregulated |
| EEF1AKMT4 | Upregulated |
| KLF17 | Upregulated |
| TMEM44 | Upregulated |
| TCIRG1 | Upregulated |
| GPR84 | Upregulated |
| CTTNBP2 | Upregulated |
| TNIP3 | Upregulated |
| CXCL1 | Upregulated |
| EPS8L2 | Upregulated |
| GPRC5A | Upregulated |
| PTPRU | Upregulated |
| PRSS53 | Upregulated |
| C7orf61 | Upregulated |
| BEST1 | Upregulated |
| NPR3 | Upregulated |
| AC107021.2 | Upregulated |
| RORA | Upregulated |
| FAM131A | Upregulated |
| PANX1 | Upregulated |
| SESN2 | Upregulated |
| DCN | Upregulated |
| AC244669.1 | Upregulated |
| SELPLG | Upregulated |
| AC254633.1 | Upregulated |
| NKX3-1 | Upregulated |
| NEFM | Upregulated |
| PGF | Upregulated |
| CDYL2 | Upregulated |
| WISP1 | Upregulated |
| ADAM12 | Upregulated |
| EEF1AKMT3 | Upregulated |
| WFDC5 | Upregulated |
| SHC3 | Upregulated |
| C4orf50 | Upregulated |
| MYCL | Upregulated |
| AC010247.1 | Upregulated |
| BMP1 | Upregulated |
| ZNF710 | Upregulated |
| UBTD1 | Upregulated |
| FCRLA | Upregulated |
| ANKRD1 | Upregulated |
| GCKR | Upregulated |
| TLR2 | Upregulated |
| LINC02575 | Upregulated |
| ZNF653 | Upregulated |
| ARRDC2 | Upregulated |
| MAP1LC3B2 | Upregulated |
| LINC02376 | Upregulated |
| SLC11A1 | Upregulated |
| HYAL3 | Upregulated |
| JCAD | Upregulated |
| YPEL3 | Upregulated |
| AC008760.2 | Upregulated |
| OSBPL5 | Upregulated |
| MXI1 | Upregulated |
| AC027559.1 | Upregulated |
| TCAF2 | Upregulated |
| RYR1 | Upregulated |
| ICAM1 | Upregulated |
| NUMBL | Upregulated |
| FAM107B | Upregulated |
| PDE4C | Upregulated |
| FCAR | Upregulated |
| SLC27A3 | Upregulated |
| ENDOG | Upregulated |
| OSCAR | Upregulated |
| AL117329.1 | Upregulated |
| AC103702.2 | Upregulated |
| SERPIND1 | Upregulated |
| SYNPO | Upregulated |
| DACT3 | Upregulated |
| DLG4 | Upregulated |
| B9D2 | Upregulated |
| USP11 | Upregulated |
| SLC24A2 | Upregulated |
| EDN1 | Upregulated |
| NOVA2 | Upregulated |
| TBC1D3L | Upregulated |
| AC026310.3 | Upregulated |
| DNER | Upregulated |
| AC005280.2 | Upregulated |
| DOCK8 | Upregulated |
| AC078883.1 | Upregulated |
| VLDLR-AS1 | Upregulated |
| ABCD1 | Upregulated |
| ZDHHC9 | Upregulated |
| NEDD9 | Upregulated |
| TICAM1 | Upregulated |
| SGIP1 | Upregulated |
| FUT4 | Upregulated |
| HR | Upregulated |
| VGLL2 | Upregulated |
| HSPB7 | Upregulated |
| SLC39A14 | Upregulated |
| CCDC184 | Upregulated |
| AC137932.2 | Upregulated |
| BHLHE40 | Upregulated |
| EFNA4 | Upregulated |
| UPB1 | Upregulated |
| AC002480.1 | Upregulated |
| PTPRM | Upregulated |
| AL031590.1 | Upregulated |
| AL357079.1 | Upregulated |
| ITPKC | Upregulated |
| STBD1 | Upregulated |
| ARRDC3 | Upregulated |
| MYO1E | Upregulated |
| TM4SF19 | Upregulated |
| AC107016.1 | Upregulated |
| AC026310.2 | Upregulated |
| HCAR3 | Upregulated |
| PTPRF | Upregulated |
| BTBD11 | Upregulated |
| DDX3Y | Upregulated |
| GASAL1 | Upregulated |
| C4orf47 | Upregulated |
| LANCL3 | Upregulated |
| MAFK | Upregulated |
| GPR137B | Upregulated |
| RBM44 | Upregulated |
| MAP1S | Upregulated |
| MAP6 | Upregulated |
| IL16 | Upregulated |
| SOWAHC | Upregulated |
| FAM83F | Upregulated |
| AC007448.4 | Upregulated |
| ZC3HAV1L | Upregulated |
| DUOX2 | Upregulated |
| PIK3AP1 | Upregulated |
| FAM229A | Upregulated |
| PRKAG2-AS1 | Upregulated |
| PIWIL2 | Upregulated |
| CACNB3 | Upregulated |
| SAA2 | Upregulated |
| ZSWIM4 | Upregulated |
| TMEM91 | Upregulated |
| TNFSF14 | Upregulated |
| AC136632.1 | Upregulated |
| AL691432.2 | Upregulated |
| FSCN2 | Upregulated |
| MED26 | Upregulated |
| TMEM151B | Upregulated |
| SIGLEC7 | Upregulated |
| RUBCNL | Upregulated |
| WNT5A-AS1 | Upregulated |
| CDCP1 | Upregulated |
| PDGFB | Upregulated |
| MB21D2 | Upregulated |
| RAB17 | Upregulated |
| THCAT158 | Upregulated |
| FAM110C | Upregulated |
| PPP1R16A | Upregulated |
| CCR10 | Upregulated |
| SLC9A1 | Upregulated |
| BIRC3 | Upregulated |
| PRICKLE2 | Upregulated |
| ENKUR | Upregulated |
| NYAP1 | Upregulated |
| NR4A3 | Upregulated |
| NR2F2-AS1 | Upregulated |
| ESR2 | Upregulated |
| CLCNKA | Upregulated |
| SAMSN1 | Upregulated |
| RPL17P50 | Upregulated |
| ALOXE3 | Upregulated |
| KLF9 | Upregulated |
| LCE1F | Upregulated |
| ADAMTS14 | Upregulated |
| FBXO42 | Upregulated |
| LDHAP4 | Upregulated |
| LPAR2 | Upregulated |
| PPP1R13L | Upregulated |
| PPAN | Upregulated |
| PAX8 | Upregulated |
| AC139795.2 | Upregulated |
| AMZ1 | Upregulated |
| P2RY11 | Upregulated |
| TMEM200A | Upregulated |
| ZEB2 | Upregulated |
| PTPRB | Upregulated |
| TCAF2C | Upregulated |
| TPBG | Upregulated |
| LINC01583 | Upregulated |
| FOXN3 | Upregulated |
| WNK4 | Upregulated |
| CAHM | Upregulated |
| MEFV | Upregulated |
| DARS-AS1 | Upregulated |
| TRIM29 | Upregulated |
| SORCS3 | Upregulated |
| KCTD16 | Upregulated |
| FANK1 | Upregulated |
| AC021534.1 | Upregulated |
| ALOX15B | Upregulated |
| AL645608.2 | Upregulated |
| AP000695.3 | Upregulated |
| ADAMTS6 | Upregulated |
| LINC02535 | Upregulated |
| AC022509.3 | Upregulated |
| AC107021.1 | Upregulated |
| LINC00565 | Upregulated |
| AC099548.2 | Upregulated |
| SRMS | Upregulated |
| AL356414.1 | Upregulated |
| KMT2E-AS1 | Upregulated |
| ADAMTS18 | Upregulated |
| SEMA6D | Upregulated |
| PIK3IP1 | Upregulated |
| CLDN14 | Upregulated |
| AK4P1 | Upregulated |
| AC245128.3 | Upregulated |
